# Supplementary material for: Susceptibility of domain experts to color manipulation indicate a need for design principles in data visualization
Source: PLoS One. 2021 Feb 4;16(2):e0246479. doi: 10.1371/journal.pone.0246479 (PMC7861358; doi:10.1371/journal.pone.0246479)
Supplement: S1 File — (PDF) [file pone.0246479.s001.pdf]

Introduction

Dear Colleague,

We invite you cordially to spend ~15 minutes to participate in a worldwide survey on the use of colors in displaying complex data, notably in neuroimaging and geographic information visualization. We aim to understand the connection between the display practice and the interpretation of data both among experts as well as lay persons.

The survey "Color Use in Data Displays" is open both to lay persons as well as to experts that are involved in imaging technology development, research and use, in particular in neuroscience and the geosciences. It is hosted by the Institute of Biomedical Ethics of the University of Zurich, the GIScience Center of the University of Zurich, and the neuropsychology unit of the University Hospital Zurich.

If you choose to participate in the survey, please click the button below and complete the survey in one pass. The next page will outline the study such that you can give informed consent for participation. Thank you very much for your important contribution.

Thank you…

…for participating in the study "Color Use in Data Displays". This survey is performed by the following researchers of the University of Zurich, Switzerland: Dr. Markus Christen (Institute of Biomedical Ethics), Prof. Sara Fabrikant and Gianluca Boo (GIScience Center) and Prof. Peter Brugger (University Hospital Neuropsychology Unit). Please contact us, if you have any questions (christen@ethik.uzh.ch).

The study is performed in agreement with the ethical regulations of the University of Zurich:

- You are not asked to disclose personal information besides basic demographics and professional experience.
- All data are collected anonymously and will be treated as confidential.
- Your responding to this survey is voluntary; you are free to withdraw at any time.
- There are no foreseeable risks to you for participating or declining to participate in this study.
- There are no direct benefits to you as a participant beside that you contribute to a better understanding of the use of colors in data displays.

The number of questions presented to you depends on some of your answers. The "progress bar" may therefore show "jumps" in some cases. **Please use the buttons at the bottom of each page to navigate, and do not use the back button of your browser**.

Accessing the survey is the equivalent of your consent to participate in the study.

We thank you for your time to answer our survey.

General Information

Please indicate some general information about yourself:

What is your gender?

- ◯ Female
- ◯ Male

What is your age?

In which country do you currently work?

Please indicate whether you suffer from any type of color vision deficiencies:

- ◯ no deficiency
- ◯ weak red-green color deficiency
- ◯ complete red-green color deficiency
- ◯ yellow-blue color deficiency
- ◯ complete color deficiency (color blindness)

Do you wear glasses or contact lenses?

◯ Yes

◯ No

Determine your field of expertise

Please indicate, which of the following descriptions best apply to you:

◯ I have an education and/or I work or I have worked in **neuroscience, neurology or a related field** or I work professionally with data (e.g., **neuroimages**) emerging from those fields (e.g., as a science journalist).

◯ I have an education and/or I work or I have worked in the **geosciences, including cartography and geovisualization or a related field** or I work professionally with geographic data (e.g., **GIS, maps**) emerging from those fields (e.g., as a GIS analyst, cartographer, graphics designer).

◯ None of the first two options apply to me.

Exp Neuro rainbow black

Evaluating neuroimages

For the next five questions, we ask you to evaluate images that have been produced using techniques to measure brain activity. But before that, we would like to know your opinion on the following matter:

"The state of brain death equals the death of a person."

Please indicate to what extent you agree with this position:

◯ I completely agree; a brain dead person is dead

◯ I somewhat agree; a brain dead person is probably dead

◯ I somewhat disagree; a brain dead person is probably not dead

◯ I completely disagree; a brain dead person is still alive

◯ I have no opinion on this matter

Evaluating neuroimages

Please read the following text carefully before responding.

Below you see an image that has been produced using a method called Positron-Emission-Tomography (PET). With this method, radioactive glucose is injected into the bloodstream of a person (the radiation dose is very low and not harmful for the person). The brain needs glucose. The more glucose the brain uses, the more active is the respective brain region. PET measures how much radioactive glucose has been consumed in each part of the brain (i.e.,"glucose density").

The color-scale on the right side of the image depicts the level of glucose consumption: the lowest value (at the bottom of the scale) indicates that no glucose has been consumed in the depicted part of the brain. The highest value (at the top of the scale) indicates maximal glucose consumption. Some parts of the brain (e.g., ventricles, in the center of the brain) are filled with brain liquor (basically water), and these regions naturally have a much lower glucose density.

***The question:** A researcher presents you with the image below. The researcher claims that the depicted brain shows a normal consciousness state of a person, i.e., a person without any neurological problem that may disrupt normal brain activity.*

Using the response scale below the image, please rate how much you support the researcher's claim based on the image that shows the brain activity.

If you tick the box on the far left side, you do not believe that the image supports the claim at all. If you tick the box on the far right side, you believe that the image fully supports the claim. Tick the box that you believe represents the level of support for the made claim best.

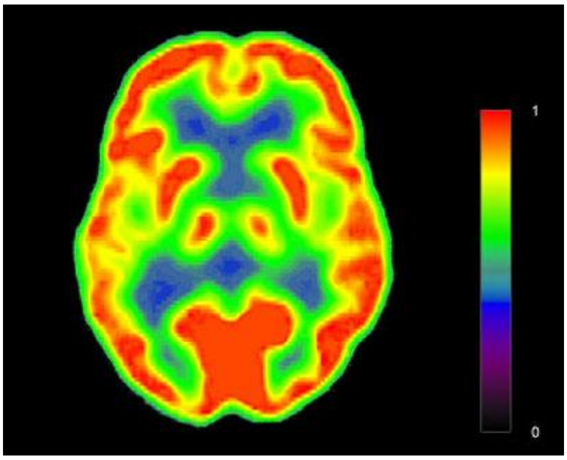

The image supports this claim not at all | ◯ ◯ ◯ ◯ ◯ ◯ ◯ ◯ | The image supports this claim very much

Evaluating neuroimages

Please read the following text carefully before responding.

The color-scale on the right side of the image depicts the level of glucose consumption: the lowest value (at the bottom of the scale) indicates that no glucose has been consumed in the depicted part of the brain. The highest value (at the top of the scale) indicates maximal glucose consumption. Some parts of the brain (e.g., ventricles, in the center of the brain) are filled with brain liquor (basically water), and these regions naturally have a much lower glucose density.

The question: *A researcher presents you with the image below. The researcher claims that the depicted brain is dead, i.e., the blood flow to the brain is blocked such that no oxygen can reach the brain. As a consequence, the brain cells cannot consume glucose any longer, and the cells are in a process of disintegration.*

Using the response scale below the image, please rate how much you support the researcher's claim based on the image that shows the brain activity.

If you tick the box on the far left side, you do not believe that the image supports the claim at all. If you tick the box on the far right side, you believe that the image fully supports the claim. Tick the box that you believe represents the level of support for the made claim best.

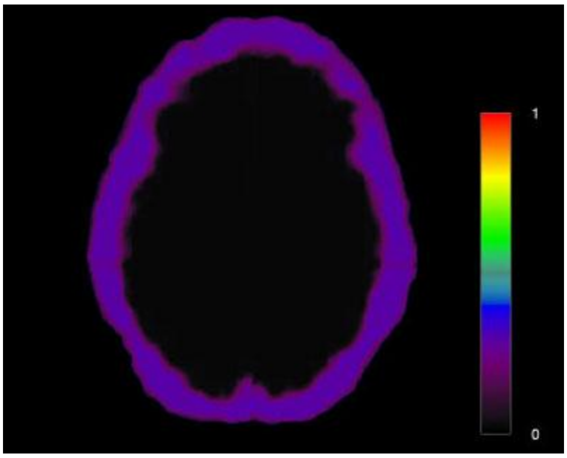

The image supports this claim not at all ○ ○ ○ ○ ○ ○ ○ ○ The image supports this claim very much

Evaluating neuroimages

Please read the following text carefully before responding.

In the beginning, you have seen two PET-images that show two extreme states with respect to brain activity: The first image depicted the brain of a normal, conscious person; the second image showed a brain of a brain dead person. We now present you with an image depicting a brain with a neurological problem.

The question: *A researcher presents you with the image below. The researcher claims that the depicted brain is in a so-called locked-in state, i.e., the brain of the person has a more or less normal consciousness state, but due to a neurological problem the person is unable to move and to communicate with the environment. This state shows reduced brain activity when measured with PET.*

Using the response scale below indicate the brain's consciousness state. The response scale shows on the far left side zero brain activity (i.e., brain death; "worst state"), and on the far right side normal, conscious brain activity ("best state").

If you tick the box on the far left side, you believe that the image shows a dead brain (no brain activity), if you tick the box on the far right side, you believe that the image shows a fully conscious, normal brain (normal brain activity). Tick the box you think best shows the brain's consciousness state.

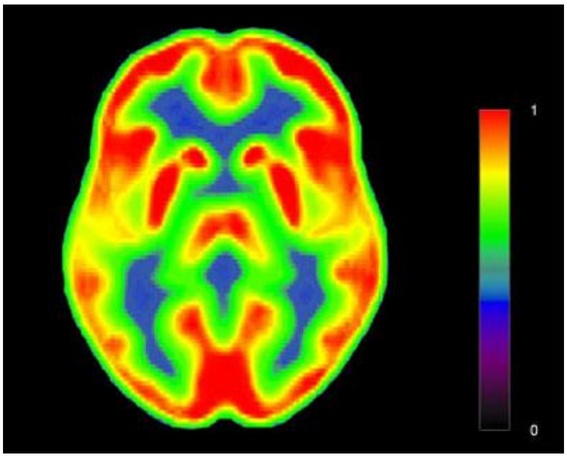

○ ○ ○ ○ ○ ○ ○ ○

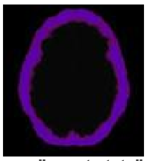

"worst state"

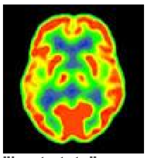

"best state"

Evaluating neuroimages

Please read the following text carefully before responding.

In the beginning, you have seen two PET-images that show two extreme states with respect to brain activity: The first image depicted the brain of a normal, conscious person; the second image showed a brain of a dead person. We now present you with an image depicting a brain with a neurological problem.

The question: *A researcher presents you with the image below. The researcher claims that the depicted brain is in a so-called minimally conscious state, i.e. the person has a severely damaged brain, but still has partial preservation of consciousness. The person also displays minimal but clear behavioral evidence of self / environmental awareness. This state shows reduced brain activity when measured with PET.*

Using the response scale below indicate the brain's consciousness state. The response scale shows on the far left side zero brain activity (i.e., brain death; "worst state"), and on the far right side normal, conscious brain activity ("best state").

If you tick the box on the far left side, you believe that the image shows a dead brain (no brain activity), if you tick the box on the far right side, you believe that the image shows a fully conscious, normal brain (normal brain activity). Tick the box you think best shows the brain's consciousness state.

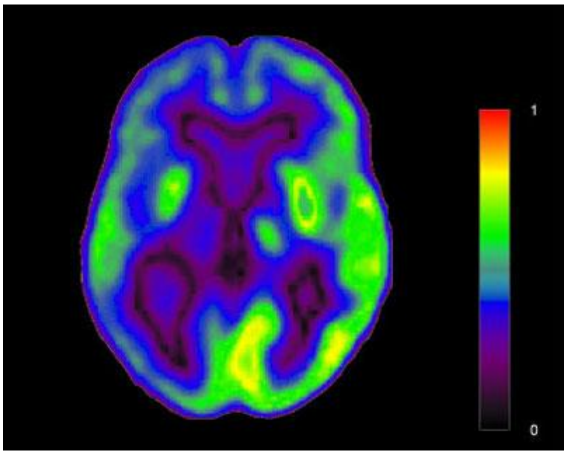

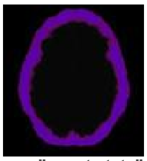

"worst state"

○ ○ ○ ○ ○ ○ ○ ○

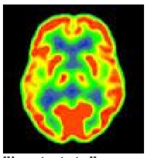

"best state"

Evaluating neuroimages

Please read the following text carefully before responding.

In the beginning, you have seen two PET-images that show two extreme states with respect to brain activity: The first image depicted the brain of a normal, conscious person; the second image showed a brain of a dead person. We now present you with an image depicting a brain with a neurological problem.

The question: *A researcher presents you with the image below. The researcher claims that the depicted brain is in a so-called vegetative state, i.e., the person has a severely damaged brain which results in a state of partial arousal rather than true awareness. The person may open the eyes occasionally, and may demonstrate sleep-wake cycles, but completely lacks cognitive functions. This state shows reduced brain activity when measured with PET.*

Using the response scale below indicate the brain's consciousness state. The response scale shows on the far left side zero brain activity (i.e., brain death; "worst state"), and on the far right side normal, conscious brain activity ("best state").

If you tick the box on the far left side, you believe that the image shows a dead brain (no brain activity), if you tick the box on the far right side, you believe that the image shows a fully conscious, normal brain (normal brain activity). Tick the box you think best shows the brain's consciousness state.

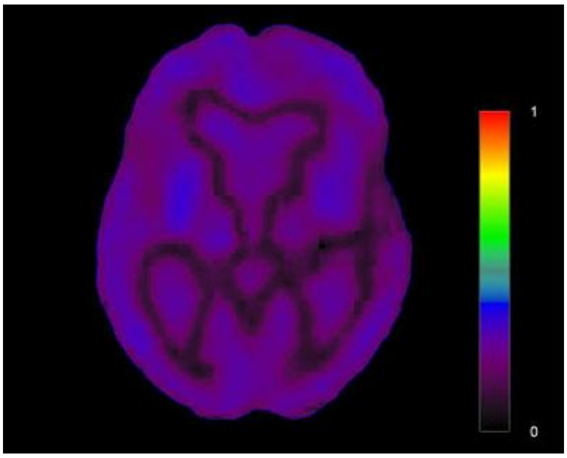

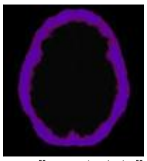

"worst state"

○ ○ ○ ○ ○ ○ ○ ○

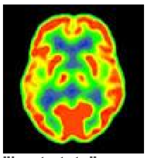

"best state"

Exp Neuro rainbow white

Evaluating neuroimages

For the next five questions, we ask you to evaluate images that have been produced using techniques to measure brain activity. But before that, we would like to know your opinion on the following matter:

"The state of brain death equals the death of a person."

Please indicate to what extent you agree with this position:

○ I completely agree; a brain dead person is dead

○ I somewhat agree; a brain dead person is probably dead

○ I somewhat disagree; a brain dead person is probably not dead

○ I completely disagree; a brain dead person is still alive

○ I have no opinion on this matter

Evaluating neuroimages

Please read the following text carefully before responding.

Below you see an image that has been produced using a method called Positron-Emission-Tomography (PET). With this method, radioactive glucose is injected into the bloodstream of a person (the radiation dose is very low and not harmful for the person). The brain needs glucose. The more glucose the brain uses, the more active is the respective brain region. PET measures how much radioactive glucose has been consumed in each part of the brain (i.e.,"glucose density").

The color-scale on the right side of the image depicts the level of glucose consumption: the lowest value (at the bottom of the scale) indicates that no glucose has been consumed in the depicted part of the brain. The highest value (at the top of the scale) indicates maximal glucose consumption. Some parts of the brain (e.g., ventricles, in the center of the brain) are filled with brain liquor (basically water), and these regions naturally have a much lower glucose density.

The question: *A researcher presents you with the image below. The researcher claims that the depicted brain shows a normal consciousness state of a person, i.e., a person without any neurological problem that may disrupt normal brain activity.*

Using the response scale below the image, please rate how much you support the researcher's claim based on the image that shows the brain activity.

If you tick the box on the far left side, you do not believe that the image supports the claim at all. If you tick the box on the far right side, you believe that the image fully supports the claim. Tick the box that you believe represents the level of support for the made claim best.

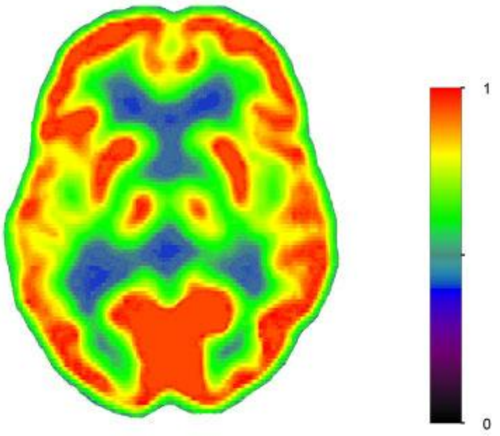

The image supports this claim not at all ◯ ◯ ◯ ◯ ◯ ◯ ◯ ◯ The image supports this claim very much

Evaluating neuroimages

Please read the following text carefully before responding.

The color-scale on the right side of the image depicts the level of glucose consumption: the lowest value (at the bottom of the scale) indicates that no glucose has been consumed in the depicted part of the brain. The highest value (at the top of the scale) indicates maximal glucose consumption. Some parts of the brain (e.g., ventricles, in the center of the brain) are filled with brain liquor (basically water), and these regions naturally have a much lower glucose density.

The question: *A researcher presents you with the image below. The researcher claims that the depicted brain is dead, i.e., the blood flow to the brain is blocked such that no oxygen can reach the brain. As a consequence, the brain cells cannot consume glucose any longer, and the cells are in a process of disintegration.*

Using the response scale below the image, please rate how much you support the researcher's claim based on the image that shows the brain activity.

If you tick the box on the far left side, you do not believe that the image supports the claim at all. If you tick the box on the far right side, you believe that the image fully supports the claim. Tick the box that you believe represents the level of support for the made claim best.

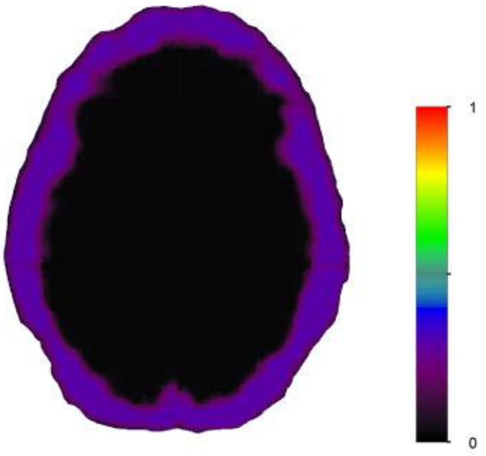

The image supports this claim not at all ◯ ◯ ◯ ◯ ◯ ◯ ◯ ◯ The image supports this claim very much

Evaluating neuroimages

Please read the following text carefully before responding.

In the beginning, you have seen two PET-images that show two extreme states with respect to brain activity: The first image depicted the brain of a normal, conscious person; the second image showed a brain of a brain dead person. We now present you with an image depicting a brain with a neurological problem.

The question: *A researcher presents you with the image below. The researcher claims that the depicted brain is in a so-called locked-in state, i.e., the brain of the person has a more or less normal consciousness state, but due to a neurological problem the person is unable to move and to communicate with the environment. This state shows reduced brain activity when measured with PET.*

Using the response scale below indicate the brain's consciousness state. The response scale shows on the far left side zero brain activity (i.e., brain death; "worst state"), and on the far right side normal, conscious brain activity ("best state").

If you tick the box on the far left side, you believe that the image shows a dead brain (no brain activity), if you tick the box on the far right side, you believe that the image shows a fully conscious, normal brain (normal brain activity). Tick the box you

think best shows the brain's consciousness state.

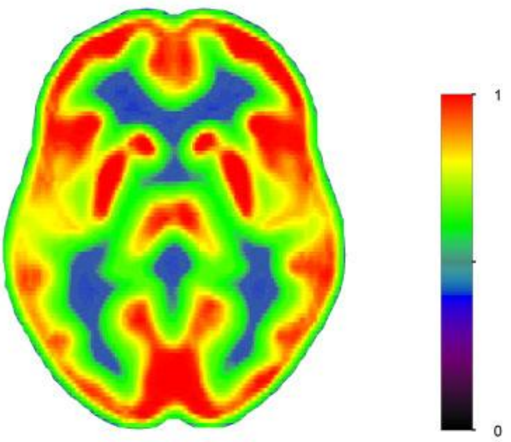

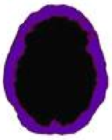

"worst state"

○ ○ ○ ○ ○ ○ ○ ○

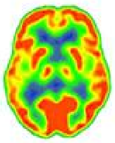

"best state"

Evaluating neuroimages

Please read the following text carefully before responding.

In the beginning, you have seen two PET-images that show two extreme states with respect to brain activity: The first image depicted the brain of a normal, conscious person; the second image showed a brain of a dead person. We now present you with an image depicting a brain with a neurological problem.

The question: *A researcher presents you with the image below. The researcher claims that the depicted brain is in a so-called minimally conscious state, i.e. the person has a severely damaged brain, but still has partial preservation of consciousness. The person also displays minimal but clear behavioral evidence of self / environmental awareness. This state shows reduced brain activity when measured with PET.*

Using the response scale below indicate the brain's consciousness state. The response scale shows on the far left side zero brain activity (i.e., brain death; "worst state"), and on the far right side normal, conscious brain activity ("best state").

If you tick the box on the far left side, you believe that the image shows a dead brain (no brain activity), if you tick the box on the far right side, you believe that the image shows a fully conscious, normal brain (normal brain activity). Tick the box you think best shows the brain's consciousness state.

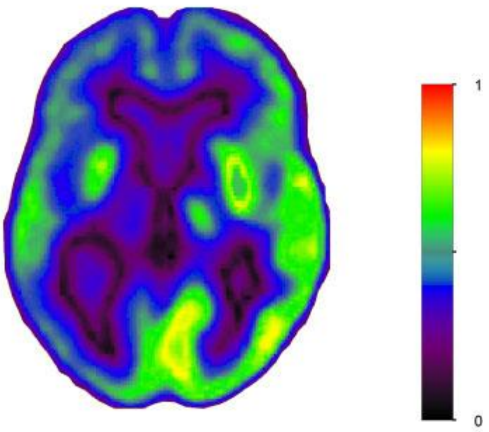

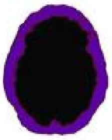

"worst state"

○ ○ ○ ○ ○ ○ ○ ○

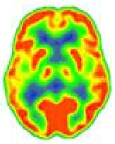

"best state"

Evaluating neuroimages

Please read the following text carefully before responding.

In the beginning, you have seen two PET-images that show two extreme states with respect to brain activity: The first image depicted the brain of a normal, conscious person; the second image showed a brain of a dead person. We now present you with an image depicting a brain with a neurological problem.

The question: *A researcher presents you with the image below. The researcher claims that the depicted brain is in a so-called vegetative state, i.e., the person has a severely damaged brain which results in a state of partial arousal rather than true awareness. The person may open the eyes occasionally, and may demonstrate sleep-wake cycles, but completely lacks cognitive functions. This state shows reduced brain activity when measured with PET.*

Using the response scale below indicate the brain's consciousness state. The response scale shows on the far left side zero brain activity (i.e., brain death; "worst state"), and on the far right side normal, conscious brain activity ("best state").

If you tick the box on the far left side, you believe that the image shows a dead brain (no brain activity), if you tick the box on the far right side, you believe that the image shows a fully conscious, normal brain (normal brain activity). Tick the box you think best shows the brain's consciousness state.

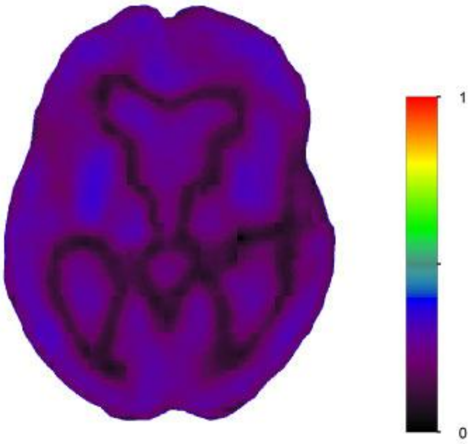

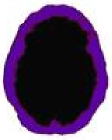

"worst state"

○ ○ ○ ○ ○ ○ ○ ○

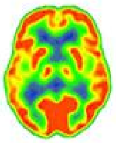

"best state"

Exp Neuro blue-red black

Evaluating neuroimages

For the next five questions, we ask you to evaluate images that have been produced using techniques to measure brain activity. But before that, we would like to know your opinion on the following matter:

"The state of brain death equals the death of a person."

Please indicate to what extent you agree with this position:

- ○ I completely agree; a brain dead person is dead
- ○ I somewhat agree; a brain dead person is probably dead
- ○ I somewhat disagree; a brain dead person is probably not dead
- ○ I completely disagree; a brain dead person is still alive
- ○ I have no opinion on this matter

Evaluating neuroimages

Please read the following text carefully before responding.

Below you see an image that has been produced using a method called Positron-Emission-Tomography (PET). With this method, radioactive glucose is injected into the bloodstream of a person (the radiation dose is very low and not harmful for the person). The brain needs glucose. The more glucose the brain uses, the more active is the respective brain region. PET measures how much radioactive glucose has been consumed in each part of the brain (i.e.,"glucose density").

The color-scale on the right side of the image depicts the level of glucose consumption: the lowest value (at the bottom of the scale) indicates that no glucose has been consumed in the depicted part of the brain. The highest value (at the top of the scale) indicates maximal glucose consumption. Some parts of the brain (e.g., ventricles, in the center of the brain) are filled with brain liquor (basically water), and these regions naturally have a much lower glucose density.

The question: *A researcher presents you with the image below. The researcher claims that the depicted brain shows a normal consciousness state of a person, i.e., a person without any neurological problem that may disrupt normal brain activity.*

Using the response scale below the image, please rate how much you support the researcher's claim based on the image that shows the brain activity.

If you tick the box on the far left side, you do not believe that the image supports the claim at all. If you tick the box on the far right side, you believe that the image fully supports the claim. Tick the box that you believe represents the level of support for the made claim best.

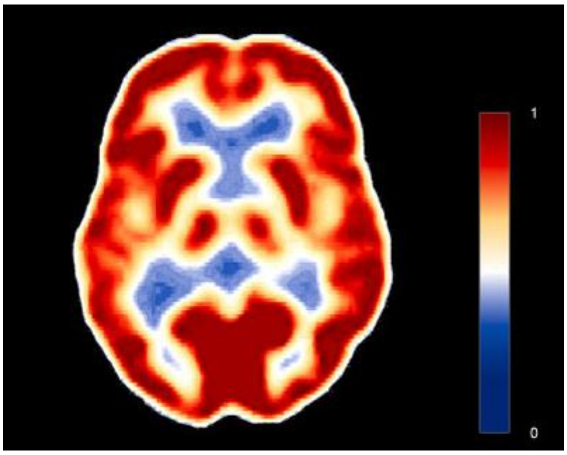

The image supports this claim not at all ○ ○ ○ ○ ○ ○ ○ ○ The image supports this claim very much

Evaluating neuroimages

Please read the following text carefully before responding.

The color-scale on the right side of the image depicts the level of glucose consumption: the lowest value (at the bottom of the scale) indicates that no glucose has been consumed in the depicted part of the brain. The highest value (at the top of the scale) indicates maximal glucose consumption. Some parts of the brain (e.g., ventricles, in the center of the brain) are filled with brain liquor (basically water), and these regions naturally have a much lower glucose density.

The question: *A researcher presents you with the image below. The researcher claims that the depicted brain is dead, i.e., the blood flow to the brain is blocked such that no oxygen can reach the brain. As a consequence, the brain cells cannot consume glucose any longer, and the cells are in a process of disintegration.*

Using the response scale below the image, please rate how much you support the researcher's claim based on the image that shows the brain activity.

If you tick the box on the far left side, you do not believe that the image supports the claim at all. If you tick the box on the far right side, you believe that the image fully supports the claim. Tick the box that you believe represents the level of support for the made claim best.

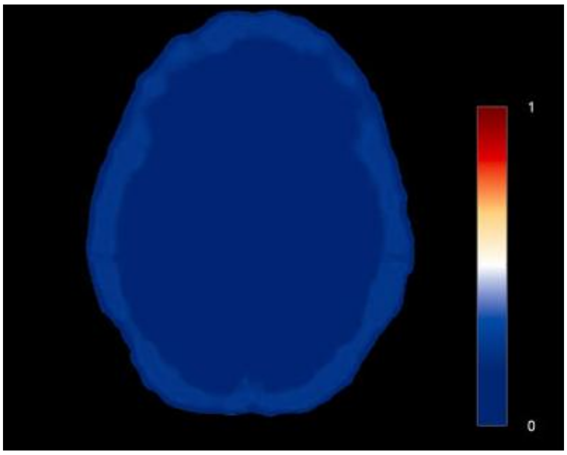

The image supports this claim not at all ○ ○ ○ ○ ○ ○ ○ ○ The image supports this claim very much

Evaluating neuroimages

Please read the following text carefully before responding.

In the beginning, you have seen two PET-images that show two extreme states with respect to brain activity: The first image depicted the brain of a normal, conscious person; the second image showed a brain of a brain dead person. We now present you with an image depicting a brain with a neurological problem.

The question: *A researcher presents you with the image below. The researcher claims that the depicted brain is in a so-called locked-in state, i.e., the brain of the person has a more or less normal consciousness state, but due to a neurological problem the person is unable to move and to communicate with the environment. This state shows reduced brain activity when measured with PET.*

Using the response scale below indicate the brain's consciousness state. The response scale shows on the far left side zero brain activity (i.e., brain death; "worst state"), and on the far right side normal, conscious brain activity ("best state").

If you tick the box on the far left side, you believe that the image shows a dead brain (no brain activity), if you tick the box on the far right side, you believe that the image shows a fully conscious, normal brain (normal brain activity). Tick the box you think best shows the brain's consciousness state.

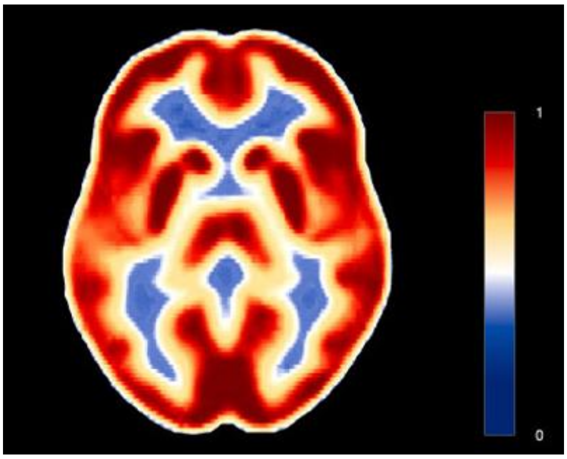

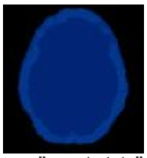

"worst state"

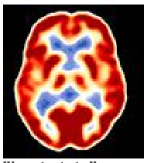

"best state"

Evaluating neuroimages

Please read the following text carefully before responding.

In the beginning, you have seen two PET-images that show two extreme states with respect to brain activity: The first image depicted the brain of a normal, conscious person; the second image showed a brain of a dead person. We now present you with an image depicting a brain with a neurological problem.

The question: *A researcher presents you with the image below. The researcher claims that the depicted brain is in a so-called minimally conscious state, i.e. the person has a severely damaged brain, but still has partial preservation of consciousness. The person also displays minimal but clear behavioral evidence of self / environmental awareness. This state shows reduced brain activity when measured with PET.*

Using the response scale below indicate the brain's consciousness state. The response scale shows on the far left side zero brain activity (i.e., brain death; "worst state"), and on the far right side normal, conscious brain activity ("best state").

If you tick the box on the far left side, you believe that the image shows a dead brain (no brain activity), if you tick the box on the far right side, you believe that the image shows a fully conscious, normal brain (normal brain activity). Tick the box you think best shows the brain's consciousness state.

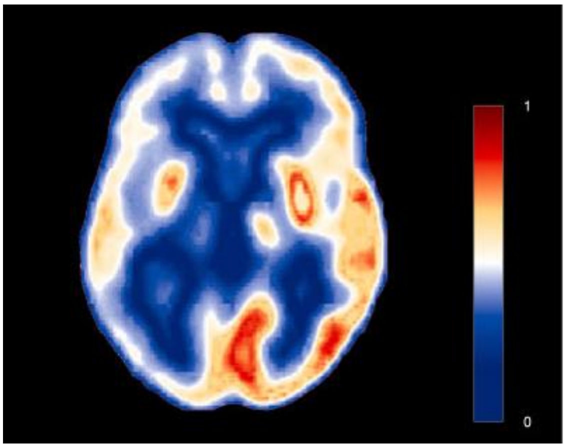

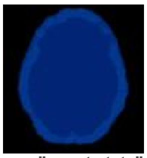

"worst state"

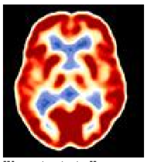

"best state"

Evaluating neuroimages

Please read the following text carefully before responding.

In the beginning, you have seen two PET-images that show two extreme states with respect to brain activity: The first image depicted the brain of a normal, conscious person; the second image showed a brain of a dead person. We now present you with an image depicting a brain with a neurological problem.

The question: *A researcher presents you with the image below. The researcher claims that the depicted brain is in a so-called vegetative state, i.e., the person has a severely damaged brain which results in a state of partial arousal rather than true awareness. The person may open the eyes occasionally, and may demonstrate sleep-wake cycles, but completely lacks cognitive functions. This state shows reduced brain activity when measured with PET.*

Using the response scale below indicate the brain's consciousness state. The response scale shows on the far left side zero brain activity (i.e., brain death; "worst state"), and on the far right side normal, conscious brain activity ("best state").

If you tick the box on the far left side, you believe that the image shows a dead brain (no brain activity), if you tick the box on the far right side, you believe that the image shows a fully conscious, normal brain (normal brain activity). Tick the box you think best shows the brain's consciousness state.

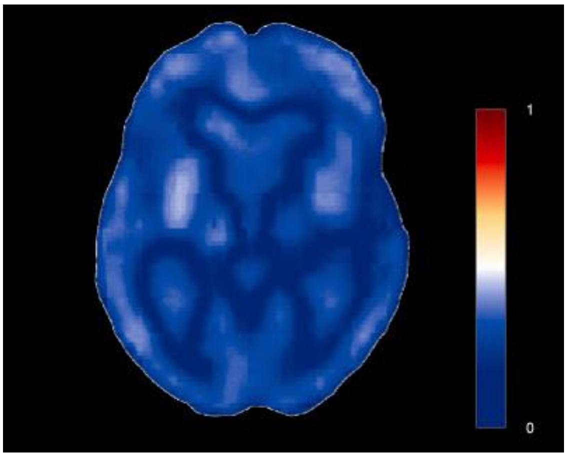

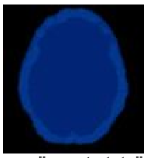

"worst state"

○ ○ ○ ○ ○ ○ ○ ○

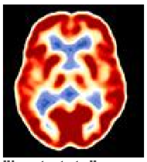

"best state"

Exp Neuro blue-red white

Evaluating neuroimages

For the next five questions, we ask you to evaluate images that have been produced using techniques to measure brain activity. But before that, we would like to know your opinion on the following matter:

"The state of brain death equals the death of a person."

Please indicate to what extent you agree with this position:

○ I completely agree; a brain dead person is dead

○ I somewhat agree; a brain dead person is probably dead

○ I somewhat disagree; a brain dead person is probably not dead

○ I completely disagree; a brain dead person is still alive

○ I have no opinion on this matter

Evaluating neuroimages

Please read the following text carefully before responding.

Below you see an image that has been produced using a method called Positron-Emission-Tomography (PET). With this method, radioactive glucose is injected into the bloodstream of a person (the radiation dose is very low and not harmful for the person). The brain needs glucose. The more glucose the brain uses, the more active is the respective brain region. PET measures how much radioactive glucose has been consumed in each part of the brain (i.e.,"glucose density").

The color-scale on the right side of the image depicts the level of glucose consumption: the lowest value (at the bottom of the scale) indicates that no glucose has been consumed in the depicted part of the brain. The highest value (at the top of the scale) indicates maximal glucose consumption. Some parts of the brain (e.g., ventricles, in the center of the brain) are filled with brain liquor (basically water), and these regions naturally have a much lower glucose density.

The question: *A researcher presents you with the image below. The researcher claims that the depicted brain shows a normal consciousness state of a person, i.e., a person without any neurological problem that may disrupt normal brain activity.*

Using the response scale below the image, please rate how much you support the researcher's claim based on the image that shows the brain activity.

If you tick the box on the far left side, you do not believe that the image supports the claim at all. If you tick the box on the far right side, you believe that the image fully supports the claim. Tick the box that you believe represents the level of support for the made claim best.

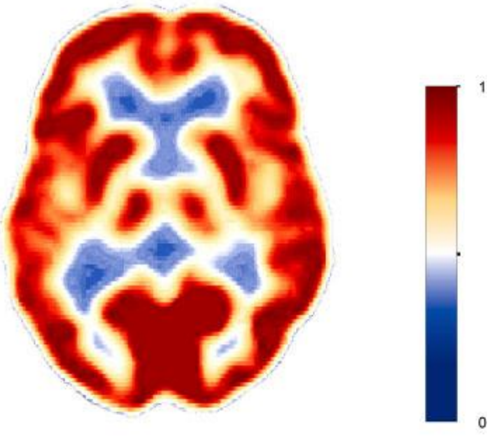

The image supports this claim not at all ○ ○ ○ ○ ○ ○ ○ ○ The image supports this claim very much

Evaluating neuroimages

Please read the following text carefully before responding.

The color-scale on the right side of the image depicts the level of glucose consumption: the lowest value (at the bottom of the scale) indicates that no glucose has been consumed in the depicted part of the brain. The highest value (at the top of the scale) indicates maximal glucose consumption. Some parts of the brain (e.g., ventricles, in the center of the brain) are filled with brain liquor (basically water), and these regions naturally have a much lower glucose density.

The question: *A researcher presents you with the image below. The researcher claims that the depicted brain is dead, i.e., the blood flow to the brain is blocked such that no oxygen can reach the brain. As a consequence, the brain cells cannot consume glucose any longer, and the cells are in a process of disintegration.*

Using the response scale below the image, please rate how much you support the researcher's claim based on the image that shows the brain activity.

If you tick the box on the far left side, you do not believe that the image supports the claim at all. If you tick the box on the far right side, you believe that the image fully supports the claim. Tick the box that you believe represents the level of support for the made claim best.

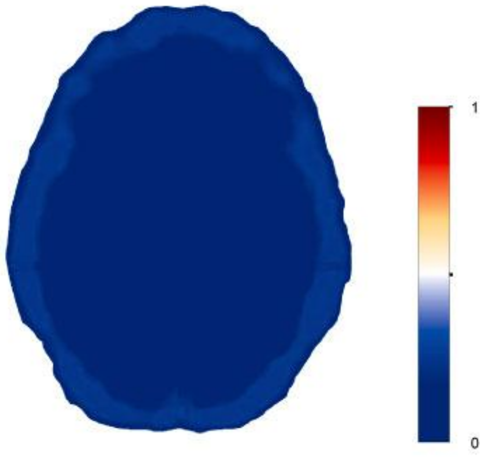

The image supports this claim not at all ○ ○ ○ ○ ○ ○ ○ ○ The image supports this claim very much

Evaluating neuroimages

Please read the following text carefully before responding.

In the beginning, you have seen two PET-images that show two extreme states with respect to brain activity: The first image depicted the brain of a normal, conscious person; the second image showed a brain of a brain dead person. We now present you with an image depicting a brain with a neurological problem.

The question: *A researcher presents you with the image below. The researcher claims that the depicted brain is in a so-called locked-in state, i.e., the brain of the person has a more or less normal consciousness state, but due to a neurological problem the person is unable to move and to communicate with the environment. This state shows reduced brain activity*

when measured with PET.

Using the response scale below indicate the brain's consciousness state. The response scale shows on the far left side zero brain activity (i.e., brain death; "worst state"), and on the far right side normal, conscious brain activity ("best state").

If you tick the box on the far left side, you believe that the image shows a dead brain (no brain activity), if you tick the box on the far right side, you believe that the image shows a fully conscious, normal brain (normal brain activity). Tick the box you think best shows the brain's consciousness state.

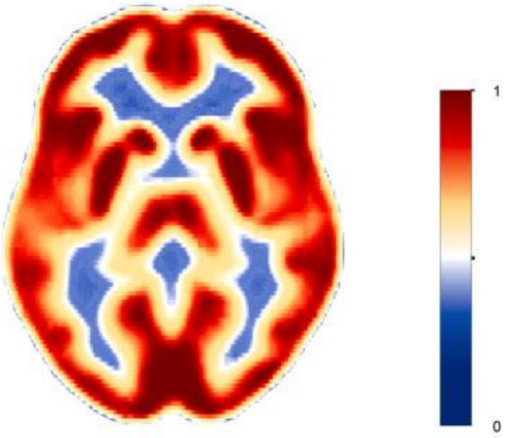

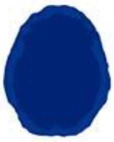

"worst state"

○ ○ ○ ○ ○ ○ ○ ○

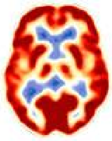

"best state"

Evaluating neuroimages

Please read the following text carefully before responding.

In the beginning, you have seen two PET-images that show two extreme states with respect to brain activity: The first image depicted the brain of a normal, conscious person; the second image showed a brain of a dead person. We now present you with an image depicting a brain with a neurological problem.

The question: *A researcher presents you with the image below. The researcher claims that the depicted brain is in a so-called minimally conscious state, i.e. the person has a severely damaged brain, but still has partial preservation of consciousness. The person also displays minimal but clear behavioral evidence of self / environmental awareness. This state shows reduced brain activity when measured with PET.*

Using the response scale below indicate the brain's consciousness state. The response scale shows on the far left side zero brain activity (i.e., brain death; "worst state"), and on the far right side normal, conscious brain activity ("best state").

If you tick the box on the far left side, you believe that the image shows a dead brain (no brain activity), if you tick the box on the far right side, you believe that the image shows a fully conscious, normal brain (normal brain activity). Tick the box you think best shows the brain's consciousness state.

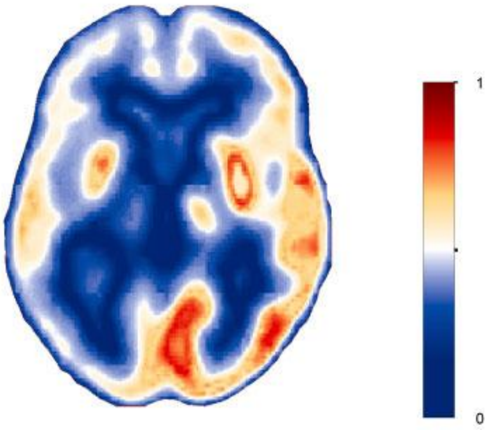

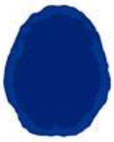

○ ○ ○ ○ ○ ○ ○ ○

"worst state"

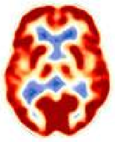

"best state"

Evaluating neuroimages

Please read the following text carefully before responding.

In the beginning, you have seen two PET-images that show two extreme states with respect to brain activity: The first image depicted the brain of a normal, conscious person; the second image showed a brain of a dead person. We now present you with an image depicting a brain with a neurological problem.

The question: *A researcher presents you with the image below. The researcher claims that the depicted brain is in a so-called vegetative state, i.e., the person has a severely damaged brain which results in a state of partial arousal rather than true awareness. The person may open the eyes occasionally, and may demonstrate sleep-wake cycles, but completely lacks cognitive functions. This state shows reduced brain activity when measured with PET.*

Using the response scale below indicate the brain's consciousness state. The response scale shows on the far left side zero brain activity (i.e., brain death; "worst state"), and on the far right side normal, conscious brain activity ("best state").

If you tick the box on the far left side, you believe that the image shows a dead brain (no brain activity), if you tick the box on the far right side, you believe that the image shows a fully conscious, normal brain (normal brain activity). Tick the box you think best shows the brain's consciousness state.

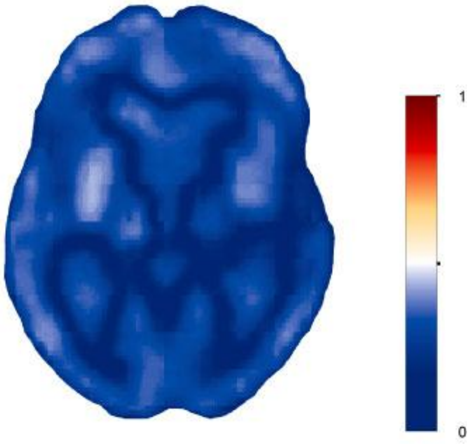

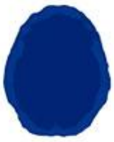

"worst state"

○ ○ ○ ○ ○ ○ ○ ○

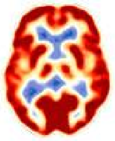

"best state"

Exp Neuro green black

Evaluating neuroimages

For the next five questions, we ask you to evaluate images that have been produced using techniques to measure brain activity. But before that, we would like to know your opinion on the following matter:

"The state of brain death equals the death of a person."

Please indicate to what extent you agree with this position:

- ○ I completely agree; a brain dead person is dead
- ○ I somewhat agree; a brain dead person is probably dead
- ○ I somewhat disagree; a brain dead person is probably not dead
- ○ I completely disagree; a brain dead person is still alive
- ○ I have no opinion on this matter

Evaluating neuroimages

Please read the following text carefully before responding.

Below you see an image that has been produced using a method called Positron-Emission-Tomography (PET). With this method, radioactive glucose is injected into the bloodstream of a person (the radiation dose is very low and not harmful for the person). The brain needs glucose. The more glucose the brain uses, the more active is the respective brain region. PET measures how much radioactive glucose has been consumed in each part of the brain (i.e.,"glucose density").

The color-scale on the right side of the image depicts the level of glucose consumption: the lowest value (at the bottom of the scale) indicates that no glucose has been consumed in the depicted part of the brain. The highest value (at the top of the scale) indicates maximal glucose consumption. Some parts of the brain (e.g., ventricles, in the center of the brain) are filled with brain liquor (basically water), and these regions naturally have a much lower glucose density.

The question: *A researcher presents you with the image below. The researcher claims that the depicted brain shows a normal consciousness state of a person, i.e., a person without any neurological problem that may disrupt normal brain activity.*

Using the response scale below the image, please rate how much you support the researcher's claim based on the image that shows the brain activity.

If you tick the box on the far left side, you do not believe that the image supports the claim at all. If you tick the box on the far right side, you believe that the image fully supports the claim. Tick the box that you believe represents the level of support for the made claim best.

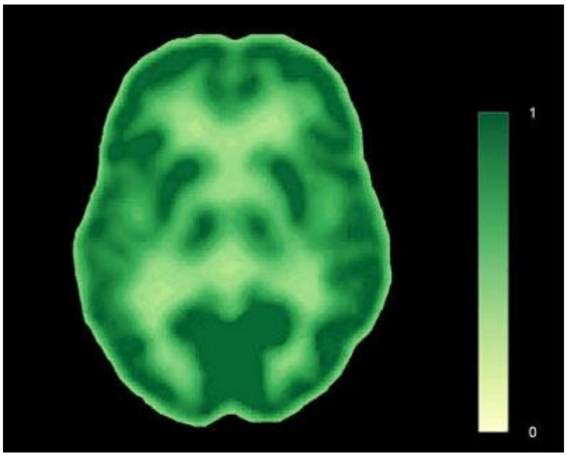

The image supports this claim not at all ○ ○ ○ ○ ○ ○ ○ ○ The image supports this claim very much

Evaluating neuroimages

Please read the following text carefully before responding.

The color-scale on the right side of the image depicts the level of glucose consumption: the lowest value (at the bottom of the scale) indicates that no glucose has been consumed in the depicted part of the brain. The highest value (at the top of the scale) indicates maximal glucose consumption. Some parts of the brain (e.g., ventricles, in the center of the brain) are filled with brain liquor (basically water), and these regions naturally have a much lower glucose density.

The question: *A researcher presents you with the image below. The researcher claims that the depicted brain is dead, i.e., the blood flow to the brain is blocked such that no oxygen can reach the brain. As a consequence, the brain cells cannot consume glucose any longer, and the cells are in a process of disintegration.*

Using the response scale below the image, please rate how much you support the researcher's claim based on the image that shows the brain activity.

If you tick the box on the far left side, you do not believe that the image supports the claim at all. If you tick the box on the far right side, you believe that the image fully supports the claim. Tick the box that you believe represents the level of support for the made claim best.

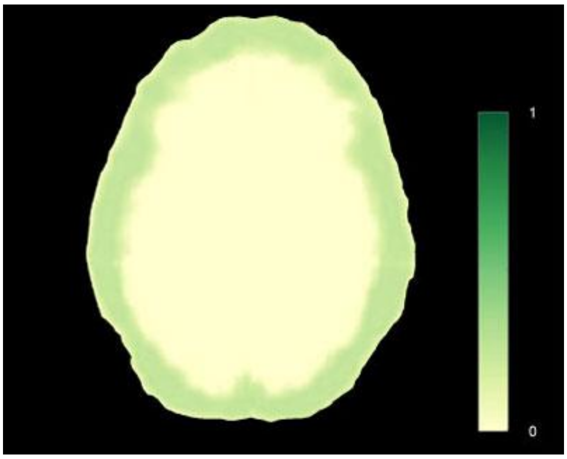

The image supports this claim not at all ○ ○ ○ ○ ○ ○ ○ ○ The image supports this claim very much

Evaluating neuroimages

Please read the following text carefully before responding.

In the beginning, you have seen two PET-images that show two extreme states with respect to brain activity: The first image depicted the brain of a normal, conscious person; the second image showed a brain of a brain dead person. We now present you with an image depicting a brain with a neurological problem.

The question: *A researcher presents you with the image below. The researcher claims that the depicted brain is in a so-called locked-in state, i.e., the brain of the person has a more or less normal consciousness state, but due to a neurological problem the person is unable to move and to communicate with the environment. This state shows reduced brain activity when measured with PET.*

Using the response scale below indicate the brain's consciousness state. The response scale shows on the far left side zero brain activity (i.e., brain death; "worst state"), and on the far right side normal, conscious brain activity ("best state").

If you tick the box on the far left side, you believe that the image shows a dead brain (no brain activity), if you tick the box on the far right side, you believe that the image shows a fully conscious, normal brain (normal brain activity). Tick the box you think best shows the brain's consciousness state.

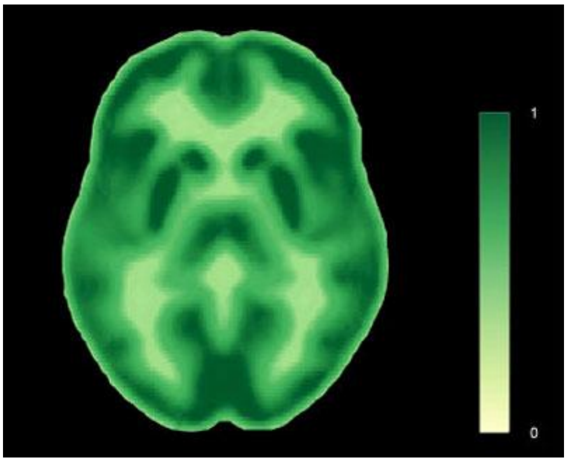

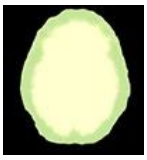

"worst state"

○ ○ ○ ○ ○ ○ ○ ○

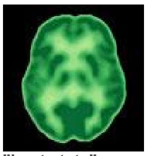

"best state"

Evaluating neuroimages

Please read the following text carefully before responding.

In the beginning, you have seen two PET-images that show two extreme states with respect to brain activity: The first image depicted the brain of a normal, conscious person; the second image showed a brain of a dead person. We now present you with an image depicting a brain with a neurological problem.

The question: *A researcher presents you with the image below. The researcher claims that the depicted brain is in a so-called minimally conscious state, i.e. the person has a severely damaged brain, but still has partial preservation of consciousness. The person also displays minimal but clear behavioral evidence of self / environmental awareness. This state shows reduced brain activity when measured with PET.*

Using the response scale below indicate the brain's consciousness state. The response scale shows on the far left side zero brain activity (i.e., brain death; "worst state"), and on the far right side normal, conscious brain activity ("best state").

If you tick the box on the far left side, you believe that the image shows a dead brain (no brain activity), if you tick the box on the far right side, you believe that the image shows a fully conscious, normal brain (normal brain activity). Tick the box you think best shows the brain's consciousness state.

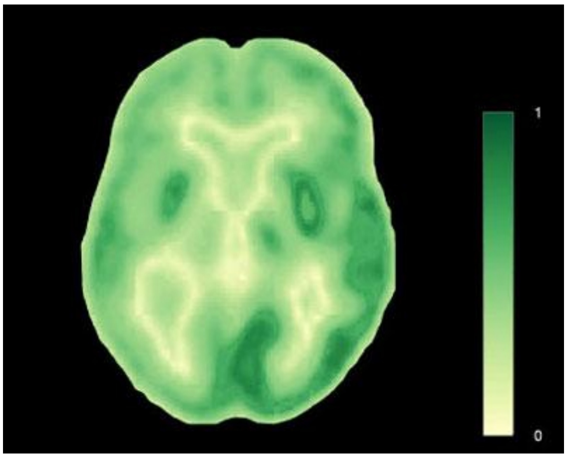

○ ○ ○ ○ ○ ○ ○ ○

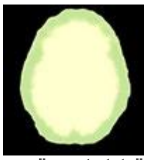

"worst state"

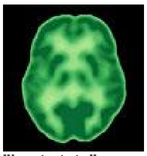

"best state"

Evaluating neuroimages

Please read the following text carefully before responding.

In the beginning, you have seen two PET-images that show two extreme states with respect to brain activity: The first image depicted the brain of a normal, conscious person; the second image showed a brain of a dead person. We now present you with an image depicting a brain with a neurological problem.

The question: *A researcher presents you with the image below. The researcher claims that the depicted brain is in a so-called vegetative state, i.e., the person has a severely damaged brain which results in a state of partial arousal rather than true awareness. The person may open the eyes occasionally, and may demonstrate sleep-wake cycles, but completely lacks cognitive functions. This state shows reduced brain activity when measured with PET.*

Using the response scale below indicate the brain’s consciousness state. The response scale shows on the far left side zero brain activity (i.e., brain death; "worst state"), and on the far right side normal, conscious brain activity ("best state").

If you tick the box on the far left side, you believe that the image shows a dead brain (no brain activity), if you tick the box on the far right side, you believe that the image shows a fully conscious, normal brain (normal brain activity). Tick the box you think best shows the brain’s consciousness state.

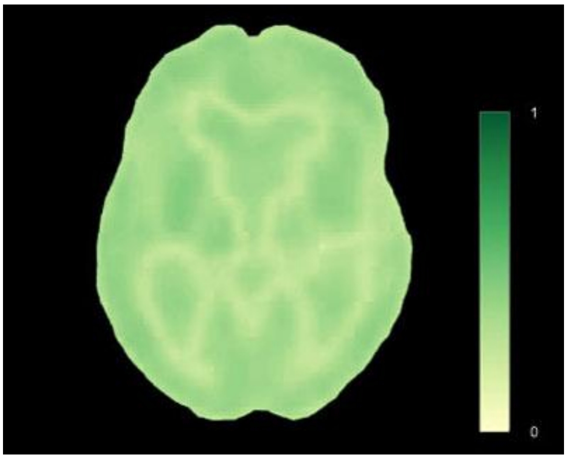

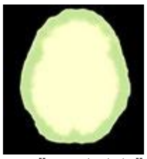

"worst state"

○ ○ ○ ○ ○ ○ ○ ○

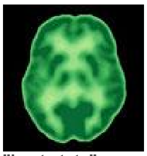

"best state"

Exp Neuro green white

Evaluating neuroimages

For the next five questions, we ask you to evaluate images that have been produced using techniques to measure brain activity. But before that, we would like to know your opinion on the following matter:

“The state of brain death equals the death of a person.”

Please indicate to what extent you agree with this position:

- ○ I completely agree; a brain dead person is dead
- ○ I somewhat agree; a brain dead person is probably dead
- ○ I somewhat disagree; a brain dead person is probably not dead
- ○ I completely disagree; a brain dead person is still alive
- ○ I have no opinion on this matter

Evaluating neuroimages

Please read the following text carefully before responding.

Below you see an image that has been produced using a method called Positron-Emission-Tomography (PET). With this method, radioactive glucose is injected into the bloodstream of a person (the radiation dose is very low and not harmful for the person). The brain needs glucose. The more glucose the brain uses, the more active is the respective brain region. PET

measures how much radioactive glucose has been consumed in each part of the brain (i.e.,“glucose density”).

The color-scale on the right side of the image depicts the level of glucose consumption: the lowest value (at the bottom of the scale) indicates that no glucose has been consumed in the depicted part of the brain. The highest value (at the top of the scale) indicates maximal glucose consumption. Some parts of the brain (e.g., ventricles, in the center of the brain) are filled with brain liquor (basically water), and these regions naturally have a much lower glucose density.

The question: *A researcher presents you with the image below. The researcher claims that the depicted brain shows a normal consciousness state of a person, i.e., a person without any neurological problem that may disrupt normal brain activity.*

Using the response scale below the image, please rate how much you support the researcher’s claim based on the image that shows the brain activity.

If you tick the box on the far left side, you do not believe that the image supports the claim at all. If you tick the box on the far right side, you believe that the image fully supports the claim. Tick the box that you believe represents the level of support for the made claim best.

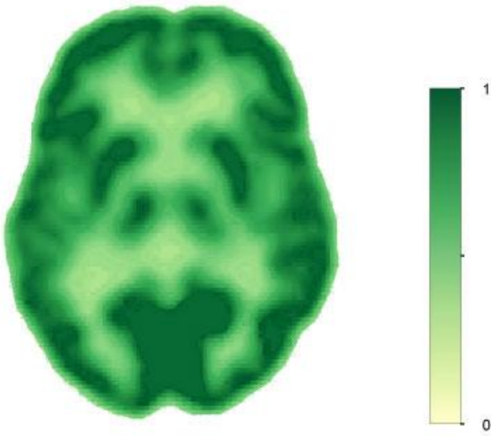

The image supports this claim not at all ○ ○ ○ ○ ○ ○ ○ ○ The image supports this claim very much

Evaluating neuroimages

Please read the following text carefully before responding.

The color-scale on the right side of the image depicts the level of glucose consumption: the lowest value (at the bottom of the scale) indicates that no glucose has been consumed in the depicted part of the brain. The highest value (at the top of the scale) indicates maximal glucose consumption. Some parts of the brain (e.g., ventricles, in the center of the brain) are filled with brain liquor (basically water), and these regions naturally have a much lower glucose density.

The question: *A researcher presents you with the image below. The researcher claims that the depicted brain is dead, i.e., the blood flow to the brain is blocked such that no oxygen can reach the brain. As a consequence, the brain cells cannot consume glucose any longer, and the cells are in a process of disintegration.*

Using the response scale below the image, please rate how much you support the researcher’s claim based on the image that shows the brain activity.

If you tick the box on the far left side, you do not believe that the image supports the claim at all. If you tick the box on the far right side, you believe that the image fully supports the claim. Tick the box that you believe represents the level of support for the made claim best.

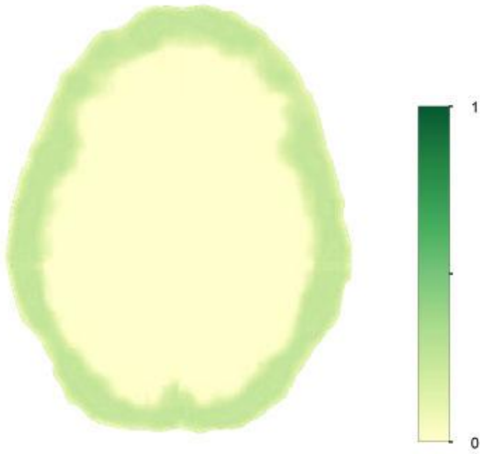

The image supports this claim not at all ○ ○ ○ ○ ○ ○ ○ ○ The image supports this claim very much

Evaluating neuroimages

Please read the following text carefully before responding.

In the beginning, you have seen two PET-images that show two extreme states with respect to brain activity: The first image depicted the brain of a normal, conscious person; the second image showed a brain of a brain dead person. We now present you with an image depicting a brain with a neurological problem.

The question: *A researcher presents you with the image below. The researcher claims that the depicted brain is in a so-called locked-in state, i.e., the brain of the person has a more or less normal consciousness state, but due to a neurological problem the person is unable to move and to communicate with the environment. This state shows reduced brain activity when measured with PET.*

Using the response scale below indicate the brain's consciousness state. The response scale shows on the far left side zero brain activity (i.e., brain death; "worst state"), and on the far right side normal, conscious brain activity ("best state").

If you tick the box on the far left side, you believe that the image shows a dead brain (no brain activity), if you tick the box on the far right side, you believe that the image shows a fully conscious, normal brain (normal brain activity). Tick the box you think best shows the brain's consciousness state.

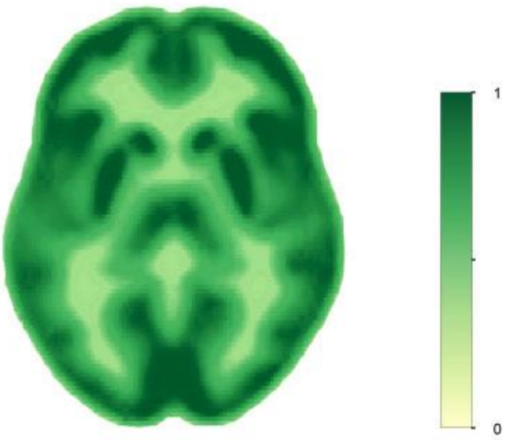

"worst state"

○ ○ ○ ○ ○ ○ ○ ○

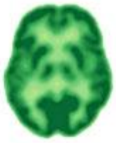

"best state"

Evaluating neuroimages

Please read the following text carefully before responding.

In the beginning, you have seen two PET-images that show two extreme states with respect to brain activity: The first image depicted the brain of a normal, conscious person; the second image showed a brain of a dead person. We now present you with an image depicting a brain with a neurological problem.

The question: *A researcher presents you with the image below. The researcher claims that the depicted brain is in a so-called minimally conscious state, i.e. the person has a severely damaged brain, but still has partial preservation of consciousness. The person also displays minimal but clear behavioral evidence of self / environmental awareness. This state shows reduced brain activity when measured with PET.*

Using the response scale below indicate the brain's consciousness state. The response scale shows on the far left side zero brain activity (i.e., brain death; "worst state"), and on the far right side normal, conscious brain activity ("best state").

If you tick the box on the far left side, you believe that the image shows a dead brain (no brain activity), if you tick the box on the far right side, you believe that the image shows a fully conscious, normal brain (normal brain activity). Tick the box you think best shows the brain's consciousness state.

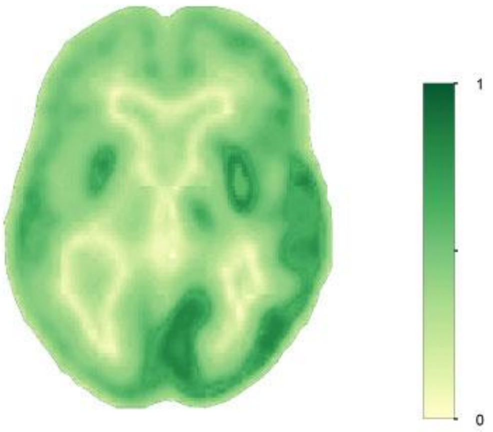

"worst state"

○ ○ ○ ○ ○ ○ ○ ○

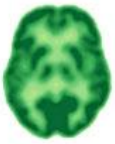

"best state"

Evaluating neuroimages

Please read the following text carefully before responding.

In the beginning, you have seen two PET-images that show two extreme states with respect to brain activity: The first image depicted the brain of a normal, conscious person; the second image showed a brain of a dead person. We now present you with an image depicting a brain with a neurological problem.

The question: *A researcher presents you with the image below. The researcher claims that the depicted brain is in a so-called vegetative state, i.e., the person has a severely damaged brain which results in a state of partial arousal rather than true awareness. The person may open the eyes occasionally, and may demonstrate sleep-wake cycles, but completely lacks cognitive functions. This state shows reduced brain activity when measured with PET.*

Using the response scale below indicate the brain's consciousness state. The response scale shows on the far left side zero brain activity (i.e., brain death; "worst state"), and on the far right side normal, conscious brain activity ("best state").

If you tick the box on the far left side, you believe that the image shows a dead brain (no brain activity), if you tick the box on the far right side, you believe that the image shows a fully conscious, normal brain (normal brain activity). Tick the box you think best shows the brain's consciousness state.

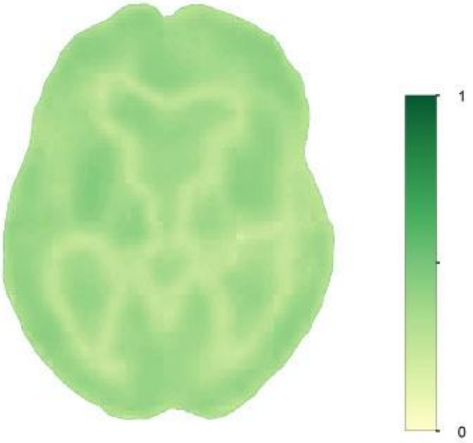

"worst state"

○ ○ ○ ○ ○ ○ ○ ○

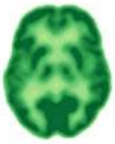

"best state"

Exp Neuro red-yellow black

Evaluating neuroimages

For the next five questions, we ask you to evaluate images that have been produced using techniques to measure brain activity. But before that, we would like to know your opinion on the following matter:

"The state of brain death equals the death of a person."

Please indicate to what extent you agree with this position:

- ◯ I completely agree; a brain dead person is dead
- ◯ I somewhat agree; a brain dead person is probably dead
- ◯ I somewhat disagree; a brain dead person is probably not dead
- ◯ I completely disagree; a brain dead person is still alive
- ◯ I have no opinion on this matter

Evaluating neuroimages

Please read the following text carefully before responding.

Below you see an image that has been produced using a method called Positron-Emission-Tomography (PET). With this method, radioactive glucose is injected into the bloodstream of a person (the radiation dose is very low and not harmful for the person). The brain needs glucose. The more glucose the brain uses, the more active is the respective brain region. PET measures how much radioactive glucose has been consumed in each part of the brain (i.e.,"glucose density").

The color-scale on the right side of the image depicts the level of glucose consumption: the lowest value (at the bottom of the scale) indicates that no glucose has been consumed in the depicted part of the brain. The highest value (at the top of the scale) indicates maximal glucose consumption. Some parts of the brain (e.g., ventricles, in the center of the brain) are filled with brain liquor (basically water), and these regions naturally have a much lower glucose density.

The question: *A researcher presents you with the image below. The researcher claims that the depicted brain shows a normal consciousness state of a person, i.e., a person without any neurological problem that may disrupt normal brain activity.*

Using the response scale below the image, please rate how much you support the researcher's claim based on the image that shows the brain activity.

If you tick the box on the far left side, you do not believe that the image supports the claim at all. If you tick the box on the far right side, you believe that the image fully supports the claim. Tick the box that you believe represents the level of support for the made claim best.

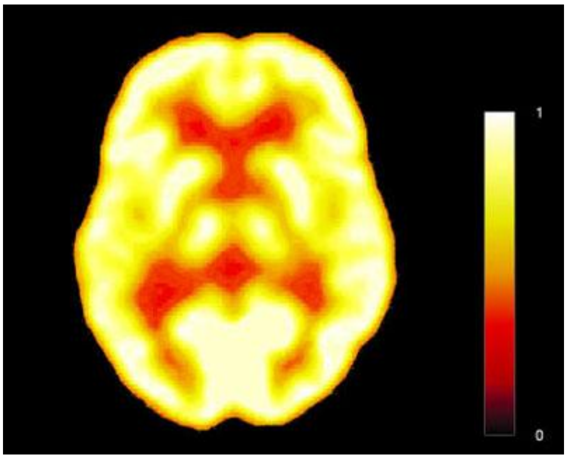

The image supports this claim not at all | ◯ ◯ ◯ ◯ ◯ ◯ ◯ ◯ | The image supports this claim very much

Evaluating neuroimages

Please read the following text carefully before responding.

The color-scale on the right side of the image depicts the level of glucose consumption: the lowest value (at the bottom of the scale) indicates that no glucose has been consumed in the depicted part of the brain. The highest value (at the top of the scale) indicates maximal glucose consumption. Some parts of the brain (e.g., ventricles, in the center of the brain) are filled with brain liquor (basically water), and these regions naturally have a much lower glucose density.

The question: *A researcher presents you with the image below. The researcher claims that the depicted brain is dead, i.e., the blood flow to the brain is blocked such that no oxygen can reach the brain. As a consequence, the brain cells cannot consume glucose any longer, and the cells are in a process of disintegration.*

Using the response scale below the image, please rate how much you support the researcher's claim based on the image that shows the brain activity.

If you tick the box on the far left side, you do not believe that the image supports the claim at all. If you tick the box on the far right side, you believe that the image fully supports the claim. Tick the box that you believe represents the level of support for the made claim best.

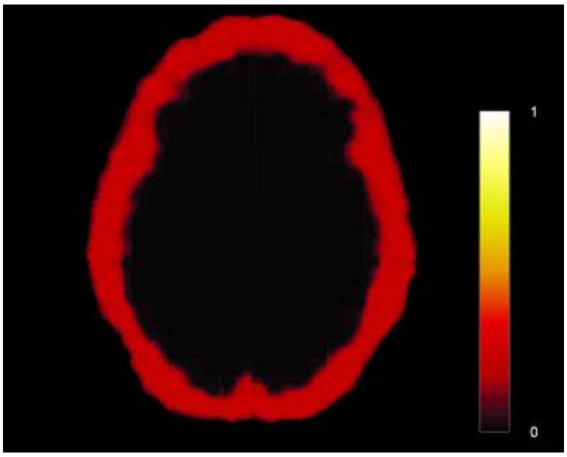

The image supports this claim not at all ○ ○ ○ ○ ○ ○ ○ ○ The image supports this claim very much

Evaluating neuroimages

Please read the following text carefully before responding.

In the beginning, you have seen two PET-images that show two extreme states with respect to brain activity: The first image depicted the brain of a normal, conscious person; the second image showed a brain of a brain dead person. We now present you with an image depicting a brain with a neurological problem.

The question: *A researcher presents you with the image below. The researcher claims that the depicted brain is in a so-called locked-in state, i.e., the brain of the person has a more or less normal consciousness state, but due to a neurological problem the person is unable to move and to communicate with the environment. This state shows reduced brain activity when measured with PET.*

Using the response scale below indicate the brain's consciousness state. The response scale shows on the far left side zero brain activity (i.e., brain death; "worst state"), and on the far right side normal, conscious brain activity ("best state").

If you tick the box on the far left side, you believe that the image shows a dead brain (no brain activity), if you tick the box on the far right side, you believe that the image shows a fully conscious, normal brain (normal brain activity). Tick the box you think best shows the brain's consciousness state.

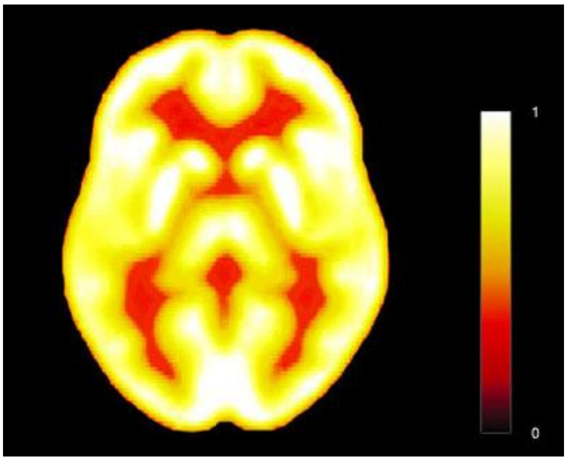

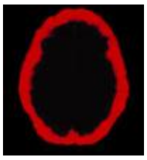

"worst state"

○ ○ ○ ○ ○ ○ ○ ○

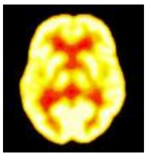

"best state"

Evaluating neuroimages

Please read the following text carefully before responding.

In the beginning, you have seen two PET-images that show two extreme states with respect to brain activity: The first image depicted the brain of a normal, conscious person; the second image showed a brain of a dead person. We now present you with an image depicting a brain with a neurological problem.

The question: *A researcher presents you with the image below. The researcher claims that the depicted brain is in a so-called minimally conscious state, i.e. the person has a severely damaged brain, but still has partial preservation of consciousness. The person also displays minimal but clear behavioral evidence of self / environmental awareness. This state shows reduced brain activity when measured with PET.*

Using the response scale below indicate the brain's consciousness state. The response scale shows on the far left side zero brain activity (i.e., brain death; "worst state"), and on the far right side normal, conscious brain activity ("best state").

If you tick the box on the far left side, you believe that the image shows a dead brain (no brain activity), if you tick the box on the far right side, you believe that the image shows a fully conscious, normal brain (normal brain activity). Tick the box you think best shows the brain's consciousness state.

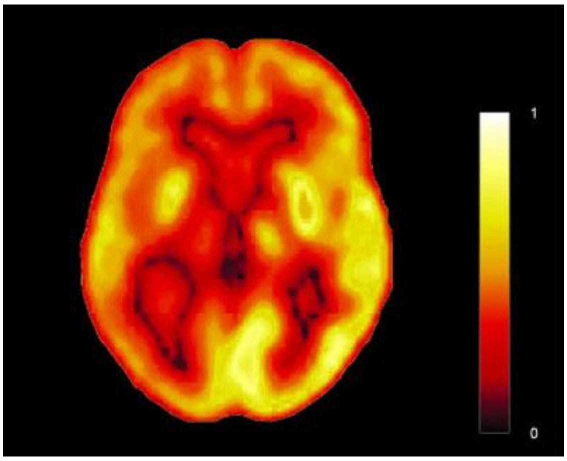

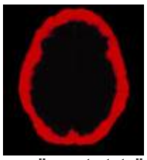

"worst state"

○ ○ ○ ○ ○ ○ ○ ○

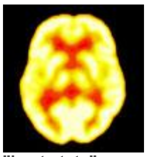

"best state"

Evaluating neuroimages

Please read the following text carefully before responding.

In the beginning, you have seen two PET-images that show two extreme states with respect to brain activity: The first image depicted the brain of a normal, conscious person; the second image showed a brain of a dead person. We now present you with an image depicting a brain with a neurological problem.

The question: *A researcher presents you with the image below. The researcher claims that the depicted brain is in a so-called vegetative state, i.e., the person has a severely damaged brain which results in a state of partial arousal rather than true awareness. The person may open the eyes occasionally, and may demonstrate sleep-wake cycles, but completely lacks cognitive functions. This state shows reduced brain activity when measured with PET.*

Using the response scale below indicate the brain's consciousness state. The response scale shows on the far left side zero brain activity (i.e., brain death; "worst state"), and on the far right side normal, conscious brain activity ("best state").

If you tick the box on the far left side, you believe that the image shows a dead brain (no brain activity), if you tick the box on the far right side, you believe that the image shows a fully conscious, normal brain (normal brain activity). Tick the box you think best shows the brain's consciousness state.

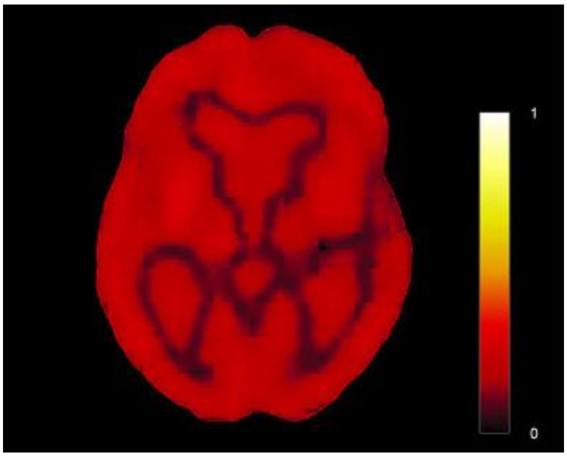

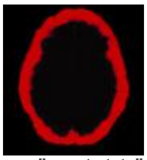

"worst state"

○ ○ ○ ○ ○ ○ ○ ○

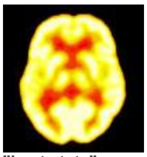

"best state"

Exp Neuro red-yellow white

Evaluating neuroimages

For the next five questions, we ask you to evaluate images that have been produced using techniques to measure brain activity. But before that, we would like to know your opinion on the following matter:

"The state of brain death equals the death of a person."

Please indicate to what extent you agree with this position:

- ◯ I completely agree; a brain dead person is dead
- ◯ I somewhat agree; a brain dead person is probably dead
- ◯ I somewhat disagree; a brain dead person is probably not dead
- ◯ I completely disagree; a brain dead person is still alive
- ◯ I have no opinion on this matter

Evaluating neuroimages

Please read the following text carefully before responding.

Below you see an image that has been produced using a method called Positron-Emission-Tomography (PET). With this method, radioactive glucose is injected into the bloodstream of a person (the radiation dose is very low and not harmful for the person). The brain needs glucose. The more glucose the brain uses, the more active is the respective brain region. PET measures how much radioactive glucose has been consumed in each part of the brain (i.e.,"glucose density").

The color-scale on the right side of the image depicts the level of glucose consumption: the lowest value (at the bottom of the scale) indicates that no glucose has been consumed in the depicted part of the brain. The highest value (at the top of the scale) indicates maximal glucose consumption. Some parts of the brain (e.g., ventricles, in the center of the brain) are filled with brain liquor (basically water), and these regions naturally have a much lower glucose density.

The question: *A researcher presents you with the image below. The researcher claims that the depicted brain shows a normal consciousness state of a person, i.e., a person without any neurological problem that may disrupt normal brain activity.*

Using the response scale below the image, please rate how much you support the researcher's claim based on the image that shows the brain activity.

If you tick the box on the far left side, you do not believe that the image supports the claim at all. If you tick the box on the far right side, you believe that the image fully supports the claim. Tick the box that you believe represents the level of support for the made claim best.

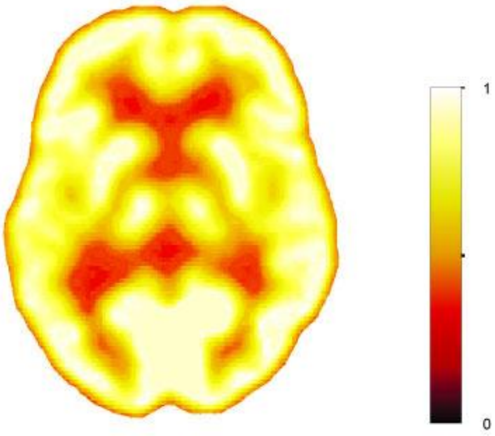

The image supports this claim not at all | ◯ ◯ ◯ ◯ ◯ ◯ ◯ ◯ | The image supports this claim very much

Evaluating neuroimages

Please read the following text carefully before responding.

The color-scale on the right side of the image depicts the level of glucose consumption: the lowest value (at the bottom of the scale) indicates that no glucose has been consumed in the depicted part of the brain. The highest value (at the top of the scale) indicates maximal glucose consumption. Some parts of the brain (e.g., ventricles, in the center of the brain) are filled with brain liquor (basically water), and these regions naturally have a much lower glucose density.

The question: *A researcher presents you with the image below. The researcher claims that the depicted brain is dead, i.e., the blood flow to the brain is blocked such that no oxygen can reach the brain. As a consequence, the brain cells cannot consume glucose any longer, and the cells are in a process of disintegration.*

Using the response scale below the image, please rate how much you support the researcher's claim based on the image that shows the brain activity.

If you tick the box on the far left side, you do not believe that the image supports the claim at all. If you tick the box on the far right side, you believe that the image fully supports the claim. Tick the box that you believe represents the level of support for the made claim best.

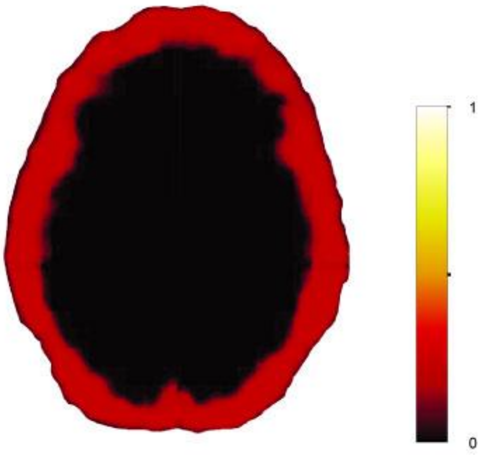

The image supports this claim not at all | ○ ○ ○ ○ ○ ○ ○ ○ | The image supports this claim very much

Evaluating neuroimages

Please read the following text carefully before responding.

In the beginning, you have seen two PET-images that show two extreme states with respect to brain activity: The first image depicted the brain of a normal, conscious person; the second image showed a brain of a brain dead person. We now present you with an image depicting a brain with a neurological problem.

The question: *A researcher presents you with the image below. The researcher claims that the depicted brain is in a so-called locked-in state, i.e., the brain of the person has a more or less normal consciousness state, but due to a neurological problem the person is unable to move and to communicate with the environment. This state shows reduced brain activity when measured with PET.*

Using the response scale below indicate the brain's consciousness state. The response scale shows on the far left side zero brain activity (i.e., brain death; "worst state"), and on the far right side normal, conscious brain activity ("best state").

If you tick the box on the far left side, you believe that the image shows a dead brain (no brain activity), if you tick the box on the far right side, you believe that the image shows a fully conscious, normal brain (normal brain activity). Tick the box you think best shows the brain's consciousness state.

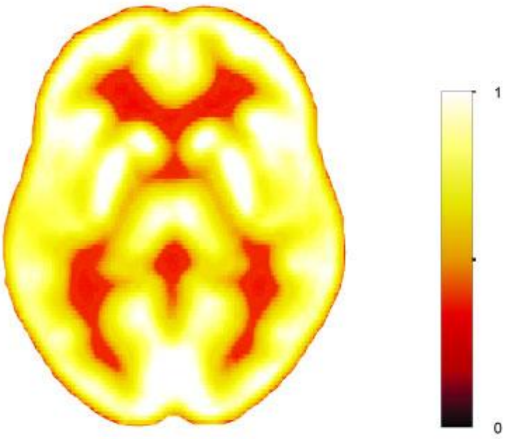

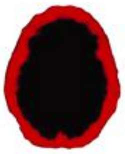

"worst state" | ○ ○ ○ ○ ○ ○ ○ ○ |

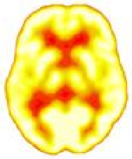

"best state"

Evaluating neuroimages

Please read the following text carefully before responding.

In the beginning, you have seen two PET-images that show two extreme states with respect to brain activity: The first image depicted the brain of a normal, conscious person; the second image showed a brain of a dead person. We now present you with an image depicting a brain with a neurological problem.

The question: *A researcher presents you with the image below. The researcher claims that the depicted brain is in a so-called minimally conscious state, i.e. the person has a severely damaged brain, but still has partial preservation of consciousness. The person also displays minimal but clear behavioral evidence of self / environmental awareness. This state*

shows reduced brain activity when measured with PET.

Using the response scale below indicate the brain's consciousness state. The response scale shows on the far left side zero brain activity (i.e., brain death; "worst state"), and on the far right side normal, conscious brain activity ("best state").

If you tick the box on the far left side, you believe that the image shows a dead brain (no brain activity), if you tick the box on the far right side, you believe that the image shows a fully conscious, normal brain (normal brain activity). Tick the box you think best shows the brain's consciousness state.

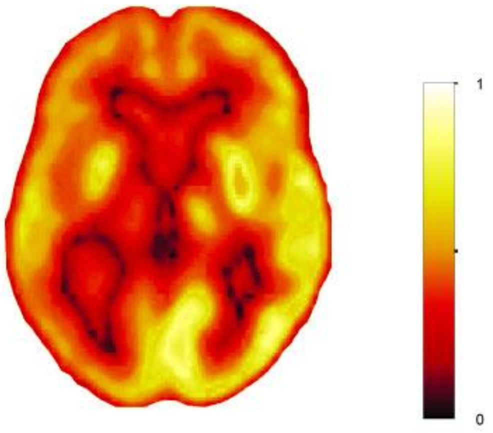

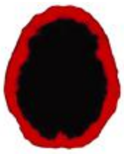

"worst state"

○ ○ ○ ○ ○ ○ ○ ○

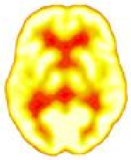

"best state"

Evaluating neuroimages

Please read the following text carefully before responding.

In the beginning, you have seen two PET-images that show two extreme states with respect to brain activity: The first image depicted the brain of a normal, conscious person; the second image showed a brain of a dead person. We now present you with an image depicting a brain with a neurological problem.

The question: *A researcher presents you with the image below. The researcher claims that the depicted brain is in a so-called vegetative state, i.e., the person has a severely damaged brain which results in a state of partial arousal rather than true awareness. The person may open the eyes occasionally, and may demonstrate sleep-wake cycles, but completely lacks cognitive functions. This state shows reduced brain activity when measured with PET.*

Using the response scale below indicate the brain's consciousness state. The response scale shows on the far left side zero brain activity (i.e., brain death; "worst state"), and on the far right side normal, conscious brain activity ("best state").

If you tick the box on the far left side, you believe that the image shows a dead brain (no brain activity), if you tick the box on the far right side, you believe that the image shows a fully conscious, normal brain (normal brain activity). Tick the box you think best shows the brain's consciousness state.

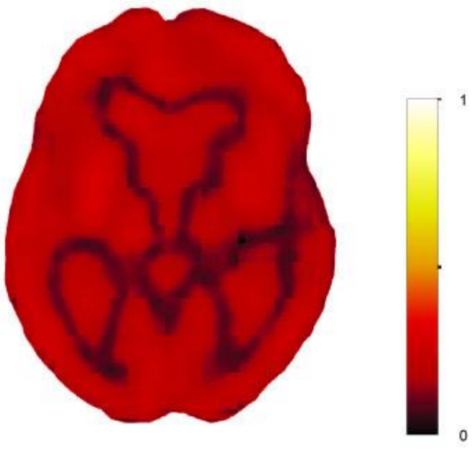

○ ○ ○ ○ ○ ○ ○ ○

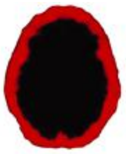

"worst state"

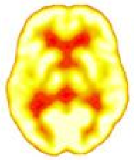

"best state"

Exp Geo rainbow black

Evaluating geoimages

For the next five questions, we ask you to evaluate images that have been produced using techniques to measure the impact of human activity on the ecosystem in a given country. But before that, we would like to know your opinion on the following matter:

"The climate change we experience now is caused by human activities."

Please indicate to what extent you agree with this position:

◯ I completely agree; human activites (burning fossil fuels, etc.) cause climate change

◯ I somewhat agree; human activities contribute partly to climate change

◯ I somewhat disagree; human activities only marginally contribute to climate change

◯ I completely disagree; human activities do not contribute to climate change

◯ I have no opinion on this matter

Evaluating geoimages

Please read the following text carefully before responding.

Below you see an image that has been produced using a method called "environmental sustainability index" (ESI). With this method, the following parameters are integrated into a predictive ecosystem health model: natural resource endowments, past and present pollution levels, environmental management efforts, contributions to protection of the global commons, and a society's capacity to improve its environmental performance over time. The ESI model predicts the health of an ecosystem on a fine-grained regional scale. In below example, the model calculations have been performed for the Netherlands.

The color-scale on the right side of the image depicts the normalized ESI index values that result from model calculations: the lowest value (at the bottom of the scale) indicates that the ecosystem has basically collapsed, such that sustainable human life is impossible. The highest value (at the top of the scale) indicates an optimal, healthy ecosystem state. Some parts in the modeled region (e.g., city centers, sand dunes, etc.) naturally have lower ESI index values.

The question: *A researcher presents you with the image below. The researcher claims that the depicted region shows a normal healthy ecosystem state, i.e., a country without any ecological problems that may disrupt human life.*

Using the response scale below the image, please rate how much you support the researcher's claim based on the image that shows the ecological state. If you tick the box on the far left side, you do not believe that the image supports the claim at all.

If you tick the box on the far right side, you believe that the image fully supports the claim. Tick the box that you believe represents the level of support for the made claim best.

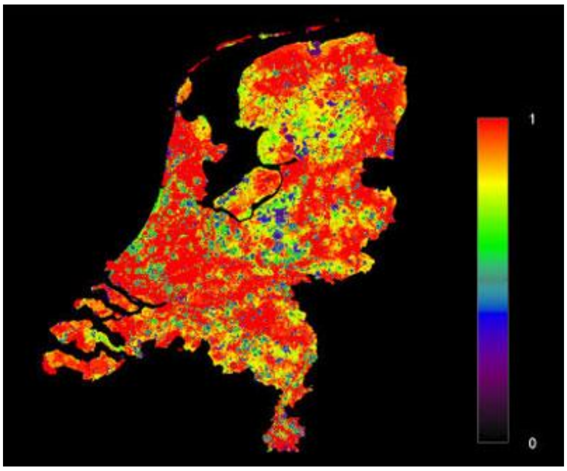

The image supports this claim not at all | ◯ ◯ ◯ ◯ ◯ ◯ ◯ ◯ | The image supports this claim very much

Evaluating geoimages

Please read the following text carefully before responding.

The color-scale on the right side of the image depicts the normalized ESI index values that result from model calculations: the lowest value (at the bottom of the scale) indicates that the ecosystem has basically collapsed, such that sustainable

human life is impossible. The highest value (at the top of the scale) indicates an optimal, healthy ecosystem state. Some parts in the modeled region (e.g., city centers, sand dunes, etc.) naturally have lower ESI index values.

The question: *A researcher presents you with the image below. The researcher claims that the depicted region shows a collapsed ecosystem, i.e., a country that is ecologically dead. Should a country reach this stage, human life would not be possible anymore.*

Using the response scale below the image, please rate how much you support the researcher's claim based on the image that shows the ecological state. If you tick the box on the far left side, you do not believe that the image supports the claim at all.

If you tick the box on the far right side, you believe that the image fully supports the claim. Tick the box that you believe represents the level of support for the made claim best.

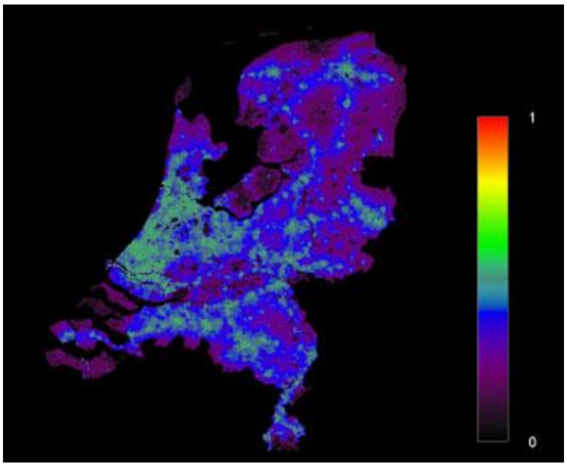

The image supports this claim not at all ○ ○ ○ ○ ○ ○ ○ ○ The image supports this claim very much

Evaluating geoimages

Please read the following text carefully before responding.

In the beginning, you have seen two ESI-images that show two extreme states with respect to a country's ecosystem health: The first image depicted the Netherlands in optimal ecological health state; the second image showed the Netherlands as an ecologically dead country. We now present you with an image depicting a scenario for the Netherlands with an ecological problem.

The question: *A researcher presents you with the image below. The researcher claims that the depicted scenario is in a so-called tipping-point state, i.e., the region has a more or less normal ecosystem health state, but appearing ecological problems in some areas may trigger a downward-trend that would be hard to reverse - nevertheless, recovery is still likely, if the right measures are taken.*

Using the response scale below indicate the country's ecological health state. The response scale shows on the far left that the ecosystem has basically collapsed, such that sustainable human life is impossible (i.e., "worst state"), and on the far right side an optimal, healthy ecosystem ("best state").

If you tick the box on the far left side, you believe that the image shows ecological death (no human life is possible) in the region, if you tick the box on the far right side, you believe that the image shows a fully functional ecosystem (normal ecosystem state). Tick the box you think best shows the country's ecosystem state.

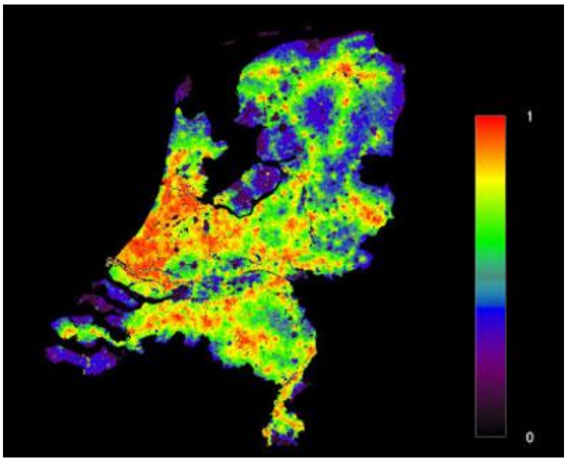

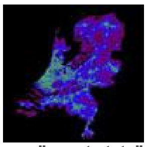

"worst state" ○ ○ ○ ○ ○ ○ ○ ○

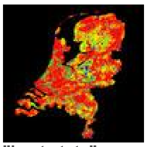

"best state"

Evaluating geoimages

Please read the following text carefully before responding.

In the beginning, you have seen two ESI-images that show two extreme states with respect to a country's ecosystem health: The first image depicted the Netherlands in optimal ecological health state; the second image showed the Netherlands as an ecologically dead country. We now present you with an image depicting a scenario for the Netherlands with an ecological problem.

The question: *A researcher presents you with the image below. The researcher claims that the depicted scenario shows the region in a minimal ecosystem health state, where large parts of the region display unfavorable ESI values. In such an ecosystem health state, there is a substantial risk that the region becomes unlivable unless radical measures are taken.*

Using the response scale below indicate the country's ecological health state. The response scale shows on the far left that the ecosystem has basically collapsed, such that sustainable human life is impossible (i.e., "worst state"), and on the far right side an optimal, healthy ecosystem ("best state").

If you tick the box on the far left side, you believe that the image shows ecological death (no human life is possible) in the region, if you tick the box on the far right side, you believe that the image shows a fully functional ecosystem (normal ecosystem state). Tick the box you think best shows the country's ecosystem state.

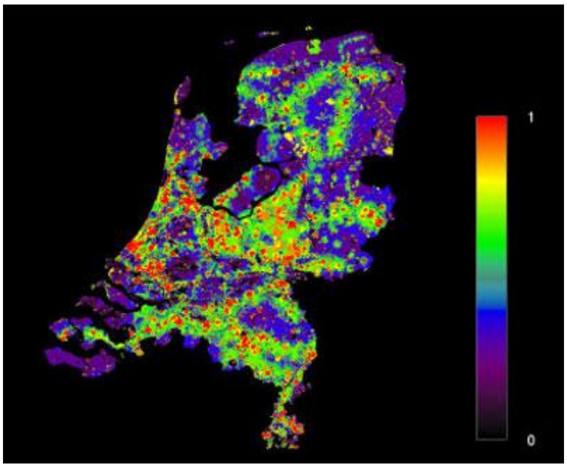

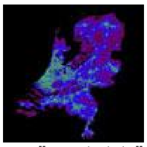

"worst state"

○ ○ ○ ○ ○ ○ ○ ○

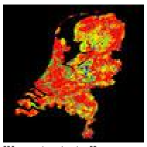

"best state"

Evaluating geoimages

Please read the following text carefully before responding.

In the beginning, you have seen two ESI-images that show two extreme states with respect to a country's ecosystem health: The first image depicted the Netherlands in optimal ecological health state; the second image showed the Netherlands as an ecologically dead country. We now present you with an image depicting a scenario for the Netherlands with an ecological Problem.

The question: *A researcher presents you with the image below. The researcher claims that the depicted scenario shows the region in a so-called beyond recovery state, where most parts of the region display unfavorable ESI values. Human existence is still possible but at bare minimum levels.*

Using the response scale below indicate the country's ecological health state. The response scale shows on the far left that the ecosystem has basically collapsed, such that sustainable human life is impossible (i.e., "worst state"), and on the far right side an optimal, healthy ecosystem ("best state").

If you tick the box on the far left side, you believe that the image shows ecological death (no human life is possible) in the region, if you tick the box on the far right side, you believe that the image shows a fully functional ecosystem (normal ecosystem state). Tick the box you think best shows the country's ecosystem state.

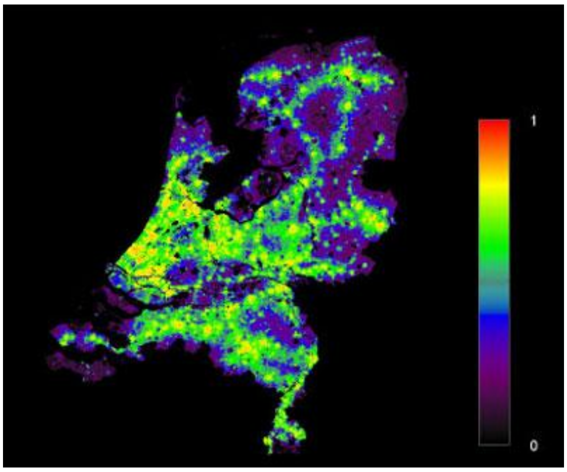

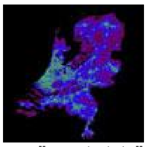

"worst state"

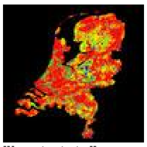

"best state"

Exp Geo rainbow white

Evaluating geoimages

For the next five questions, we ask you to evaluate images that have been produced using techniques to measure the impact of human activity on the ecosystem in a given country. But before that, we would like to know your opinion on the following matter:

"The climate change we experience now is caused by human activities."

Please indicate to what extent you agree with this position:

- I completely agree; human activites (burning fossil fuels, etc.) cause climate change
- I somewhat agree; human activities contribute partly to climate change
- I somewhat disagree; human activities only marginally contribute to climate change
- I completely disagree; human activities do not contribute to climate change
- I have no opinion on this matter

Evaluating geoimages

Please read the following text carefully before responding.

Below you see an image that has been produced using a method called "environmental sustainability index" (ESI). With this method, the following parameters are integrated into a predictive ecosystem health model: natural resource endowments, past and present pollution levels, environmental management efforts, contributions to protection of the global commons, and a society's capacity to improve its environmental performance over time. The ESI model predicts the health of an ecosystem on a fine-grained regional scale. In below example, the model calculations have been performed for the Netherlands.

The color-scale on the right side of the image depicts the normalized ESI index values that result from model calculations: the lowest value (at the bottom of the scale) indicates that the ecosystem has basically collapsed, such that sustainable human life is impossible. The highest value (at the top of the scale) indicates an optimal, healthy ecosystem state. Some parts in the modeled region (e.g., city centers, sand dunes, etc.) naturally have lower ESI index values.

The question: *A researcher presents you with the image below. The researcher claims that the depicted region shows a normal healthy ecosystem state, i.e., a country without any ecological problems that may disrupt human life.*

Using the response scale below the image, please rate how much you support the researcher's claim based on the image that shows the ecological state. If you tick the box on the far left side, you do not believe that the image supports the claim at all.

If you tick the box on the far right side, you believe that the image fully supports the claim. Tick the box that you believe represents the level of support for the made claim best.

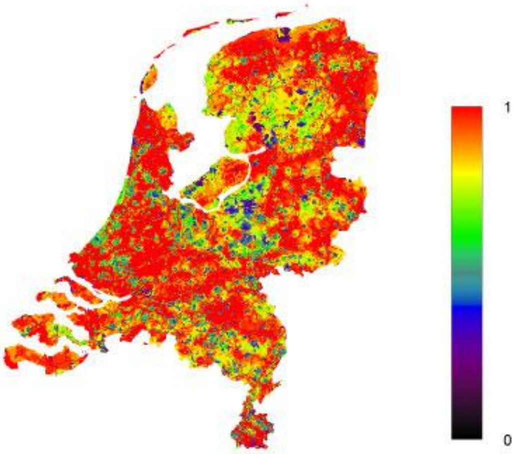

The image supports this claim not at all ○ ○ ○ ○ ○ ○ ○ ○ The image supports this claim very much

Evaluating geoimages

Please read the following text carefully before responding.

The color-scale on the right side of the image depicts the normalized ESI index values that result from model calculations: the lowest value (at the bottom of the scale) indicates that the ecosystem has basically collapsed, such that sustainable human life is impossible. The highest value (at the top of the scale) indicates an optimal, healthy ecosystem state. Some parts in the modeled region (e.g., city centers, sand dunes, etc.) naturally have lower ESI index values.

The question: *A researcher presents you with the image below. The researcher claims that the depicted region shows a collapsed ecosystem, i.e., a country that is ecologically dead. Should a country reach this stage, human life would not be possible anymore.*

Using the response scale below the image, please rate how much you support the researcher’s claim based on the image that shows the ecological state. If you tick the box on the far left side, you do not believe that the image supports the claim at all.

If you tick the box on the far right side, you believe that the image fully supports the claim. Tick the box that you believe represents the level of support for the made claim best.

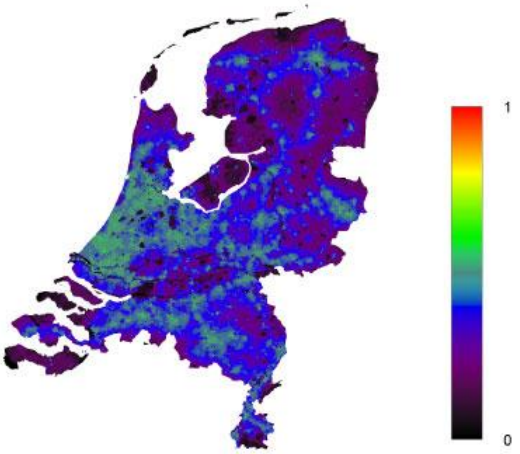

The image supports this claim not at all ○ ○ ○ ○ ○ ○ ○ ○ The image supports this claim very much

Evaluating geoimages

Please read the following text carefully before responding.

In the beginning, you have seen two ESI-images that show two extreme states with respect to a country's ecosystem health: The first image depicted the Netherlands in optimal ecological health state; the second image showed the Netherlands as an ecologically dead country. We now present you with an image depicting a scenario for the Netherlands with an ecological problem.

The question: *A researcher presents you with the image below. The researcher claims that the depicted scenario is in a so-called tipping-point state, i.e., the region has a more or less normal ecosystem health state, but appearing ecological problems in some areas may trigger a downward-trend that would be hard to reverse - nevertheless, recovery is still likely, if the right measures are taken.*

Using the response scale below indicate the country’s ecological health state. The response scale shows on the far left that

the ecosystem has basically collapsed, such that sustainable human life is impossible (i.e., "worst state"), and on the far right side an optimal, healthy ecosystem ("best state").

If you tick the box on the far left side, you believe that the image shows ecological death (no human life is possible) in the region, if you tick the box on the far right side, you believe that the image shows a fully functional ecosystem (normal ecosystem state). Tick the box you think best shows the country's ecosystem state.

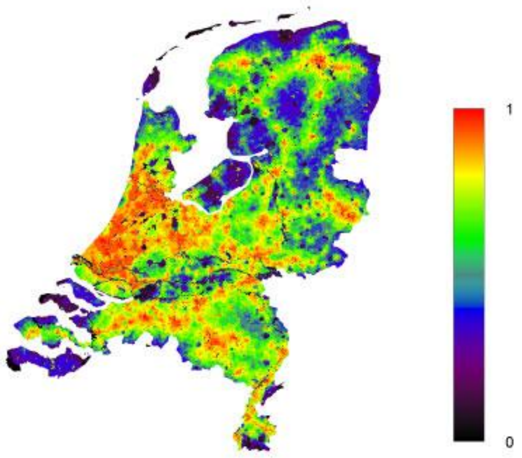

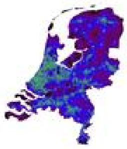

"worst state"

○ ○ ○ ○ ○ ○ ○ ○

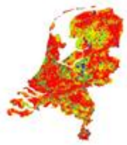

"best state"

Evaluating geoimages

Please read the following text carefully before responding.

In the beginning, you have seen two ESI-images that show two extreme states with respect to a country's ecosystem health: The first image depicted the Netherlands in optimal ecological health state; the second image showed the Netherlands as an ecologically dead country. We now present you with an image depicting a scenario for the Netherlands with an ecological problem.

The question: *A researcher presents you with the image below. The researcher claims that the depicted scenario shows the region in a minimal ecosystem health state, where large parts of the region display unfavorable ESI values. In such an ecosystem health state, there is a substantial risk that the region becomes unlivable unless radical measures are taken.*

Using the response scale below indicate the country's ecological health state. The response scale shows on the far left that the ecosystem has basically collapsed, such that sustainable human life is impossible (i.e., "worst state"), and on the far right side an optimal, healthy ecosystem ("best state").

If you tick the box on the far left side, you believe that the image shows ecological death (no human life is possible) in the region, if you tick the box on the far right side, you believe that the image shows a fully functional ecosystem (normal ecosystem state). Tick the box you think best shows the country's ecosystem state.

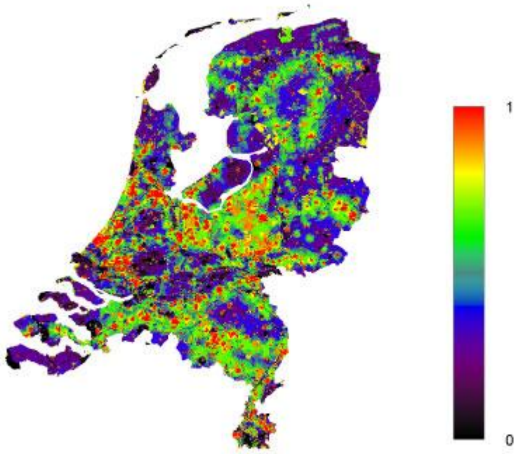

○ ○ ○ ○ ○ ○ ○ ○

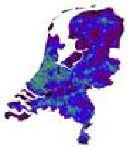

"worst state"

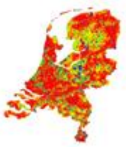

"best state"

Evaluating geoimages

Please read the following text carefully before responding.

In the beginning, you have seen two ESI-images that show two extreme states with respect to a country's ecosystem health: The first image depicted the Netherlands in optimal ecological health state; the second image showed the Netherlands as an ecologically dead country. We now present you with an image depicting a scenario for the Netherlands with an ecological Problem.

The question: *A researcher presents you with the image below. The researcher claims that the depicted scenario shows the region in a so-called beyond recovery state, where most parts of the region display unfavorable ESI values. Human existence is still possible but at bare minimum levels.*

Using the response scale below indicate the country's ecological health state. The response scale shows on the far left that the ecosystem has basically collapsed, such that sustainable human life is impossible (i.e., "worst state"), and on the far right side an optimal, healthy ecosystem ("best state").

If you tick the box on the far left side, you believe that the image shows ecological death (no human life is possible) in the region, if you tick the box on the far right side, you believe that the image shows a fully functional ecosystem (normal ecosystem state). Tick the box you think best shows the country's ecosystem state.

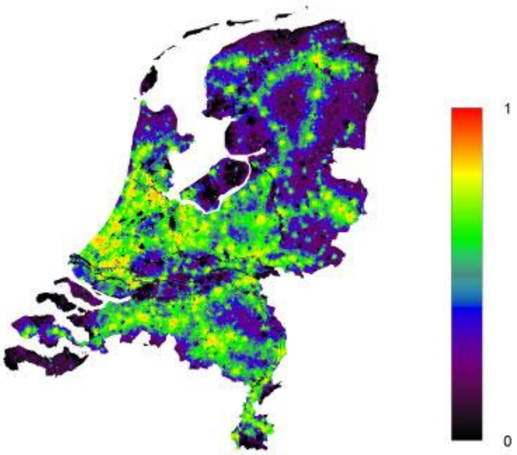

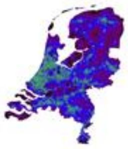

"worst state"

○ ○ ○ ○ ○ ○ ○ ○

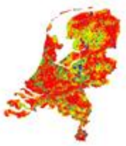

"best state"

Exp Geo blue-red black

Evaluating geoimages

For the next five questions, we ask you to evaluate images that have been produced using techniques to measure the impact of human activity on the ecosystem in a given country. But before that, we would like to know your opinion on the following matter:

"The climate change we experience now is caused by human activities."

Please indicate to what extent you agree with this position:

- ○ I completely agree; human activites (burning fossil fuels, etc.) cause climate change
- ○ I somewhat agree; human activities contribute partly to climate change
- ○ I somewhat disagree; human activities only marginally contribute to climate change
- ○ I completely disagree; human activities do not contribute to climate change
- ○ I have no opinion on this matter

Evaluating geoimages

Please read the following text carefully before responding.

Below you see an image that has been produced using a method called "environmental sustainability index" (ESI). With this method, the following parameters are integrated into a predictive ecosystem health model: natural resource endowments, past and present pollution levels, environmental management efforts, contributions to protection of the global commons, and a society's capacity to improve its environmental performance over time. The ESI model predicts the health of an ecosystem on a fine-grained regional scale. In below example, the model calculations have been performed for the Netherlands.

The color-scale on the right side of the image depicts the normalized ESI index values that result from model calculations: the lowest value (at the bottom of the scale) indicates that the ecosystem has basically collapsed, such that sustainable human life is impossible. The highest value (at the top of the scale) indicates an optimal, healthy ecosystem state. Some parts in the modeled region (e.g., city centers, sand dunes, etc.) naturally have lower ESI index values.

The question: *A researcher presents you with the image below. The researcher claims that the depicted region shows a normal healthy ecosystem state, i.e., a country without any ecological problems that may disrupt human life.*

Using the response scale below the image, please rate how much you support the researcher's claim based on the image that shows the ecological state. If you tick the box on the far left side, you do not believe that the image supports the claim at all.

If you tick the box on the far right side, you believe that the image fully supports the claim. Tick the box that you believe represents the level of support for the made claim best.

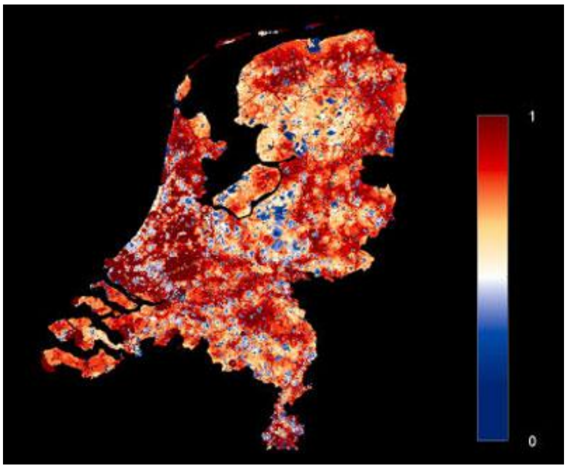

The image supports this claim not at all ○ ○ ○ ○ ○ ○ ○ ○ The image supports this claim very much

Evaluating geoimages

Please read the following text carefully before responding.

The color-scale on the right side of the image depicts the normalized ESI index values that result from model calculations: the lowest value (at the bottom of the scale) indicates that the ecosystem has basically collapsed, such that sustainable human life is impossible. The highest value (at the top of the scale) indicates an optimal, healthy ecosystem state. Some parts in the modeled region (e.g., city centers, sand dunes, etc.) naturally have lower ESI index values.

The question: *A researcher presents you with the image below. The researcher claims that the depicted region shows a collapsed ecosystem, i.e., a country that is ecologically dead. Should a country reach this stage, human life would not be possible anymore.*

Using the response scale below the image, please rate how much you support the researcher's claim based on the image that shows the ecological state. If you tick the box on the far left side, you do not believe that the image supports the claim at all.

If you tick the box on the far right side, you believe that the image fully supports the claim. Tick the box that you believe represents the level of support for the made claim best.

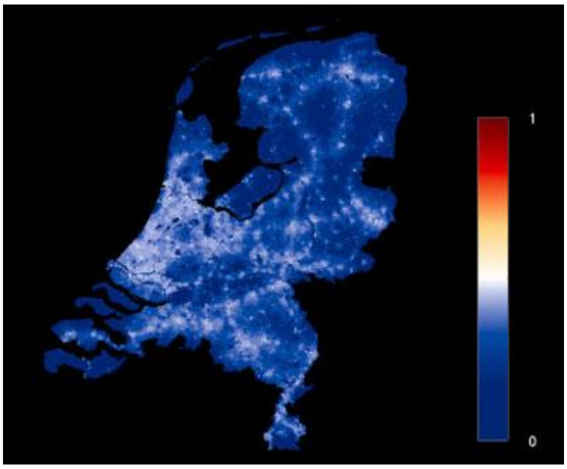

The image supports this claim not at all ○ ○ ○ ○ ○ ○ ○ ○ The image supports this claim very much

Evaluating geoimages

Please read the following text carefully before responding.

In the beginning, you have seen two ESI-images that show two extreme states with respect to a country's ecosystem health: The first image depicted the Netherlands in optimal ecological health state; the second image showed the Netherlands as an ecologically dead country. We now present you with an image depicting a scenario for the Netherlands with an ecological problem.

The question: *A researcher presents you with the image below. The researcher claims that the depicted scenario is in a so-called tipping-point state, i.e., the region has a more or less normal ecosystem health state, but appearing ecological problems in some areas may trigger a downward-trend that would be hard to reverse - nevertheless, recovery is still likely, if the right measures are taken.*

Using the response scale below indicate the country’s ecological health state. The response scale shows on the far left that the ecosystem has basically collapsed, such that sustainable human life is impossible (i.e., "worst state"), and on the far right side an optimal, healthy ecosystem ("best state").

If you tick the box on the far left side, you believe that the image shows ecological death (no human life is possible) in the region, if you tick the box on the far right side, you believe that the image shows a fully functional ecosystem (normal ecosystem state). Tick the box you think best shows the country’s ecosystem state.

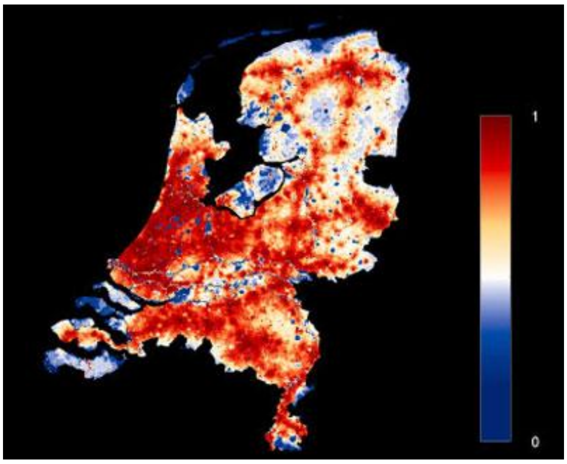

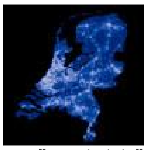

"worst state"

○ ○ ○ ○ ○ ○ ○ ○

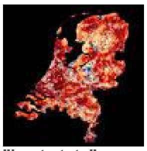

"best state"

Evaluating geoimages

Please read the following text carefully before responding.

In the beginning, you have seen two ESI-images that show two extreme states with respect to a country's ecosystem health: The first image depicted the Netherlands in optimal ecological health state; the second image showed the Netherlands as an ecologically dead country. We now present you with an image depicting a scenario for the Netherlands with an ecological problem.

The question: *A researcher presents you with the image below. The researcher claims that the depicted scenario shows the region in a minimal ecosystem health state, where large parts of the region display unfavorable ESI values. In such an ecosystem health state, there is a substantial risk that the region becomes unlivable unless radical measures are taken.*

Using the response scale below indicate the country’s ecological health state. The response scale shows on the far left that the ecosystem has basically collapsed, such that sustainable human life is impossible (i.e., "worst state"), and on the far right side an optimal, healthy ecosystem ("best state").

If you tick the box on the far left side, you believe that the image shows ecological death (no human life is possible) in the region, if you tick the box on the far right side, you believe that the image shows a fully functional ecosystem (normal ecosystem state). Tick the box you think best shows the country’s ecosystem state.

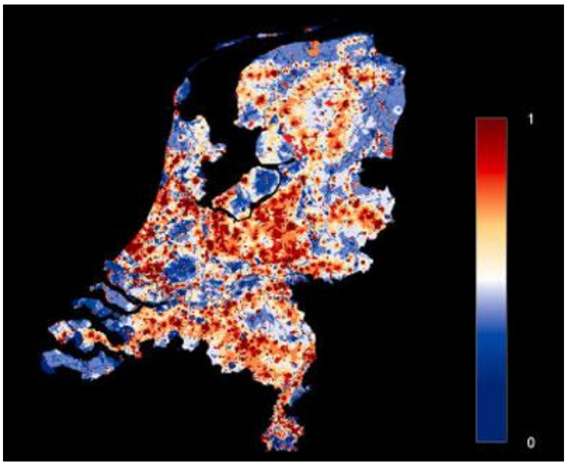

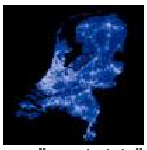
"worst state"

○ ○ ○ ○ ○ ○ ○ ○

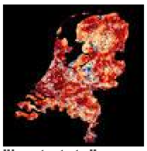
"best state"

Evaluating geoimages

Please read the following text carefully before responding.

In the beginning, you have seen two ESI-images that show two extreme states with respect to a country's ecosystem health: The first image depicted the Netherlands in optimal ecological health state; the second image showed the Netherlands as an ecologically dead country. We now present you with an image depicting a scenario for the Netherlands with an ecological Problem.

The question: *A researcher presents you with the image below. The researcher claims that the depicted scenario shows the region in a so-called beyond recovery state, where most parts of the region display unfavorable ESI values. Human existence is still possible but at bare minimum levels.*

Using the response scale below indicate the country's ecological health state. The response scale shows on the far left that the ecosystem has basically collapsed, such that sustainable human life is impossible (i.e., "worst state"), and on the far right side an optimal, healthy ecosystem ("best state").

If you tick the box on the far left side, you believe that the image shows ecological death (no human life is possible) in the region, if you tick the box on the far right side, you believe that the image shows a fully functional ecosystem (normal ecosystem state). Tick the box you think best shows the country's ecosystem state.

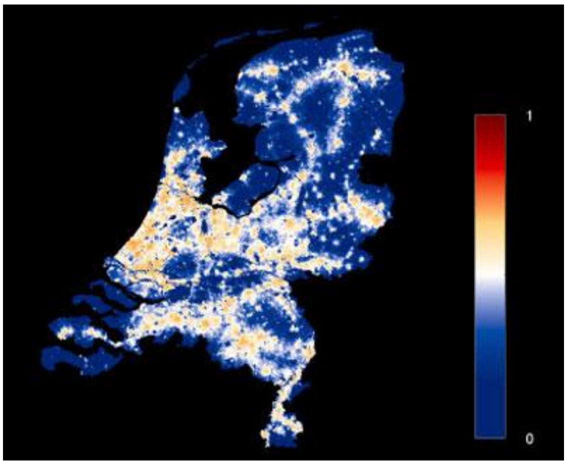

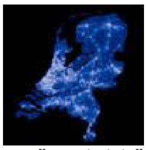
"worst state"

○ ○ ○ ○ ○ ○ ○ ○

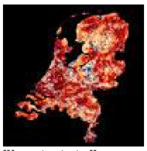
"best state"

Exp Geo blue-red white

Evaluating geoimages

For the next five questions, we ask you to evaluate images that have been produced using techniques to measure the impact of human activity on the ecosystem in a given country. But before that, we would like to know your opinion on the following matter:

"The climate change we experience now is caused by human activities."

Please indicate to what extent you agree with this position:

- ◯ I completely agree; human activites (burning fossil fuels, etc.) cause climate change
- ◯ I somewhat agree; human activities contribute partly to climate change
- ◯ I somewhat disagree; human activities only marginally contribute to climate change
- ◯ I completely disagree; human activities do not contribute to climate change
- ◯ I have no opinion on this matter

Evaluating geoimages

Please read the following text carefully before responding.

Below you see an image that has been produced using a method called "environmental sustainability index" (ESI). With this method, the following parameters are integrated into a predictive ecosystem health model: natural resource endowments, past and present pollution levels, environmental management efforts, contributions to protection of the global commons, and a society's capacity to improve its environmental performance over time. The ESI model predicts the health of an ecosystem on a fine-grained regional scale. In below example, the model calculations have been performed for the Netherlands.

The color-scale on the right side of the image depicts the normalized ESI index values that result from model calculations: the lowest value (at the bottom of the scale) indicates that the ecosystem has basically collapsed, such that sustainable human life is impossible. The highest value (at the top of the scale) indicates an optimal, healthy ecosystem state. Some parts in the modeled region (e.g., city centers, sand dunes, etc.) naturally have lower ESI index values.

The question: *A researcher presents you with the image below. The researcher claims that the depicted region shows a normal healthy ecosystem state, i.e., a country without any ecological problems that may disrupt human life.*

Using the response scale below the image, please rate how much you support the researcher's claim based on the image that shows the ecological state. If you tick the box on the far left side, you do not believe that the image supports the claim at all.

If you tick the box on the far right side, you believe that the image fully supports the claim. Tick the box that you believe represents the level of support for the made claim best.

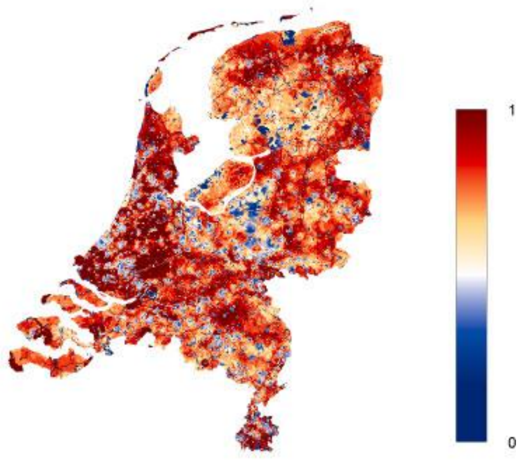

The image supports this claim not at all | ◯ ◯ ◯ ◯ ◯ ◯ ◯ ◯ | The image supports this claim very much

Evaluating geoimages

Please read the following text carefully before responding.

The color-scale on the right side of the image depicts the normalized ESI index values that result from model calculations: the lowest value (at the bottom of the scale) indicates that the ecosystem has basically collapsed, such that sustainable human life is impossible. The highest value (at the top of the scale) indicates an optimal, healthy ecosystem state. Some parts in the modeled region (e.g., city centers, sand dunes, etc.) naturally have lower ESI index values.

The question: *A researcher presents you with the image below. The researcher claims that the depicted region shows a collapsed ecosystem, i.e., a country that is ecologically dead. Should a country reach this stage, human life would not be possible anymore.*

Using the response scale below the image, please rate how much you support the researcher's claim based on the image that shows the ecological state. If you tick the box on the far left side, you do not believe that the image supports the claim at all.

If you tick the box on the far right side, you believe that the image fully supports the claim. Tick the box that you believe represents the level of support for the made claim best.

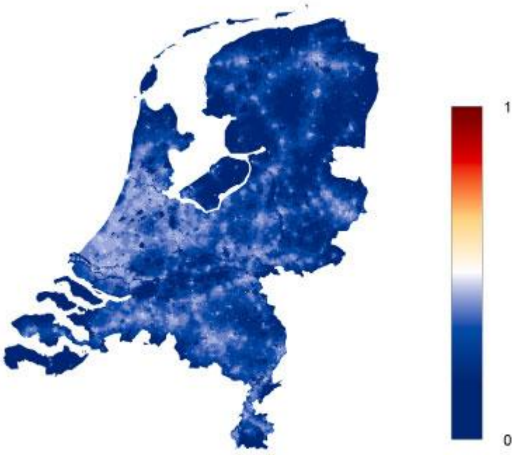

The image supports this claim not at all | ○ ○ ○ ○ ○ ○ ○ ○ | The image supports this claim very much

Evaluating geoimages

Please read the following text carefully before responding.

In the beginning, you have seen two ESI-images that show two extreme states with respect to a country's ecosystem health: The first image depicted the Netherlands in optimal ecological health state; the second image showed the Netherlands as an ecologically dead country. We now present you with an image depicting a scenario for the Netherlands with an ecological problem.

The question: *A researcher presents you with the image below. The researcher claims that the depicted scenario is in a so-called tipping-point state, i.e., the region has a more or less normal ecosystem health state, but appearing ecological problems in some areas may trigger a downward-trend that would be hard to reverse - nevertheless, recovery is still likely, if the right measures are taken.*

Using the response scale below indicate the country’s ecological health state. The response scale shows on the far left that the ecosystem has basically collapsed, such that sustainable human life is impossible (i.e., "worst state"), and on the far right side an optimal, healthy ecosystem ("best state").

If you tick the box on the far left side, you believe that the image shows ecological death (no human life is possible) in the region, if you tick the box on the far right side, you believe that the image shows a fully functional ecosystem (normal ecosystem state). Tick the box you think best shows the country’s ecosystem state.

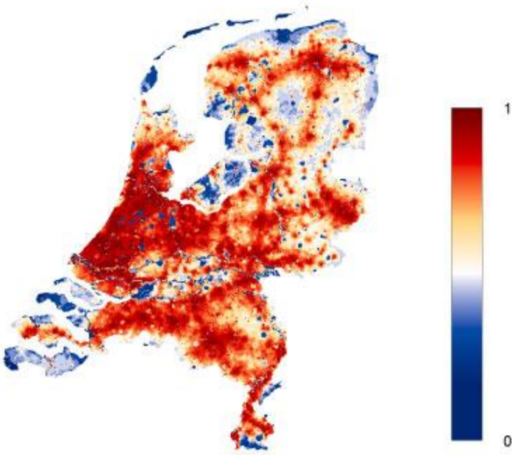

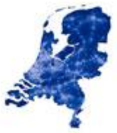

"worst state" | ○ ○ ○ ○ ○ ○ ○ ○ |

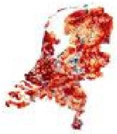

"best state"

Evaluating geoimages

Please read the following text carefully before responding.

In the beginning, you have seen two ESI-images that show two extreme states with respect to a country's ecosystem health: The first image depicted the Netherlands in optimal ecological health state; the second image showed the Netherlands as

an ecologically dead country. We now present you with an image depicting a scenario for the Netherlands with an ecological problem.

The question: *A researcher presents you with the image below. The researcher claims that the depicted scenario shows the region in a minimal ecosystem health state, where large parts of the region display unfavorable ESI values. In such an ecosystem health state, there is a substantial risk that the region becomes unlivable unless radical measures are taken.*

Using the response scale below indicate the country's ecological health state. The response scale shows on the far left that the ecosystem has basically collapsed, such that sustainable human life is impossible (i.e., "worst state"), and on the far right side an optimal, healthy ecosystem ("best state").

If you tick the box on the far left side, you believe that the image shows ecological death (no human life is possible) in the region, if you tick the box on the far right side, you believe that the image shows a fully functional ecosystem (normal ecosystem state). Tick the box you think best shows the country's ecosystem state.

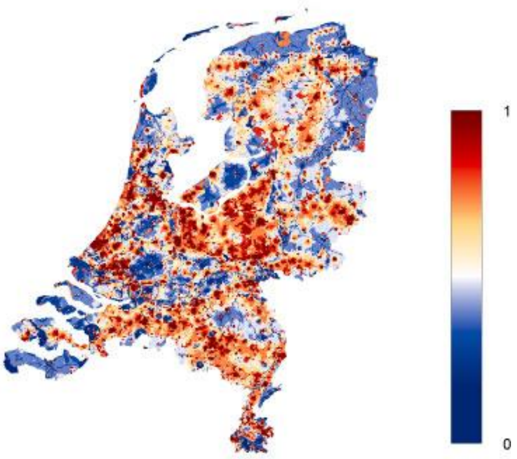

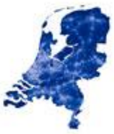

"worst state"

○ ○ ○ ○ ○ ○ ○ ○

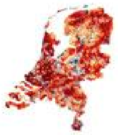

"best state"

Evaluating geoimages

Please read the following text carefully before responding.

In the beginning, you have seen two ESI-images that show two extreme states with respect to a country's ecosystem health: The first image depicted the Netherlands in optimal ecological health state; the second image showed the Netherlands as an ecologically dead country. We now present you with an image depicting a scenario for the Netherlands with an ecological Problem.

The question: *A researcher presents you with the image below. The researcher claims that the depicted scenario shows the region in a so-called beyond recovery state, where most parts of the region display unfavorable ESI values. Human existence is still possible but at bare minimum levels.*

Using the response scale below indicate the country's ecological health state. The response scale shows on the far left that the ecosystem has basically collapsed, such that sustainable human life is impossible (i.e., "worst state"), and on the far right side an optimal, healthy ecosystem ("best state").

If you tick the box on the far left side, you believe that the image shows ecological death (no human life is possible) in the region, if you tick the box on the far right side, you believe that the image shows a fully functional ecosystem (normal ecosystem state). Tick the box you think best shows the country's ecosystem state.

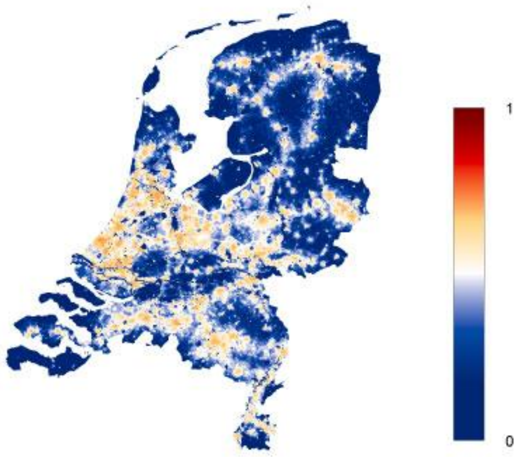

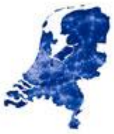

"worst state" ○ ○ ○ ○ ○ ○ ○ ○ "best state"

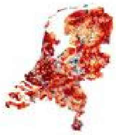

Exp Geo green black

Evaluating geoimages

For the next five questions, we ask you to evaluate images that have been produced using techniques to measure the impact of human activity on the ecosystem in a given country. But before that, we would like to know your opinion on the following matter:

"The climate change we experience now is caused by human activities."

Please indicate to what extent you agree with this position:

○ I completely agree; human activites (burning fossil fuels, etc.) cause climate change

○ I somewhat agree; human activities contribute partly to climate change

○ I somewhat disagree; human activities only marginally contribute to climate change

○ I completely disagree; human activities do not contribute to climate change

○ I have no opinion on this matter

Evaluating geoimages

Please read the following text carefully before responding.

Below you see an image that has been produced using a method called "environmental sustainability index" (ESI). With this method, the following parameters are integrated into a predictive ecosystem health model: natural resource endowments, past and present pollution levels, environmental management efforts, contributions to protection of the global commons, and a society's capacity to improve its environmental performance over time. The ESI model predicts the health of an ecosystem on a fine-grained regional scale. In below example, the model calculations have been performed for the Netherlands.

The color-scale on the right side of the image depicts the normalized ESI index values that result from model calculations: the lowest value (at the bottom of the scale) indicates that the ecosystem has basically collapsed, such that sustainable human life is impossible. The highest value (at the top of the scale) indicates an optimal, healthy ecosystem state. Some parts in the modeled region (e.g., city centers, sand dunes, etc.) naturally have lower ESI index values.

The question: *A researcher presents you with the image below. The researcher claims that the depicted region shows a normal healthy ecosystem state, i.e., a country without any ecological problems that may disrupt human life.*

Using the response scale below the image, please rate how much you support the researcher's claim based on the image that shows the ecological state. If you tick the box on the far left side, you do not believe that the image supports the claim at all.

If you tick the box on the far right side, you believe that the image fully supports the claim. Tick the box that you believe represents the level of support for the made claim best.

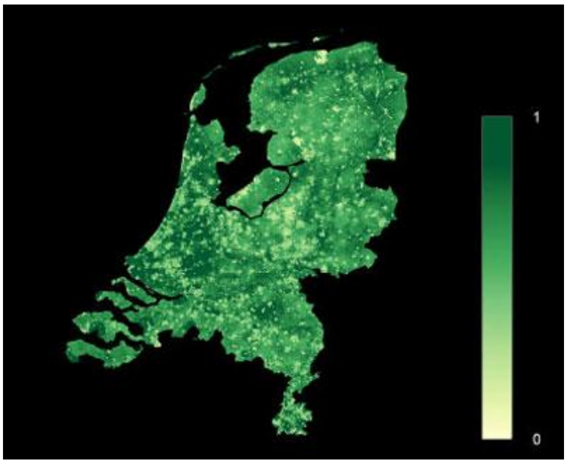

The image supports this claim not at all ○ ○ ○ ○ ○ ○ ○ ○ The image supports this claim very much

Evaluating geoimages

Please read the following text carefully before responding.

The color-scale on the right side of the image depicts the normalized ESI index values that result from model calculations: the lowest value (at the bottom of the scale) indicates that the ecosystem has basically collapsed, such that sustainable human life is impossible. The highest value (at the top of the scale) indicates an optimal, healthy ecosystem state. Some parts in the modeled region (e.g., city centers, sand dunes, etc.) naturally have lower ESI index values.

The question: *A researcher presents you with the image below. The researcher claims that the depicted region shows a collapsed ecosystem, i.e., a country that is ecologically dead. Should a country reach this stage, human life would not be possible anymore.*

Using the response scale below the image, please rate how much you support the researcher's claim based on the image that shows the ecological state. If you tick the box on the far left side, you do not believe that the image supports the claim at all.

If you tick the box on the far right side, you believe that the image fully supports the claim. Tick the box that you believe represents the level of support for the made claim best.

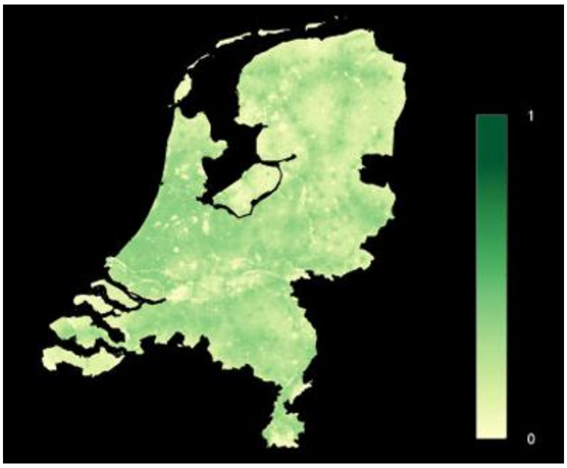

The image supports this claim not at all ○ ○ ○ ○ ○ ○ ○ ○ The image supports this claim very much

Evaluating geoimages

Please read the following text carefully before responding.

In the beginning, you have seen two ESI-images that show two extreme states with respect to a country's ecosystem health: The first image depicted the Netherlands in optimal ecological health state; the second image showed the Netherlands as an ecologically dead country. We now present you with an image depicting a scenario for the Netherlands with an ecological problem.

The question: *A researcher presents you with the image below. The researcher claims that the depicted scenario is in a so-called tipping-point state, i.e., the region has a more or less normal ecosystem health state, but appearing ecological problems in some areas may trigger a downward-trend that would be hard to reverse - nevertheless, recovery is still likely, if the right measures are taken.*

Using the response scale below indicate the country's ecological health state. The response scale shows on the far left that the ecosystem has basically collapsed, such that sustainable human life is impossible (i.e., "worst state"), and on the far right side an optimal, healthy ecosystem ("best state").

If you tick the box on the far left side, you believe that the image shows ecological death (no human life is possible) in the region, if you tick the box on the far right side, you believe that the image shows a fully functional ecosystem (normal ecosystem state). Tick the box you think best shows the country's ecosystem state.

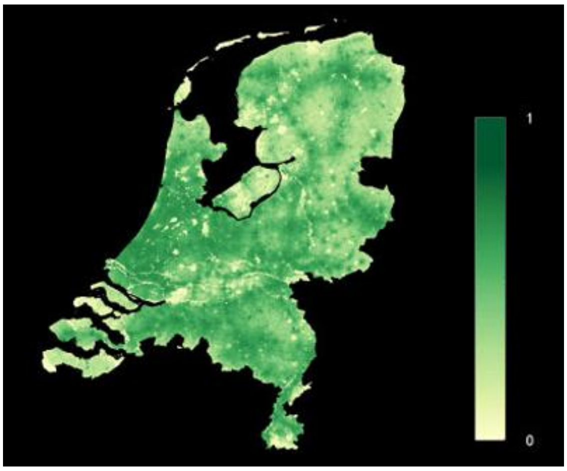

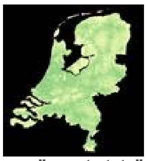

"worst state"

○ ○ ○ ○ ○ ○ ○ ○

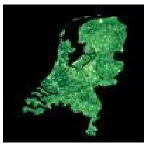

"best state"

Evaluating geoimages

Please read the following text carefully before responding.

In the beginning, you have seen two ESI-images that show two extreme states with respect to a country's ecosystem health: The first image depicted the Netherlands in optimal ecological health state; the second image showed the Netherlands as an ecologically dead country. We now present you with an image depicting a scenario for the Netherlands with an ecological problem.

The question: *A researcher presents you with the image below. The researcher claims that the depicted scenario shows the region in a minimal ecosystem health state, where large parts of the region display unfavorable ESI values. In such an ecosystem health state, there is a substantial risk that the region becomes unlivable unless radical measures are taken.*

Using the response scale below indicate the country's ecological health state. The response scale shows on the far left that the ecosystem has basically collapsed, such that sustainable human life is impossible (i.e., "worst state"), and on the far right side an optimal, healthy ecosystem ("best state").

If you tick the box on the far left side, you believe that the image shows ecological death (no human life is possible) in the region, if you tick the box on the far right side, you believe that the image shows a fully functional ecosystem (normal ecosystem state). Tick the box you think best shows the country's ecosystem state.

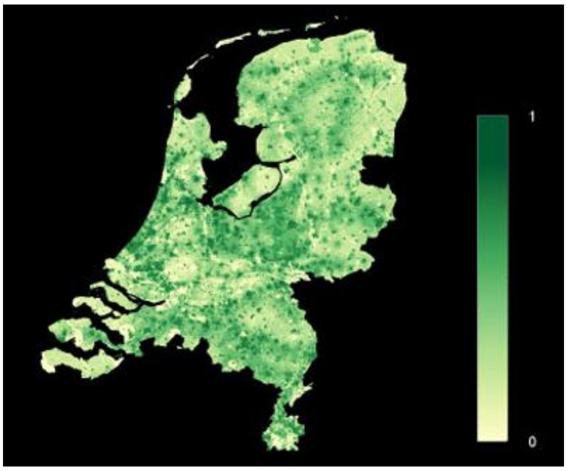

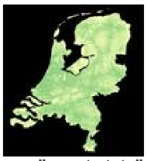

"worst state"

○ ○ ○ ○ ○ ○ ○ ○

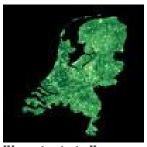

"best state"

Evaluating geoimages

Please read the following text carefully before responding.

In the beginning, you have seen two ESI-images that show two extreme states with respect to a country's ecosystem health: The first image depicted the Netherlands in optimal ecological health state; the second image showed the Netherlands as an ecologically dead country. We now present you with an image depicting a scenario for the Netherlands with an ecological Problem.

The question: *A researcher presents you with the image below. The researcher claims that the depicted scenario shows the region in a so-called beyond recovery state, where most parts of the region display unfavorable ESI values. Human existence is still possible but at bare minimum levels.*

Using the response scale below indicate the country's ecological health state. The response scale shows on the far left that the ecosystem has basically collapsed, such that sustainable human life is impossible (i.e., "worst state"), and on the far right side an optimal, healthy ecosystem ("best state").

If you tick the box on the far left side, you believe that the image shows ecological death (no human life is possible) in the region, if you tick the box on the far right side, you believe that the image shows a fully functional ecosystem (normal ecosystem state). Tick the box you think best shows the country's ecosystem state.

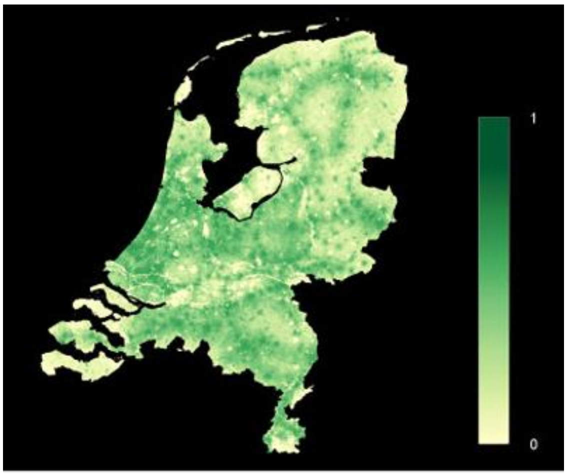

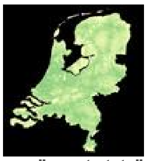
"worst state"

○ ○ ○ ○ ○ ○ ○ ○

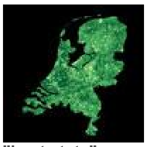
"best state"

Exp Geo green white

Evaluating geoimages

For the next five questions, we ask you to evaluate images that have been produced using techniques to measure the impact of human activity on the ecosystem in a given country. But before that, we would like to know your opinion on the following matter:

"The climate change we experience now is caused by human activities."

Please indicate to what extent you agree with this position:

- ○ I completely agree; human activites (burning fossil fuels, etc.) cause climate change
- ○ I somewhat agree; human activities contribute partly to climate change
- ○ I somewhat disagree; human activities only marginally contribute to climate change
- ○ I completely disagree; human activities do not contribute to climate change
- ○ I have no opinion on this matter

Evaluating geoimages

Please read the following text carefully before responding.

Below you see an image that has been produced using a method called "environmental sustainability index" (ESI). With this method, the following parameters are integrated into a predictive ecosystem health model: natural resource endowments, past and present pollution levels, environmental management efforts, contributions to protection of the global commons, and a society's capacity to improve its environmental performance over time. The ESI model predicts the health of an ecosystem on a fine-grained regional scale. In below example, the model calculations have been performed for the Netherlands.

The color-scale on the right side of the image depicts the normalized ESI index values that result from model calculations: the lowest value (at the bottom of the scale) indicates that the ecosystem has basically collapsed, such that sustainable human life is impossible. The highest value (at the top of the scale) indicates an optimal, healthy ecosystem state. Some parts in the modeled region (e.g., city centers, sand dunes, etc.) naturally have lower ESI index values.

The question: *A researcher presents you with the image below. The researcher claims that the depicted region shows a normal healthy ecosystem state, i.e., a country without any ecological problems that may disrupt human life.*

Using the response scale below the image, please rate how much you support the researcher's claim based on the image that shows the ecological state. If you tick the box on the far left side, you do not believe that the image supports the claim at all.

If you tick the box on the far right side, you believe that the image fully supports the claim. Tick the box that you believe represents the level of support for the made claim best.

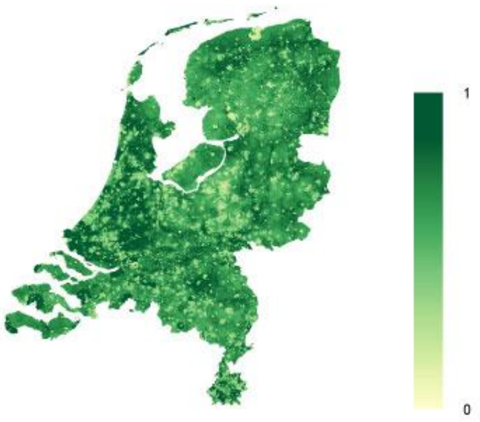

The image supports this claim not at all ○ ○ ○ ○ ○ ○ ○ ○ The image supports this claim very much

Evaluating geoimages

Please read the following text carefully before responding.

The color-scale on the right side of the image depicts the normalized ESI index values that result from model calculations: the lowest value (at the bottom of the scale) indicates that the ecosystem has basically collapsed, such that sustainable human life is impossible. The highest value (at the top of the scale) indicates an optimal, healthy ecosystem state. Some parts in the modeled region (e.g., city centers, sand dunes, etc.) naturally have lower ESI index values.

The question: *A researcher presents you with the image below. The researcher claims that the depicted region shows a collapsed ecosystem, i.e., a country that is ecologically dead. Should a country reach this stage, human life would not be possible anymore.*

Using the response scale below the image, please rate how much you support the researcher's claim based on the image that shows the ecological state. If you tick the box on the far left side, you do not believe that the image supports the claim at all.

If you tick the box on the far right side, you believe that the image fully supports the claim. Tick the box that you believe represents the level of support for the made claim best.

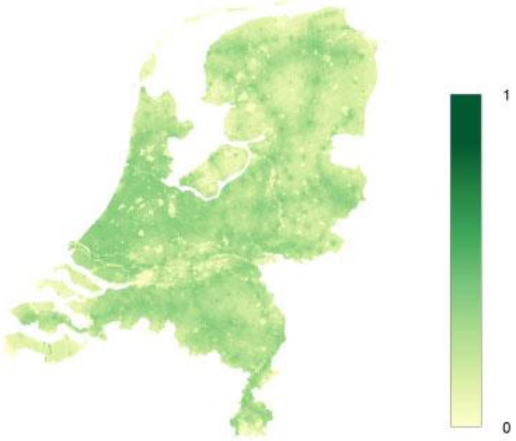

The image supports this claim not at all ○ ○ ○ ○ ○ ○ ○ ○ The image supports this claim very much

Evaluating geoimages

Please read the following text carefully before responding.

In the beginning, you have seen two ESI-images that show two extreme states with respect to a country's ecosystem health: The first image depicted the Netherlands in optimal ecological health state; the second image showed the Netherlands as an ecologically dead country. We now present you with an image depicting a scenario for the Netherlands with an ecological problem.

The question: *A researcher presents you with the image below. The researcher claims that the depicted scenario is in a so-called tipping-point state, i.e., the region has a more or less normal ecosystem health state, but appearing ecological problems in some areas may trigger a downward-trend that would be hard to reverse - nevertheless, recovery is still likely, if the right measures are taken.*

Using the response scale below indicate the country's ecological health state. The response scale shows on the far left that the ecosystem has basically collapsed, such that sustainable human life is impossible (i.e., "worst state"), and on the far right side an optimal, healthy ecosystem ("best state").

If you tick the box on the far left side, you believe that the image shows ecological death (no human life is possible) in the region, if you tick the box on the far right side, you believe that the image shows a fully functional ecosystem (normal ecosystem state). Tick the box you think best shows the country's ecosystem state.

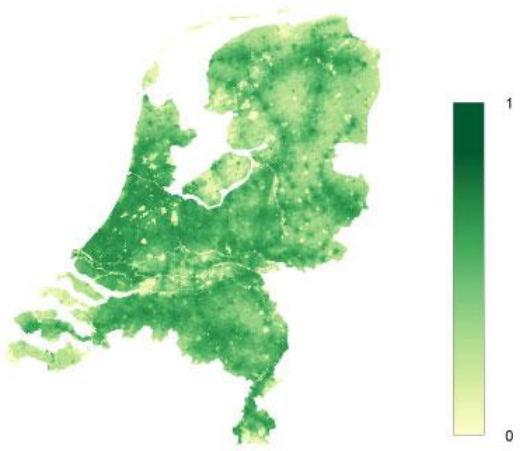

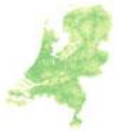

"worst state"

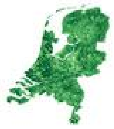

"best state"

Evaluating geoimages

Please read the following text carefully before responding.

In the beginning, you have seen two ESI-images that show two extreme states with respect to a country's ecosystem health: The first image depicted the Netherlands in optimal ecological health state; the second image showed the Netherlands as an ecologically dead country. We now present you with an image depicting a scenario for the Netherlands with an ecological problem.

The question: *A researcher presents you with the image below. The researcher claims that the depicted scenario shows the region in a minimal ecosystem health state, where large parts of the region display unfavorable ESI values. In such an ecosystem health state, there is a substantial risk that the region becomes unlivable unless radical measures are taken.*

Using the response scale below indicate the country's ecological health state. The response scale shows on the far left that the ecosystem has basically collapsed, such that sustainable human life is impossible (i.e., "worst state"), and on the far right side an optimal, healthy ecosystem ("best state").

If you tick the box on the far left side, you believe that the image shows ecological death (no human life is possible) in the region, if you tick the box on the far right side, you believe that the image shows a fully functional ecosystem (normal ecosystem state). Tick the box you think best shows the country's ecosystem state.

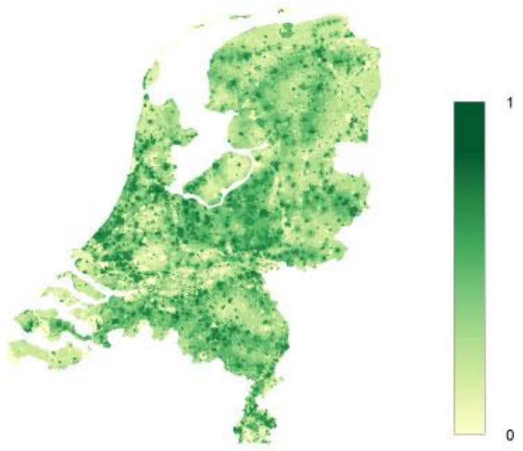

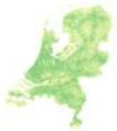

"worst state"

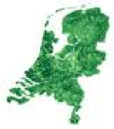

"best state"

Evaluating geoimages

Please read the following text carefully before responding.

In the beginning, you have seen two ESI-images that show two extreme states with respect to a country's ecosystem health: The first image depicted the Netherlands in optimal ecological health state; the second image showed the Netherlands as an ecologically dead country. We now present you with an image depicting a scenario for the Netherlands with an ecological Problem.

The question: *A researcher presents you with the image below. The researcher claims that the depicted scenario shows the region in a so-called beyond recovery state, where most parts of the region display unfavorable ESI values. Human existence is still possible but at bare minimum levels.*

Using the response scale below indicate the country's ecological health state. The response scale shows on the far left that the ecosystem has basically collapsed, such that sustainable human life is impossible (i.e., "worst state"), and on the far right side an optimal, healthy ecosystem ("best state").

If you tick the box on the far left side, you believe that the image shows ecological death (no human life is possible) in the region, if you tick the box on the far right side, you believe that the image shows a fully functional ecosystem (normal ecosystem state). Tick the box you think best shows the country's ecosystem state.

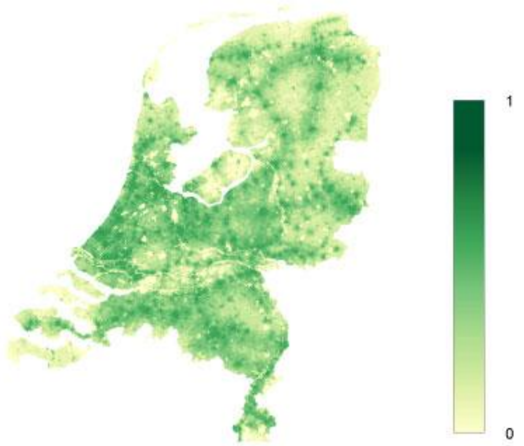

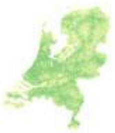

"worst state"

○ ○ ○ ○ ○ ○ ○ ○

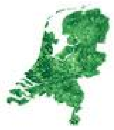

"best state"

Exp Geo red-yellow black

Evaluating geoimages

For the next five questions, we ask you to evaluate images that have been produced using techniques to measure the impact of human activity on the ecosystem in a given country. But before that, we would like to know your opinion on the following matter:

"The climate change we experience now is caused by human activities."

Please indicate to what extent you agree with this position:

- ○ I completely agree; human activites (burning fossil fuels, etc.) cause climate change
- ○ I somewhat agree; human activities contribute partly to climate change
- ○ I somewhat disagree; human activities only marginally contribute to climate change
- ○ I completely disagree; human activities do not contribute to climate change
- ○ I have no opinion on this matter

Evaluating geoimages

Please read the following text carefully before responding.

Below you see an image that has been produced using a method called "environmental sustainability index" (ESI). With this method, the following parameters are integrated into a predictive ecosystem health model: natural resource endowments, past and present pollution levels, environmental management efforts, contributions to protection of the global commons, and a society's capacity to improve its environmental performance over time. The ESI model predicts the health of an ecosystem on a fine-grained regional scale. In below example, the model calculations have been performed for the Netherlands.

The color-scale on the right side of the image depicts the normalized ESI index values that result from model calculations: the lowest value (at the bottom of the scale) indicates that the ecosystem has basically collapsed, such that sustainable human life is impossible. The highest value (at the top of the scale) indicates an optimal, healthy ecosystem state. Some parts in the modeled region (e.g., city centers, sand dunes, etc.) naturally have lower ESI index values.

The question: *A researcher presents you with the image below. The researcher claims that the depicted region shows a normal healthy ecosystem state, i.e., a country without any ecological problems that may disrupt human life.*

Using the response scale below the image, please rate how much you support the researcher's claim based on the image that shows the ecological state. If you tick the box on the far left side, you do not believe that the image supports the claim at all.

If you tick the box on the far right side, you believe that the image fully supports the claim. Tick the box that you believe represents the level of support for the made claim best.

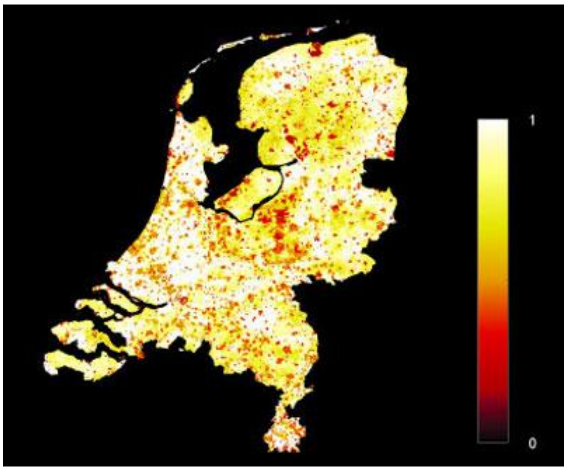

The image supports this claim not at all ○ ○ ○ ○ ○ ○ ○ ○ The image supports this claim very much

Evaluating geoimages

Please read the following text carefully before responding.

The color-scale on the right side of the image depicts the normalized ESI index values that result from model calculations: the lowest value (at the bottom of the scale) indicates that the ecosystem has basically collapsed, such that sustainable human life is impossible. The highest value (at the top of the scale) indicates an optimal, healthy ecosystem state. Some parts in the modeled region (e.g., city centers, sand dunes, etc.) naturally have lower ESI index values.

The question: *A researcher presents you with the image below. The researcher claims that the depicted region shows a collapsed ecosystem, i.e., a country that is ecologically dead. Should a country reach this stage, human life would not be possible anymore.*

Using the response scale below the image, please rate how much you support the researcher's claim based on the image that shows the ecological state. If you tick the box on the far left side, you do not believe that the image supports the claim at all.

If you tick the box on the far right side, you believe that the image fully supports the claim. Tick the box that you believe represents the level of support for the made claim best.

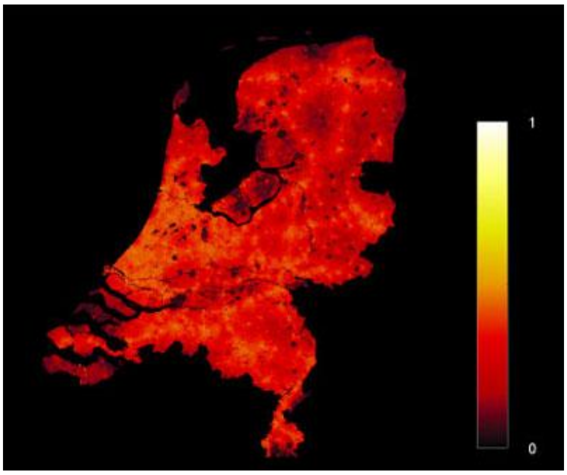

The image supports this claim not at all ○ ○ ○ ○ ○ ○ ○ ○ The image supports this claim very much

Evaluating geoimages

Please read the following text carefully before responding.

In the beginning, you have seen two ESI-images that show two extreme states with respect to a country's ecosystem health: The first image depicted the Netherlands in optimal ecological health state; the second image showed the Netherlands as

an ecologically dead country. We now present you with an image depicting a scenario for the Netherlands with an ecological problem.

The question: *A researcher presents you with the image below. The researcher claims that the depicted scenario is in a so-called tipping-point state, i.e., the region has a more or less normal ecosystem health state, but appearing ecological problems in some areas may trigger a downward-trend that would be hard to reverse - nevertheless, recovery is still likely, if the right measures are taken.*

Using the response scale below indicate the country's ecological health state. The response scale shows on the far left that the ecosystem has basically collapsed, such that sustainable human life is impossible (i.e., "worst state"), and on the far right side an optimal, healthy ecosystem ("best state").

If you tick the box on the far left side, you believe that the image shows ecological death (no human life is possible) in the region, if you tick the box on the far right side, you believe that the image shows a fully functional ecosystem (normal ecosystem state). Tick the box you think best shows the country's ecosystem state.

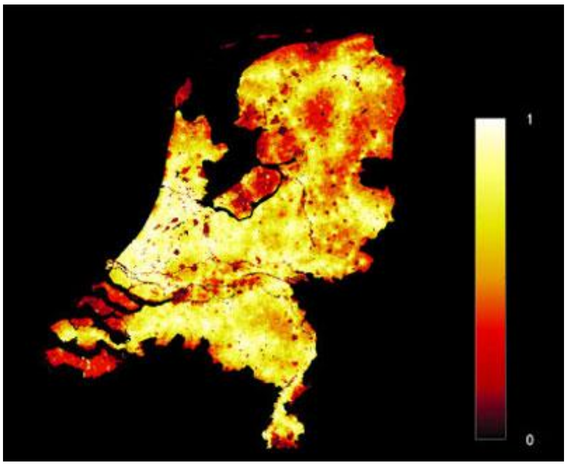

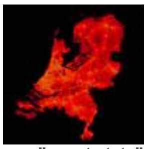

"worst state"

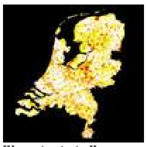

"best state"

Evaluating geoimages

Please read the following text carefully before responding.

In the beginning, you have seen two ESI-images that show two extreme states with respect to a country's ecosystem health: The first image depicted the Netherlands in optimal ecological health state; the second image showed the Netherlands as an ecologically dead country. We now present you with an image depicting a scenario for the Netherlands with an ecological problem.

The question: *A researcher presents you with the image below. The researcher claims that the depicted scenario shows the region in a minimal ecosystem health state, where large parts of the region display unfavorable ESI values. In such an ecosystem health state, there is a substantial risk that the region becomes unlivable unless radical measures are taken.*

Using the response scale below indicate the country's ecological health state. The response scale shows on the far left that the ecosystem has basically collapsed, such that sustainable human life is impossible (i.e., "worst state"), and on the far right side an optimal, healthy ecosystem ("best state").

If you tick the box on the far left side, you believe that the image shows ecological death (no human life is possible) in the region, if you tick the box on the far right side, you believe that the image shows a fully functional ecosystem (normal ecosystem state). Tick the box you think best shows the country's ecosystem state.

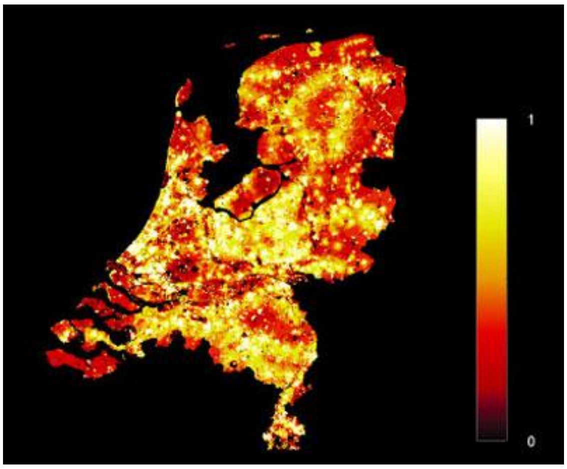

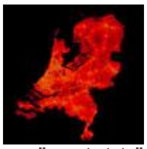

"worst state"

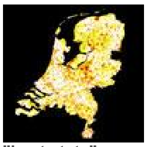

"best state"

Evaluating geoimages

Please read the following text carefully before responding.

In the beginning, you have seen two ESI-images that show two extreme states with respect to a country's ecosystem health: The first image depicted the Netherlands in optimal ecological health state; the second image showed the Netherlands as an ecologically dead country. We now present you with an image depicting a scenario for the Netherlands with an ecological Problem.

The question: *A researcher presents you with the image below. The researcher claims that the depicted scenario shows the region in a so-called beyond recovery state, where most parts of the region display unfavorable ESI values. Human existence is still possible but at bare minimum levels.*

Using the response scale below indicate the country's ecological health state. The response scale shows on the far left that the ecosystem has basically collapsed, such that sustainable human life is impossible (i.e., "worst state"), and on the far right side an optimal, healthy ecosystem ("best state").

If you tick the box on the far left side, you believe that the image shows ecological death (no human life is possible) in the region, if you tick the box on the far right side, you believe that the image shows a fully functional ecosystem (normal ecosystem state). Tick the box you think best shows the country's ecosystem state.

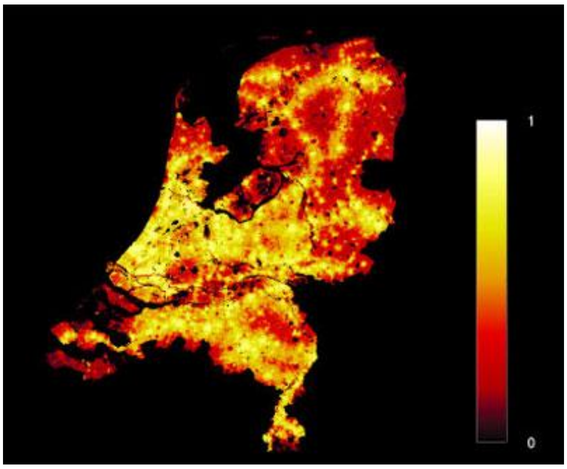

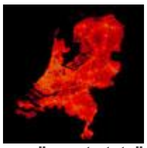

"worst state"

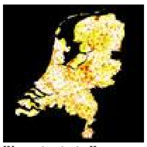

"best state"

Exp Geo red-yellow white

Evaluating geoimages

For the next five questions, we ask you to evaluate images that have been produced using techniques to measure the impact of human activity on the ecosystem in a given country. But before that, we would like to know your opinion on the following matter:

"The climate change we experience now is caused by human activities."

Please indicate to what extent you agree with this position:

- I completely agree; human activites (burning fossil fuels, etc.) cause climate change
- I somewhat agree; human activities contribute partly to climate change
- I somewhat disagree; human activities only marginally contribute to climate change
- I completely disagree; human activities do not contribute to climate change
- I have no opinion on this matter

Evaluating geoimages

Please read the following text carefully before responding.

Below you see an image that has been produced using a method called "environmental sustainability index" (ESI). With this

method, the following parameters are integrated into a predictive ecosystem health model: natural resource endowments, past and present pollution levels, environmental management efforts, contributions to protection of the global commons, and a society's capacity to improve its environmental performance over time. The ESI model predicts the health of an ecosystem on a fine-grained regional scale. In below example, the model calculations have been performed for the Netherlands.

The color-scale on the right side of the image depicts the normalized ESI index values that result from model calculations: the lowest value (at the bottom of the scale) indicates that the ecosystem has basically collapsed, such that sustainable human life is impossible. The highest value (at the top of the scale) indicates an optimal, healthy ecosystem state. Some parts in the modeled region (e.g., city centers, sand dunes, etc.) naturally have lower ESI index values.

The question: *A researcher presents you with the image below. The researcher claims that the depicted region shows a normal healthy ecosystem state, i.e., a country without any ecological problems that may disrupt human life.*

Using the response scale below the image, please rate how much you support the researcher’s claim based on the image that shows the ecological state. If you tick the box on the far left side, you do not believe that the image supports the claim at all.

If you tick the box on the far right side, you believe that the image fully supports the claim. Tick the box that you believe represents the level of support for the made claim best.

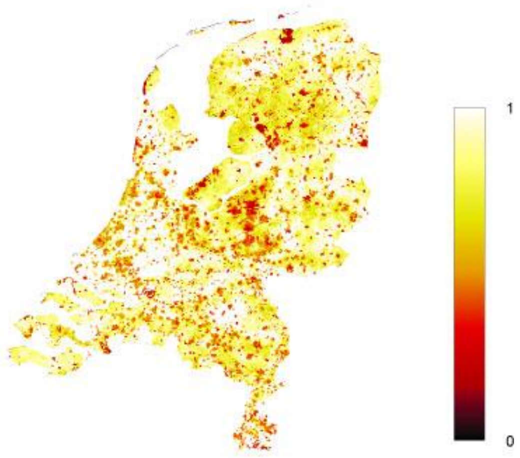

The image supports this claim not at all ◯ ◯ ◯ ◯ ◯ ◯ ◯ ◯ The image supports this claim very much

Evaluating geoimages

Please read the following text carefully before responding.

The color-scale on the right side of the image depicts the normalized ESI index values that result from model calculations: the lowest value (at the bottom of the scale) indicates that the ecosystem has basically collapsed, such that sustainable human life is impossible. The highest value (at the top of the scale) indicates an optimal, healthy ecosystem state. Some parts in the modeled region (e.g., city centers, sand dunes, etc.) naturally have lower ESI index values.

The question: *A researcher presents you with the image below. The researcher claims that the depicted region shows a collapsed ecosystem, i.e., a country that is ecologically dead. Should a country reach this stage, human life would not be possible anymore.*

Using the response scale below the image, please rate how much you support the researcher’s claim based on the image that shows the ecological state. If you tick the box on the far left side, you do not believe that the image supports the claim at all.

If you tick the box on the far right side, you believe that the image fully supports the claim. Tick the box that you believe represents the level of support for the made claim best.

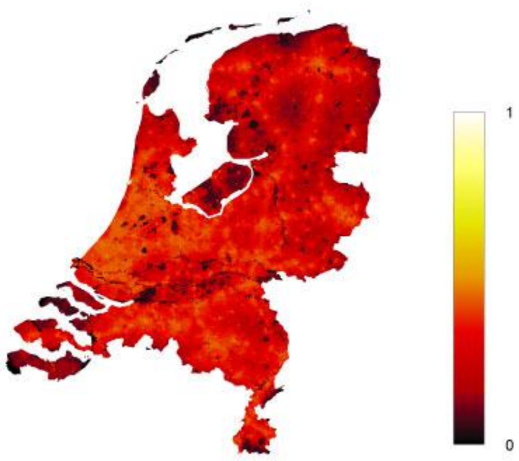

The image supports this claim not at all ◯ ◯ ◯ ◯ ◯ ◯ ◯ ◯ The image supports this claim very much

Evaluating geoimages

Please read the following text carefully before responding.

In the beginning, you have seen two ESI-images that show two extreme states with respect to a country's ecosystem health: The first image depicted the Netherlands in optimal ecological health state; the second image showed the Netherlands as an ecologically dead country. We now present you with an image depicting a scenario for the Netherlands with an ecological problem.

The question: *A researcher presents you with the image below. The researcher claims that the depicted scenario is in a so-called tipping-point state, i.e., the region has a more or less normal ecosystem health state, but appearing ecological problems in some areas may trigger a downward-trend that would be hard to reverse - nevertheless, recovery is still likely, if the right measures are taken.*

Using the response scale below indicate the country's ecological health state. The response scale shows on the far left that the ecosystem has basically collapsed, such that sustainable human life is impossible (i.e., "worst state"), and on the far right side an optimal, healthy ecosystem ("best state").

If you tick the box on the far left side, you believe that the image shows ecological death (no human life is possible) in the region, if you tick the box on the far right side, you believe that the image shows a fully functional ecosystem (normal ecosystem state). Tick the box you think best shows the country's ecosystem state.

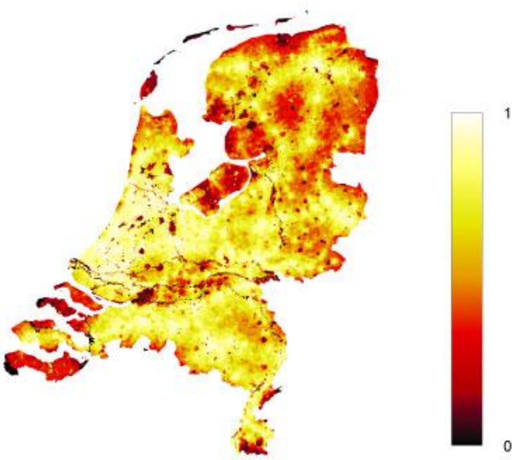

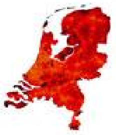

"worst state"

○ ○ ○ ○ ○ ○ ○ ○

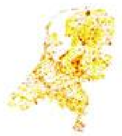

"best state"

Evaluating geoimages

Please read the following text carefully before responding.

In the beginning, you have seen two ESI-images that show two extreme states with respect to a country's ecosystem health: The first image depicted the Netherlands in optimal ecological health state; the second image showed the Netherlands as an ecologically dead country. We now present you with an image depicting a scenario for the Netherlands with an ecological problem.

The question: *A researcher presents you with the image below. The researcher claims that the depicted scenario shows the region in a minimal ecosystem health state, where large parts of the region display unfavorable ESI values. In such an ecosystem health state, there is a substantial risk that the region becomes unlivable unless radical measures are taken.*

Using the response scale below indicate the country's ecological health state. The response scale shows on the far left that the ecosystem has basically collapsed, such that sustainable human life is impossible (i.e., "worst state"), and on the far right side an optimal, healthy ecosystem ("best state").

If you tick the box on the far left side, you believe that the image shows ecological death (no human life is possible) in the region, if you tick the box on the far right side, you believe that the image shows a fully functional ecosystem (normal ecosystem state). Tick the box you think best shows the country's ecosystem state.

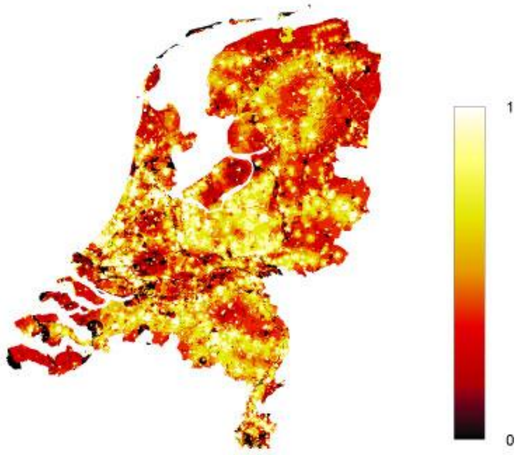

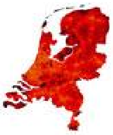

"worst state"

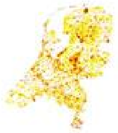

"best state"

Evaluating geoimages

Please read the following text carefully before responding.

In the beginning, you have seen two ESI-images that show two extreme states with respect to a country's ecosystem health: The first image depicted the Netherlands in optimal ecological health state; the second image showed the Netherlands as an ecologically dead country. We now present you with an image depicting a scenario for the Netherlands with an ecological Problem.

The question: *A researcher presents you with the image below. The researcher claims that the depicted scenario shows the region in a so-called beyond recovery state, where most parts of the region display unfavorable ESI values. Human existence is still possible but at bare minimum levels.*

Using the response scale below indicate the country's ecological health state. The response scale shows on the far left that the ecosystem has basically collapsed, such that sustainable human life is impossible (i.e., "worst state"), and on the far right side an optimal, healthy ecosystem ("best state").

If you tick the box on the far left side, you believe that the image shows ecological death (no human life is possible) in the region, if you tick the box on the far right side, you believe that the image shows a fully functional ecosystem (normal ecosystem state). Tick the box you think best shows the country's ecosystem state.

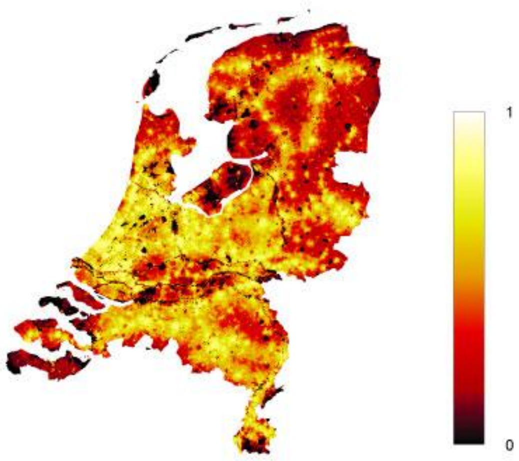

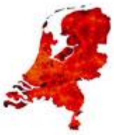

"worst state"

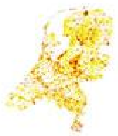

"best state"

Neuroscience 1

Your training

Please indicate the main field (one choice) that best describes your highest level of training (e.g., graduate or postgraduate studies)

I have training in

Your current occupation

Please indicate the main field (one choice) that best describes your current occupation:

I am currently occupied in

Please comment below if none of the choices given describe your training and/or occupation.

Your general relationship with neuroimaging

In the following, the term "neuroimaging" refers to any methodology that aims to measure brain structures or functions in any setting (clinical, basic research, etc.).

How many years of experience (both as a producer or consumer of brain data images) do you personally have in neuroimaging:

Which statement better describes your general relationship towards neuroimaging?

- ◯ I am predominantly a "consumer" of neuroimages (e.g., I use them when diagnosing patients or I read neuroimaging publications).
- ◯ Besides being a "consumer", I'm also a "producer" of neuroimaging data / images (i.e., I'm actively involved in the generation of neuroimages).

Your professional relationship to neuroimaging

Please indicate your current involvement in the following fields of practice:

	Never	Sometimes	Often	Main field
Generation of neuroimages for clinical settings	◯	◯	◯	◯
Design of neuroimaging experiments (research)	◯	◯	◯	◯
Execution of neuroimaging experiments (research)	◯	◯	◯	◯
Neuroimaging data analysis & visualization (clinic or research)	◯	◯	◯	◯
Computational neuroscience aspects (e.g., statistical method development)	◯	◯	◯	◯
Development of neuroimaging equipment (hardware)	◯	◯	◯	◯
Maintenance of neuroimaging equipment	◯	◯	◯	◯
Neuroimaging software development	◯	◯	◯	◯

Please comment below if you are involved in additional fields of practice related to neuroimaging not listed above.

Your institutional setting

In the following, the term "lab" refers to the organizational unit in which you are embedded and that consists of co-workers with which you collaborate routinely in your professional work (e.g., research group, small institute, clinic, etc.).

How many people work in your lab?

◯

Less than 5

◯ 5-10

◯ 11-20

◯ more than 20

◯ I'm not associated to a lab

For how many years (approximately) has your lab been active in neuroimaging research (either performing neuroimaging or collaborating with people doing neuroimaging)?

[ ]

Does your lab produce neuroimage data (using technologies like Computer Tomography, EEG, MRI, PET etc.)

◯ Yes

◯ No

Use of color in neuroimages

These questions only refer to functional neuroimages, i.e., images that display e.g. electrical activity, BOLD-signals, receptor density measured, e.g., by fMRI, PET or EEG.

Please indicate, according to your personal opinion, which of the following scales should be used to display the increase of a statistical parameter of any type (e.g., activity, receptor density etc.).

	Not suitable	Moderately suitable	Suitable	Highly suitable
**Scale A)** 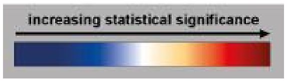	◯	◯	◯	◯
**Scale B)** 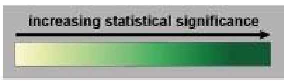	◯	◯	◯	◯
**Scale C)** 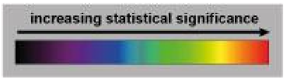	◯	◯	◯	◯
**Scale D)** 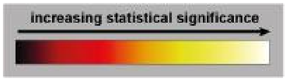	◯	◯	◯	◯

Do you believe that only one type of color scale should be used when displaying the increase of a statistical parameter in neuroimaging?

◯ Yes, the following color-scale (A, B, C or D) should be used as a standard

[ ]

◯ Yes, but another color scale should be used as a standard (please describe briefly the scale below, e.g., indicate which colors should be used)

[ ]

◯ No standard should be used (please comment briefly why)

[ ]

When producing a functional neuroimage (if you don't produce neuroimages, see below): What guidelines do you follow when choosing a color scale (e.g., like the ones shown above) displaying t-values (or other statistical parameters) of brain activation?

	Never	Occasionally	Frequently	Mostly
The analysis software I use guides my choice.	◯	◯	◯	◯
The technical literature guides my choice.	◯	◯	◯	◯
The lab-internal rules guide my choice.	◯	◯	◯	◯
The publication format guides my choice.	◯	◯	◯	◯
My intuition guides my choice.	◯	◯	◯	◯

Tick the box below only if you don't produce functional neuroimages and you therefore cannot answer this question.

◯ I do not produce functional neuroimages

Neuroscience 2

Your involvement in neuroimaging

In the following, the term "neuroimaging experiment" refers to the creation of any kind of image of the brain (structural, functional) for any purpose (clinical, research).

How many research participants volunteer for neuroimaging experiments at your lab and/or with how many patients do you carry out neuroimaging studies?

- ◯ None
- ◯ Less than 50 per year
- ◯ 51 - 100 per year
- ◯ More than 100 per year

Please indicate the balance between the number of imaging experiments made for clinical purposes or for basic research purposes (e.g. cognitive neuroscience) in your lab:

Only Imaging for clinical purposes ◯ ◯ ◯ ◯ ◯ Only Imaging for basic research

Neuroimaging techniques

What kinds of neuroimaging techniques have been used in your lab during the past 5 years?

	Never	Occasionally	Frequently	As a standard method
Computer Tomography	◯	◯	◯	◯
EEG (all sub-types)	◯	◯	◯	◯
MEG	◯	◯	◯	◯
MRI morphometry	◯	◯	◯	◯
other structural MRI	◯	◯	◯	◯
Diffusion Tensor Imaging	◯	◯	◯	◯
functional MRI (all types)	◯	◯	◯	◯
PET	◯	◯	◯	◯
SPECT	◯	◯	◯	◯
Optical Imaging	◯	◯	◯	◯

Please comment below if your lab uses other neuroimaging techniques not listed above.

Neuroimaging data processing

Which software (any version type) have you been using for neuroimaging data processing and display in the last 5 years?

	Never	Occasionally	Frequently	As a standard method
AFNI	◯	◯	◯	◯
Brain Voyager	◯	◯	◯	◯
fMRIstat	◯	◯	◯	◯
FSL	◯	◯	◯	◯
Lipsia	◯	◯	◯	◯
MedX	◯	◯	◯	◯
SPM	◯	◯	◯	◯
Stimulate	◯	◯	◯	◯
VoxBo	◯	◯	◯	◯

Please comment below if your lab uses other neuroimaging data processing tools not listed above.

When generating an image using software mentioned above: how often do you explore the possibilities of the neuroimaging data processing software (e.g., changing significance levels) purely for graphical purposes (e.g., to optimize the appearance of the image with respect to the "message" the image should convey to the observer)?

- ◯ Never
- ◯ Occasionally
- ◯ Frequently
- ◯ In almost every case

Please add any comment to this question, if necessary.

Purpose of neuroimages

Neuroimages can be produced for various purposes. Please give a rough estimate of the fraction (in percent) of all images that have been produced in your lab during the past 5 years for each of the following possibilities (the percentages have to sum up to 100%; if images fall in more than one purpose, choose the one that has the highest dissemination. E.g., an image that first has been shown at a conference but later is included in a peer-reviewed publication will be classified as "peer-reviewed publication"):

- 0 % images purely for internal use within the lab (e.g., in the context of a research project)
- 0 % images for a clinical purpose that are discussed with colleagues and/or patients
- 0 % images that are used for scientific talks or conference posters
- 0 % images for peer-reviewed publications
- 0 % images that reach a broad audience (e.g., for press releases, newspaper articles etc.)

Post-processing of neuroimages

Graphical post-processing of raw images obtained by the neuroimaging analysis software can mean different things. Please mark the frequency for doing the following types of post-processing when preparing images for publications of any type:

	Never	Occasionally	Frequently	Routinely
We arrange single raw figures to more complex images that may also include other types of images (e.g. graphical charts)	◯	◯	◯	◯
We add arrows or other markers to pinpoint specific aspects on the image	◯	◯	◯	◯
We change the original gray-scales or color-codes that emerged from the data processing software	◯	◯	◯	◯
We change the background of the image (e.g., from black to white)	◯	◯	◯	◯
We modify (delete or emphasize) parts of the pictures that represent data (e.g., by deleting areas of activations that are unrelated to the purpose of the study)	◯	◯	◯	◯

Please comment, if the amount of post-processing strongly depends on the purpose of the neuroimage (e.g., images for peer-reviewed journals versus images for informal talks or general public). You may also add additional types of post-processing you do that are not listed above.

Please indicate how frequently you use one of the following graphics processing software for image post-processing:

	Never	Occasionally	Frequently	As a standard method
Adobe Photoshop	◯	◯	◯	◯
Adobe Illustrator	◯	◯	◯	◯
Matlab	◯	◯	◯	◯
CorelDraw	◯	◯	◯	◯
GIMP (Gnu Image Manipulation Program)	◯	◯	◯	◯

Please comment, if you use other types of post-processing software not listed above.

Geo general

Your expertise in geographic information visualization

Below are two examples of images produced by mapping/GIS software. The first displays the annual mean surface temperature, the second image displays the global vegetation density.

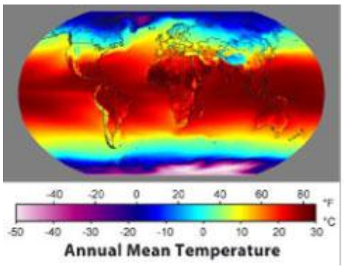

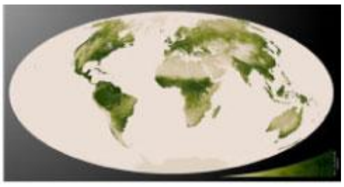

Do you have any specific relation to the production of such maps beside seeing them sometimes in the lay media? Please check all that apply:

- ☐ I have produced such types of maps
- ☐ I collaborate with experts/institutions that produce such types of maps
- ☐ Such maps are an inspiration for me when displaying data in my own field
- ☐ I regularly see such maps (e.g., in scientific journals, lay media)
- ☐ I have a basic understanding of how geographic Information visualization works

Geoscience 1

Your training

Please indicate the main field (one choice) that best describes your highest level of training (e.g., graduate or postgraduate studies):

I have training in

Your current occupation

Please indicate the main field (one choice) that best describes your current occupation:

I am currently occupied in

Please comment below if none of the choices given describe your training and/or occupation.

Your general relationship with geographic information visualization

In the following, the term "geographic information visualization" refers to any methodology or techniques supporting geospatial data analysis through the use of visualization in any setting (basic research, applied, etc.).

The term "maps" refers to displays that have been produced by such tools and techniques.

The term "geographic information system" (GIS) is a system designed to capture, store, manage, manipulate, analyze, and depict all types of geographical data.

How many years of experience do you personally have in geographic information visualization (both as producer or consumer of maps)?

Which statement better describes your general relationship towards geographic information visualization?

- ○ I am predominantly a “consumer” of maps (map user).
- ○ I am also a “producer” of maps (cartographer/GIS specialist).

Your professional relationship to geographic information visualization

Please indicate your current involvement in the following fields of practice:

	Never	Sometimes	Often	Main field
Design of map/GIS displays	○	○	○	○
Production of map/GIS displays	○	○	○	○
Geographic data analysis & visualization	○	○	○	○
Computational geographic data aspects (spatial analysis/geocomputation).	○	○	○	○
Cartography/GIS/spatial analysis software development	○	○	○	○

Please comment below if you are involved in additional fields of practice related to geographic information visualization not listed above.

Your institutional setting

In the following, the term “lab” refers to the organizational unit in which you are embedded and that consists of co-workers with which you collaborate routinely in your professional work (e.g., research group, small institute, map production site, etc.).

How many people work in your lab?

- ○ Less than 5
- ○ 5-10
- ○ 11-20
- ○ more than 20
- ○ I'm not associated to a lab

For how many years (approximately) has your lab been active in geographic information visualization (either creating map displays or collaborating with cartography/GIS labs)?

Does your lab produce map/GIS displays of any kind?

- ○ Yes
- ○ No

Use of color in maps

These questions only refer to maps that display continuous statistical information, e.g., temperatures, albedo, or population density.

Please indicate, according to your personal opinion, which of the following scales should be used to display the increase of a statistical parameter of any type (e.g., temperature, albedo etc.).

	Not suitable	Moderately suitable	Suitable	Highly suitable
Scale A) 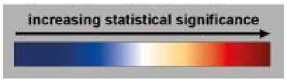	○	○	○	○
Scale B) 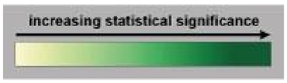	○	○	○	○

	Not suitable	Moderately suitable	Suitable	Highly suitable
Scale C) 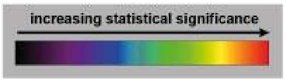	○	○	○	○
Scale D) 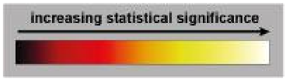	○	○	○	○

Do you believe that only one type of color scale should be used when displaying the increase of a statistical parameter?

○ Yes, the following color-scale (A, B, C or D) should be used as a standard

○ Yes, but another color scale should be used as a standard (please describe briefly the scale below)

○ No standard should be used (please comment briefly why)

When producing a map (if you don't produce maps, see below): What guidelines do you follow when choosing a color scale (e.g., like the ones shown above) displaying data?

	Never	Occasionally	Frequently	Mostly
The mapping/GIS software I use guides my choice.	○	○	○	○
The technical literature guides my choice.	○	○	○	○
The lab-internal rules guide my choice.	○	○	○	○
The publication format guides my choice.	○	○	○	○
My intuition guides my choice.	○	○	○	○

Tick the box below only if you don't produce maps and you therefore cannot answer this question.

○ I do not produce maps

Geoscience 2

Your involvement in geographic information visualization

How many maps/GIS displays per year are produced in your lab?

○ None

○ Less than 50 per year

○ 51 - 100 per year

○ More than 100 per year

Please indicate the balance between the number of maps made for public dissemination/production purposes or for research purposes (e.g., empirical cartography studies):

Only mapping for production purposes | ○ ○ ○ ○ ○ | Only mapping for research

Geographic information visualization techniques

What kinds of mapping techniques have been used in your lab during the past 5 years?

	Never	Occasionally	Frequently	As a standard method
Topographic maps (print)	○	○	○	○
Topographic maps (digital/online)	○	○	○	○
Reference maps (print)	○	○	○	○
Reference maps (digital/online)	○	○	○	○
Thematic maps (print)	○	○	○	○
Thematic maps (digital/online)	○	○	○	○
Navigational charts (print)	○	○	○	○
Navigational charts (digital/online)	○	○	○	○
Image maps /satellite images (print)	○	○	○	○
Image maps /satellite images (digital/online)	○	○	○	○

Please comment below if your lab uses other mapping techniques not listed above.

Geography information visualization data processing

Which software (any version type) have you been using for map/GIS data processing and display in the last 5 years?

	Never	Occasionally	Frequently	As a standard method
Adobe Illustrator	◯	◯	◯	◯
Adobe Photoshop	◯	◯	◯	◯
ESRI ArcGIS	◯	◯	◯	◯
GeoMedia Intergraph	◯	◯	◯	◯
QGIS	◯	◯	◯	◯
OpenStreeetMap	◯	◯	◯	◯
GoogleMap/Earth	◯	◯	◯	◯

Please comment below if your lab uses other map/GIS data processing tools not listed above.

When generating a map display using software mentioned above: how often do you explore the possibilities of data processing with the software (e.g., changing the data classification or color scheme) purely for graphical purposes (e.g., to optimize the appearance of the image with respect to the "message" the image should convey to the observer)?

◯ Never

◯ Occasionally

◯ Frequently

◯ In almost every case

Please add any comment to this question, if necessary.

Purpose of maps

Maps can be produced for various purposes. Please give a rough estimate of the fraction (in percent) of all maps that have been produced in your lab during the past 5 years for each of the following possibilities (the percentages have to sum up to 100%; if maps fall in more than one purpose, choose the one that has the highest dissemination. E.g., a map that first has been shown at a conference but later is included in a peer-reviewed publication will be classified as "peer-reviewed publication"):

0 % maps purely for internal use within the lab (e.g., in the context of a research project)

0 % maps that have been generated in a contractual relation with third parties

0 % maps that are used for scientific talks or conference posters

0 % maps for peer-reviewed publications

0 % maps that reach a broad audience (e.g., for press releases, newspaper articles etc.)

Post-processing of maps

Graphical post-processing of raw images obtained by mapping/GIS software can mean different things. Please mark the frequency for doing the following types of post-processing when preparing maps for publications of any type:

	Never	Occasionally	Frequently	Routinely
We arrange single raw maps to more complex map displays that may also include other types of images (e.g., graphical charts)	◯	◯	◯	◯

	Never	Occasionally	Frequently	Routinely
We add arrows or other markers to pinpoint specific aspects on the map	○	○	○	○
We change the original gray-scales or color-codes that emerged from prior data processing software	○	○	○	○
We change the background of the display (e.g., from black to white)	○	○	○	○
We modify (delete or emphasize) those parts of the display that represent data (e.g., by removing areas on the map that do not have any data values, and/or are unrelated to the purpose of the study)	○	○	○	○

Please comment, if the amount of post-processing strongly depends on the purpose of the map (e.g., images for peer-reviewed journals versus images for informal talks or general public). You may also add additional types of post-processing you do that are not listed above.

Please indicate how frequently you use one of the following graphics processing software for image post-processing:

	Never	Occasionally	Frequently	As a standard method
Adobe Photoshop	○	○	○	○
Adobe Illustrator	○	○	○	○
Matlab	○	○	○	○
CorelDraw	○	○	○	○
GIMP (Gnu Image Manipulation Tool)	○	○	○	○

Please comment, if you use other types of post-processing software not listed above.

Neuro general

Your expertise in neuroimaging

Below are two examples of images produced by neuroimaging software. The first displays statistically significant activation of a brain region related to a specific task measured by fMRI, the second image displays a brain tumor measured by computer tomography.

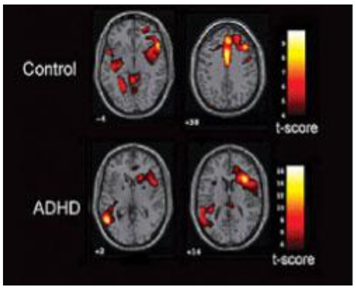

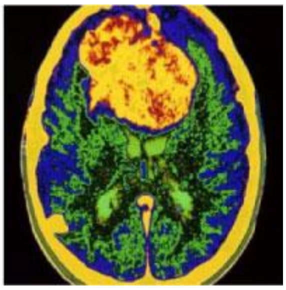

Do you have any relation to the production of such neuroimages beside seeing them sometimes in the lay media? Please check all that apply:

- ☐ I have produced such types of images
- ☐ I collaborate with experts/institutions that produce such types of images
- ☐ Such images are an inspiration for me when displaying data in my own field
- ☐ I regularly see such images (e.g., in scientific journals, lay media)
- ☐ I have a basic understanding of how methods like MRI, fMRI, PET etc. work
- ☐ I participated in neuroimaging experiments as research subject
- ☐ A clinical scan of the brain has been taken from me for medical purposes

Schluss

Final page

We thank you for your time to answer our survey.

Please send an E-mail to christen@ethik.uzh.ch, if you would like to obtain the results of our survey or if you have any additional questions or comments.

Please click on the button on the right side to transmit the survey data.
