# Supplementary material for: Susceptibility of domain experts to color manipulation indicate a need for design principles in data visualization
Source: PLoS One. 2021 Feb 4;16(2):e0246479. doi: 10.1371/journal.pone.0246479 (PMC7861358; doi:10.1371/journal.pone.0246479)
Supplement: S1 Table — (DOCX) [file pone.0246479.s002.docx]

**Markus Christen, Peter Brugger, Sara Irina Fabrikant: Susceptibility of domain experts to color manipulation indicate a need for design principles in data visualization**

**S1 Table.**

|  |  | **Data context** | |
| --- | --- | --- | --- |
|  | **Factor** | **neuro** | **geo** |
| **Experience** | Personal experience Neuro (n=132) | n.s. | n.s. |
|  | Lab experience Neuro (n=129) | n.s. | n.s. |
|  | Overall experience Neuro (n=129) | n.s. | n.s |
|  | Neuro experience in geo data context (n=129) | n.s. | n.s. |
|  | Personal experience Geo (n=133) | n.s. | n.s. |
|  | Lab experience Geo (n=131) | n.s. | n.s. |
|  | Overall experience Geo (n=131) | n.s. | n.s. |
|  | Geo experience in neuro data context (n=131) | n.s. | n.s. |
|  | Overall experience lay people | n.s. | n.s. |
| **Practices** | Software Neuro (n=116) | n.s. | n.s. |
|  | Technical literature Neuro (n=116) | n.s. | n.s. |
|  | Lab rules Neuro (n=113) | n.s. | n.s. |
|  | Publication format Neuro (n=114) | n.s. | n.s. |
|  | Intuition Neuro (n=116) | n.s. | -0.20* |
|  | Image manipulation index Neuro (n=92) | n.s. | n.s. |
|  | Image outreach index Neuro (n=97) | n.s. | n.s. |
|  | Software Geo (n=128) | n.s. | n.s. |
|  | Technical literature Geo (n=128) | n.s. | -0.16^(^*^)^ |
|  | Lab rules Geo (n=128) | n.s. | n.s. |
|  | Publication format Geo (n=128) | n.s. | n.s. |
|  | Intuition Geo (n=130) | n.s. | n.s. |
|  | Image manipulation index Geo (n=106) | n.s. | n.s. |
|  | Image outreach index Geo (n=111) | n.s. | n.s. |
| **Attitudes** | Standardization opinion Neuro^1^ | n.s. | n.s. |
|  | Suitability rainbow scale Neuro (n=129) | n.s. | n.s. |
|  | Suitability Blue-red-scale Neuro (n=129) | n.s. | n.s. |
|  | Suitability Green scale Neuro (n=129) | n.s. | n.s. |
|  | Suitability Heated-body-scale Neuro (n=129) | n.s. | n.s. |
|  | Brain death opinion Neuro (n=126) | n.s. | - |
|  | Climate change opinion Neuro (n=133) | - | 0.16^(^*^)^ |
|  | Standardization opinion Geo^1^ | n.s. | n.s. |
|  | Suitability rainbow scale Geo (n=132) | n.s. | n.s. |
|  | Suitability Blue-red-scale Geo (n=132) | n.s. | n.s. |
|  | Suitability Green scale Geo (n=132) | n.s. | n.s. |
|  | Suitability Heated-body-scale Geo (n=132) | n.s. | n.s. |
|  | Brain death opinion Geo (n=125) | n.s. | - |
|  | Climate change opinion Geo (n=132) | - | n.s. |
|  | Brain death opinion lay people (n=134) | n.s. | - |
|  | Climate change opinion lay people (n=133) | - | n.s. |

**S1 Table. Results for potential factors influencing trust in color scales.** The table displays Spearman's rank correlations of various factors determining experiences, image generation practices and attitudes with overall data interpretation variability for the neuroscience and the geographic data contexts. Almost none of those factors seem to influence interpretation variability. Significance level of difference between those for standard and those against standard: *: <.05; (*): <.1
