# Supplementary material for: Susceptibility of domain experts to color manipulation indicate a need for design principles in data visualization
Source: PLoS One. 2021 Feb 4;16(2):e0246479. doi: 10.1371/journal.pone.0246479 (PMC7861358; doi:10.1371/journal.pone.0246479)
Supplement: Attachment — Submitted filename: Response to Reviewers_final.docx [file pone.0246479.s003.docx]

**Susceptibility of domain experts to color manipulation indicate a need for design principles in data visualization – Response to Reviewers**

We thank the reviewers for their detailed and constructive review. Below we respond to revision suggestions by reviewers in turn. All respective changes in the revised manuscript are marked in yellow in the document “Revised Manuscript with Track Changes”. We did not highlight linguistic or grammatical corrections for better readability.

**Editorial remarks**

*Please ensure that your manuscript meets PLOS ONE's style requirements, including those for file naming.*

We have checked the requirements and made changes accordingly. In particular, we updated the reference style.

*Please include additional information regarding the survey or questionnaire used in the study and ensure that you have provided sufficient details that others could replicate the analyses. For instance, if you developed a questionnaire as part of this study and it is not under a copyright more restrictive than CC-BY, please include a copy, in both the original language and English, as Supporting Information.*

We made the questionnaire available as Supporting Information.

*We note that you have stated that you will provide repository information for your data at acceptance. Should your manuscript be accepted for publication, we will hold it until you provide the relevant accession numbers or DOIs necessary to access your data.*

We have uploaded the dataset on Zenodo; the DOI is: 10.5281/zenodo.4284817

**Reviewer 1**

*R1-1 While there are examples of how trust data collected, there is no description of how trust was defined to participants. Different conceptualizations of trust may influence how participants report trust. For example, how well do you trust yourself to use this visualization effectively seems like it may be more influenced by color choices than how well do you trust the data in this visualization to help you draw conclusions. While the latter framing is more ecologically valid (and seems closer to the likely framing based on evidence in the paper), it also leads to a bias whereby people may be willing to trust the visualization they’re using for a given task even though they know it is suboptimal (i.e., to report it as “good enough”). This is touched on in the discussion, but having more detail as to how trust was conceptualized (or interpreted) would significantly strengthen and contextualize the discussions of trust.*

We fully agree with this reviewer that the “trust” concept is complex and could involve various aspects such as trusting the data as such or trusting whether a certain visualization correctly translates the content of the data. We have clarified on page 4 that in our survey, the notion of trust concerns whether participants believe the data presented in a certain type of visualization supported an empirical claim by a domain expert. We refer to the exact wording of the trust-related questions on page 9. The original survey document is now added as supplementary information. We added a short discussion on the various ways how trust could be understood on page 22.

*R1-2 I appreciate the thoughtful considerations of CVDs. However, was any sort of CVD screening carried out? Many people do not know they have any sort of CVD until adulthood, making self-reporting challenging and at times inaccurate. This is a minor limitation as online CVD tests are imperfect due to a number of factors, like monitor calibration, but it is worth explicitly mentioning.*

On page 22, we added respective information that no CVD screening had been performed and why this is the case. We also further explain why we believe that the potential effect of this limitation is unlikely to have affected the main results of our study.

*R1-3 The manuscript notes that five common demographic questions were provided to all participants. However, no information about those five questions is provided. Reading the results section, it appears that these questions were used to draw conclusions about the data; however, having a summary of these questions would be helpful for understanding the study. For example, did any common questions assess lay participants’ comfort with data generally or experience with design?*

On page 10-11 we have added examples of the survey questions used to assess “general experience” and we refer to supplementary information that includes the entire questionnaire. These questions aim to assess experience with the design of such maps. We did not ask lay people about their general comfort with data. This has been added as a limitation on page 22. We thank the reviewer for helping us clarify this point.

*R1-4 For the statistical reporting, it would be helpful to have some sense of the precision of the provided means (e.g., standard deviations or confidence intervals) reported along side the means. It would also be helpful to explicitly define how variability is computed (e.g., standard deviation, variance, etc. and within subjects, over the same set of images, etc?). These details would significantly clarify the significance of the observed effects (or lack of effect in some cases).*

In Table 1, we added the standard deviations to all computed values. On page 11-12, we added detailed information on how trust variability, interpretation variability, and overall response variability have been computed.

*Potential Confounding Biases: I appreciate the thorough discussion of potential confounding factors. The thoughtful analysis of factors around domain and design conventions is a nice addition to the paper. Further, the lack of evidence that expertise has any influence on trust and interpretation raises fascinating questions about the influence of design knowledge and thinking in visualization. However, two potential confounds that may explain at least some of the observations arise from the design of the study itself rather than from the colormaps or visualization experience, namely familiarity/risk and confirmation bias. These concerns seem problematic given the reliance on a lack of result used to drive many conclusions in the paper.*

*R1-5 First, we expect that people are more familiar and comfortable with maps and space than with medical images. Medical images lead to both higher risk decisions and to less ability to personalize the data (see Peck et al, 2019), both of which are correlated with lower trust and confidence in an image and may provide an alternative explanation for the observed behaviors. This may lead to lower lay variability in the neuroscience images: they don’t feel they have the experience or authority to levy strong feelings or opinions and their response scales may be compressed as a consequence whereas geovis experts at least have familiarity and confidence in working with visualized data. It may also explain the higher criticality around brain death observed for neuroimaging experts over other populations.*

We thank the reviewer for this observation. Our data indeed allows estimating differences in familiarity and we do find significant differences with respect to the general experience with geo-images compared to neuro-images. Both lay people as well as geography experts have lower general expertise index values (a term that now is explained on page 10) for neuro-images compared to geo-images and these values are comparable for lay people and geography experts. We now discuss this potential confounding factor on page 22 by also citing the article of Peck et al. 2019.

*R1-6 Second, the question used in the geovis context appears like one that would be highly subject to confirmation bias. Most lay people have strong opinions on whether or not climate change is caused by human activities. As a result, confirmation bias may have a significant impact on responses to geovis questions compared to the neuroimaging questions, which focus on specific examples. This appears to play out in the high overall scores (mean of above 7 out of 8 for experts populations may indicate ceiling effects).*

Thank you for raising this point. We did indeed assess to what extent participants believe in a brain death claim or landscape death claim, as indeed both claims can and are being contested (i.e., see ongoing public debates on climate change and/or pro-live discussions). As outlined on page 20, however, differences with respect to these claims do not correlate with the recorded response variability, which speaks against a potential confirmation bias. Furthermore, we are unsure to what the remark regarding “ceiling effect” refers. In the absolute trust ratings (Table 2) no ceiling effects appear; the interpretation variability data is calculated differently (as not outlined) and no direct relation to the scale used (Likert scale 1-(9 can be made here. We therefore believe that this is not a general point of concern.

*R1-7 The discussion appears to point to that would invalidate this bias, “Our results also suggest that (c) potentially ideologically charged opinions related to the data context (in our case related to brain death as a death criterion or the degree to which human activities cause climate change) do not influence response variability, which is in line with our hypothesis.” However, it isn’t clear from the discussion of results (which primarily focus on expertise as relates to visualization) where the direct evidence of this division arises from. It would be helpful to the reader to more explicitly bridge this claim with the data itself.*

We have added more detail on this point, both in the result section on page 19 and in the discussion section on page 22. In particular, we clarified that some small (but indeed significant) correlations between trust and opinion exist. However, these do not play out in overall response variability.

*R1-8 While these alternative explanations do not invalidate the results, they do suggest limitations in considering the magnitude and outcomes of the provided results and for guiding future study. A revised manuscript should include an evidence-based discussion of how the general problem framing may invite specific kinds of biases and/or how those biases are mitigated by the infrastructure itself.*

As outlined above, we added our responses to these relevant points to the discussion section. We thank the reviewer for these opportunities for clarification. We thus added a paragraph on page 22 that summaries the limitations of the study.

*Grammatical Issues:*

*“each of the tree ranking tasks”—three*

*“that that (b) response”—extra “that”*

We have corrected those errors accordingly.

**Reviewer 2**

*R2-1 Of the 134 neuro email addresses, is there a possibility that some of these belong to a common person? That is, could some individual(s) have been represented multiple times in the responses, or were steps taken to somehow remove responses from an individual with multiple email addresses?*

We checked for duplicate email addresses before sending out participation invitations. We also checked for duplicates responses with the typical means for any type of online survey process, i.e., evaluation of IP addresses, comparing the similarity of response patterns, etc. We have added short notes on that point on page 6.

*R2-2 Of the 574 geographer email addresses, is there a possibility that some of these belong to a common person? That is, could some individuals have been represented multiple times in the responses, or were steps taken to somehow remove responses from an individual with multiple email addresses? The problem seems potentially more severe here (than with the neuro people) as there were also some addresses from ICA here.*

There is indeed a very small, albeit existent possibility that target people may have received the survey invitation through multiple channels (as already mentioned in the paper). However, as mentioned above we did check for duplicates. We are confident that we did not analyze duplicate data sets from an identical single respondent. We have added this clarification on p. 6. We cannot fully exclude the very small, but existent, possibility of malicious responders (i.e., the same person responding to the survey from different computers and in different ways). We do consider this probability for such malicious behavior to be very low, as no renumeration of any sort for participation was offered to the experts.

*R2-3 I wonder if some "master experts" who were contacted handed off their response to someone else. One way to test that would be to determine if any of the inputs came from email addresses different than those who were emailed (at least for the neuro people).*

Yes, we acknowledge this possibility, not only for neuro people. However, this would not be problematic for us, as we did assess in detail the experience of each respondent. We thus might have received answers of a person that may have less (or even more) experience than the targeted individual we had contacted by Email. As we applied pair-matching of experts with respect to their experience in the statistical analysis, this would thus not have affected our result.

*R2-4 How much were the Mechanical Turk people paid? How much were the domain experts paid?*

We have added this information on page 6. We thank the reviewer for this question.

*R2-5 The term "locked-in" in "Image Creation" is not well-known; a different term is needed.*

Thank you for helping us clarify this point. We mentioned on page 7 that all terms were explained in the questionnaire, and we also have added a short explanation of the “locked in” state in the paper.

*There is a typo in "Image Creation", Sentence 3: "graphic" -> "geographic".*

Thank you – the typo has been corrected.

*There are two grammar errors in "Image Creation": Sentence 6, "such as, residential" should be ", such as residential"; Sentence 7, "set is" should be "set was"*

Thank you – the errors have been corrected.

*R2-6 In "Structure of the online...", it should be mentioned if any of the Mechanical Turk participants were classified as experts? In addition, if any from the neuro or geography groups were classified as lay (or if none were), that should be mentioned.*

We thank the reviewer for this observation. We added this information on page 6.

*In "Structure of the online...", Paragraph 3, "likert" should be "Likert".*

Thank you – the typo has been corrected.

*R2-7 In "Structure of the online....", Paragraph 3, it should be made clear if the lay people answered all these questions.*

We clarified on page 9 that indeed all persons answered those questions.

*In "Structure of the online....", Paragraph 4, "more a consumer or a producer" should be "more consumers or producers".*

Thank you – the error has been corrected.

*In "Structure of the online....", Paragraph 4, what is "of main text" referring to? Is there another Figure 1 in another component that was not submitted?*

Thank you – the error has been corrected, there is indeed no other “Figure 1”.

*R2-8 In "Structure of the online....", Paragraph 6, Sentence 2, this point should also be made earlier--just before Paragraph 2 of "Structure of the online..." (or perhaps only made there).*

We now have made this point already in the fourth paragraph of this section.

*R2-9 Who were the 27 participants that tested questionnaire usability--were any of them the same ones that took part in the other parts of the study? Did any of the 27 that tested usability drop out due to the length of the survey completion?*

On page 11 we clarified that all usability testers were domain experts and that no data of the pretest have been used in the main study.

*R2-10 There is a sentence frag. just above Table 1 ("Specifically....").*

Thank you – the error has been corrected.

*R2-11 Figure 3 seems misleading as it aggregates the Table 1 information without regard to context. I'm not convinced of the conclusions related to such aggregated material...domain experts, especially if they have a long history in their domains, may tend to become very domain-centric and thus perhaps more likely to use their own training as they approach another domain, which may be a serious error for the other domain. I think the Figure 3 material should be de-aggregated, with the study then considering each context for each expert set, without considering "geo" as a whole, for example. Alternately, the focus could be just neuro experts on neuro data and geo experts just on geo data.*

We thank the reviewer for this important remark, as it points to the fact that the explanation for Figure 3 was not clear enough. We added in the paper that Figure 3 does not aggregate any data from Table 1, but compares the general suitability ratings of color scales (assessed by each of the expert groups)

*R2-12 In "Factors potentially," the focus suddenly shifts to "preference." I don't see where that comes from. Moreover, the Figure 3 material is suitability versus trust, NOT preference versus trust. There is a difference between something being suitable and something being preferred. This part of the paper needs some re-thought and re-work.*

We thank the reviewer for this important observation. We indeed did ask participants specifically about the “suitability” of one of the four presented scales to display the increase of a statistical parameter of any type (see survey question in the supplementary information). Being nonnative speakers we now realize that “suitability” cannot be equated with “preference”, thus we now correctly use the original term suitability systematically across the paper (in particular page 16). We report the original question as participants saw it in the questionnaire on page 10.

*R2-13 The current Figure 3 is not convincing to me that "geovis experts seem to trust the heated body color scale most" (statement just before the "Other variables" section) - the trust levels are pretty close for all. Either a statistical showing there is needed or the statement needs to be removed.*

Information on statistical trends has been added and the statement on page 17 has been adapted.

*R2-14 Table 2 has a low information-to-space ratio. The table should be reformatted in some way to be made much more compact - nearly every entry there is "ns" but the table takes up about 5% of the entire page length of the paper.*

Thank you for this comment. We agree, and make the table available in supplementary information.

*p. 19, "Our hypotheses" paragraph, line 5, "that that" should be "that".*

Thank you – the error has been corrected.

*R2-15 Ref. 2 is incomplete.*

We have replaced this reference by a very recently published paper (Crameri et al. 2020), which lead to a slight renumbering of the first 9 references.

*Lastly, a final positive remark: Next-to-last paragraph last sentence makes a very good point!*

We thank the reviewer for this positive remark.
