# Supplementary material for: Susceptibility of domain experts to color manipulation indicate a need for design principles in data visualization
Source: PLoS One. 2021 Feb 4;16(2):e0246479. doi: 10.1371/journal.pone.0246479 (PMC7861358; doi:10.1371/journal.pone.0246479)
Supplement: Attachment — Submitted filename: Response to Reviewers2.docx [file pone.0246479.s004.docx]

**Susceptibility of domain experts to color manipulation indicate a need for design principles in data visualization – Response to 2. Review**

We thank the reviewers for their detailed and constructive review. Below we respond to revision suggestions by reviewers in turn. All respective changes in the first revision iteration are marked in yellow, the revisions made in the second iteration are marked in green in the document “Revised Manuscript with Track Changes” (some smaller corrections in the yellow parts have not been marked in green).

**Reviewer 1**

*The definition of trust makes the rest of the conclusions a lot more logical and easier to follow. Given the definition, the results seem better supported compared with more conventional definitions. I wonder whether “trust” is really what this metric captures (it seems more like a measure of the validity of the conclusions), but so long as a clear definition is maintained, this is a minor point that I simply want to raise for the authors to consider as a potential opportunity for further clarity. It would similarly be useful to reiterate this definition when introducing these measures in the Structure section.*

We have further clarified our definition of “trust” on page 4 and reiterated this clarification on page 9.

*As another minor point for clarification, simply being blue-white-red does not necessarily mean the scale will be color-blind friendly (it depends on the internal variations in each of the blue and red sections). It’s worth noting if the scale has been externally confirmed to be colorblind safe (e.g., by the source document or by an external simulator). Most such encodings are, but explicit validity would be useful here.*

On page 8, we added detailed information that the bivariate blue-white-red scale has been checked for colorblind safety.

*Small issues:*

- *“In the methods part”—Methods section*
- *“Nine, respectively ten persons”—It seems like there’s an issue here: is it nine or ten? Also, were these participants removed or reclassified as they don’t fit the definition of a “lay” participant?*
- *“color vision deficiencies (no deficiency… (i.e., color blindness)”—Missing closing parentheses*
- *Some of the paragraphs in the discussion are quite long. For legibility, it may be worth breaking these into smaller pieces.*

We thank the reviewer for this observation. We have addressed all issues accordingly.

**Reviewer 2**

*This manuscript is a nice improvement on the initial submission. I recommend its acceptance. I did notice one small typo to fix - p. 22, line 3, "assessing" -> "assess"*

We thank the reviewer for this observation. We have corrected the typo accordingly.
